# Characteristics, service use and mortality of clusters of multimorbid patients in England: a population-based study

**DOI:** 10.1186/s12916-020-01543-8

**Published:** 2020-04-10

**Authors:** Yajing Zhu, Duncan Edwards, Jonathan Mant, Rupert A. Payne, Steven Kiddle

**Affiliations:** 1 MRC Biostatistics Unit, University of Cambridge, Cambridge, CB2 0SR UK; 2 Department of Public Health and Primary Care, Strangeways Research Laboratory, University of Cambridge, Worts’ Causeway, Cambridge, CB1 8RN UK; 3 Centre for Academic Primary Care, Population Health Sciences, Bristol Medical School, University of Bristol, Bristol, BS8 2PS UK

**Keywords:** Multimorbidity phenotype, Chronic conditions, Latent class analysis, Pattern recognition, Validation, Age stratification

## Abstract

**Background:**

Multimorbidity is associated with mortality and service use, with specific types of multimorbidity having differential effects. Additionally, multimorbidity is often negatively associated with participation in research cohorts. Therefore, we set out to identify clusters of multimorbidity patients and how they are differentially associated with mortality and service use across age groups in a population-representative sample.

**Methods:**

Linked primary and secondary care electronic health records contributed by 382 general practices in England to the Clinical Practice Research Datalink (CPRD) were used. The study included a representative set of multimorbid adults (18 years old or more, *N* = 113,211) with two or more long-term conditions (a total of 38 conditions were included). A random set of 80% of the multimorbid patients (*N* = 90,571) were stratified by age groups and clustered using latent class analysis. Consistency between obtained multimorbidity phenotypes, classification quality and associations with demographic characteristics and primary outcomes (GP consultations, hospitalisations, regular medications and mortality) was validated in the remaining 20% of multimorbid patients (*N* = 22,640).

**Results:**

We identified 20 patient clusters across four age strata. The clusters with the highest mortality comprised psychoactive substance and alcohol misuse (aged 18–64); coronary heart disease, depression and pain (aged 65–84); and coronary heart disease, heart failure and atrial fibrillation (aged 85+). The clusters with the highest service use coincided with those with the highest mortality for people aged over 65. For people aged 18–64, the cluster with the highest service use comprised depression, anxiety and pain. The majority of 85+-year-old multimorbid patients belonged to the cluster with the lowest service use and mortality for that age range. Pain featured in 13 clusters.

**Conclusions:**

This work has highlighted patterns of multimorbidity that have implications for health services. These include the importance of psychoactive substance and alcohol misuse in people under the age of 65, of co-morbid depression and coronary heart disease in people aged 65–84 and of cardiovascular disease in people aged 85+.

## Background

As a result of improved life expectancy and ageing populations, a growing number of individuals are living with multimorbidity, i.e. more than one long-term condition [1, 2]. Multimorbidity has been recognised as a global challenge for health care management [3], and it is estimated by the Health Foundation that 14 million individuals in England have multimorbidity, with over a third of these having more than four long-term conditions [4]. Patients with multimorbidity also account for the majority of primary care consultations, prescriptions and hospitalisations in the UK [5]. However, current clinical specialities, guidelines, quality improvement strategies and quality of care metrics are organised around single diseases [6], and treatments of multiple conditions are rarely coordinated, resulting in insufficient or even conflicting care [6, 7].

Patients with multimorbidity have a diverse range of diseases, needs and outcomes [4, 5, 8]. Identifying and characterising groups of multimorbid patients that share similar patterns of long-term conditions might facilitate an improvement in their healthcare. For example, such an approach might aid the development of effective strategies for early diagnosis and prevention of multimorbidity and allow for a better design and delivery of targeted interventions [1, 9]. Several systematic reviews have found common multimorbidity clusters involving cardiovascular-metabolic conditions, mental health and musculoskeletal disorders [10, 11]. However, existing evidence has important limitations. The two largest studies of specific multimorbidity groups in the UK have used UK Biobank data where participants are healthier (i.e. less multimorbid), smoke and drink less and are from less socioeconomically deprived areas than the overall population [12–14]. Second, most previous studies have focused on older populations (aged 60+); few have provided age-stratified clusters [10, 11, 15], leaving scarce evidence for the younger multimorbid population. Third, multimorbidity clusters composed of more than two conditions have not been well profiled mostly due to non-representative and smaller samples [1, 10, 11]. Fourth, there is substantial heterogeneity in the number of conditions considered (often less than 20) and in the statistical methods. Most studies focused on grouping diseases rather than patients, where each disease can only go into one cluster and so it is not straightforward to relate patients to outcomes in order to facilitate patient-centred policy-making [16]. Commonly used clustering methods were exploratory approaches such as factor analysis and hierarchical clustering [10, 17], where results were highly sensitive to the subjective choice of metrics [18]. Finally, the validity and generalisability of cluster solutions in new samples is important for decision-making but is often ignored in the current literature [10, 11].

This study aims to identify, validate and study the outcomes of age-stratified clusters of multimorbid adult patients in a large representative sample of UK patients. Towards this end, we used a comprehensive list of 38 long-term conditions [5] and a robust model-based probabilistic approach, latent class analysis [18].

## Methods

### Data source

Our analysis used the Clinical Practice Research Datalink (CPRD)-GOLD database where anonymised and longitudinal primary care clinical data are contributed by UK general (family) practices (GP) who use the Vision health record system [19]. CPRD has been validated to be representative of the UK population for age, sex and ethnicity [19, 20]. Patients’ GP records were linked with hospitalisation data (Hospital Episodes Statistics, HES), all-cause mortality data (Office for National Statistics, ONS) and area-based (1000–1500 people per area around patients’ home) socioeconomic deprivation data (Index of Multiple Deprivation, IMD); these linked data were available for approximately 75% of English practices in CPRD. The protocol for this study (16_057RA2) was approved scientifically and ethically by the CPRD Independent Scientific Advisory Committee.

### Study population

Data on a random selection of individuals were acquired from CPRD (the same individuals studied in Cassell et al. [5]). Patients aged 18 years and above with valid registered status in a practice with data classified by CPRD as “up-to-standard” in January 2012 [19] were included in the study. We chose the year 2012 to allow complete ascertainment of 5-year mortality. Additionally, we required that their practice allowed linkage to ONS, IMD and HES, resulting in the inclusion of only English practices.

### Patient and public involvement

There was no patient or public involvement in this study.

### Statistical software

Data analysis was performed in R 3.4.4. R package names are given in the following sections where appropriate (*in brackets and italics*); for example, memory-efficient packages were used to extract data for analysis in R (*ff, CALIBERdatamanage*). For transparency and reproducibility, all analysis scripts and code lists are available from https://github.com/Kiddle-group.

### Definition of patient characteristics, morbidities and outcomes

Morbidities in this study were defined as binary variables (present or not) based on the classification of LTCs in primary care developed by Barnett et al. [8]. This taxonomy attempted to include all conditions “likely to be chronic (defined as having significant impact over at least the most recent year) and with significant impact on patients in terms of need for chronic treatment, reduced function, reduced quality of life and risk of future morbidity and mortality”, and was developed for use in UK primary care electronic health record research and has been adapted for use in CPRD [5]. The specific definitions for each LTC are based on the UK Read code system and electronic prescription data coded using CPRD’s prodcode, giving a total of 38 LTCs (https://www.phpc.cam.ac.uk/pcu/cprd_cam/codelists/v11/). The LTCs used in this study largely match the only other large sample size UK multimorbidity cluster study [13].

Two sets of outcome variables related to service use and mortality were defined. NHS service utilisation or treatment burden was measured by three variables over the 12-month period after January 2012: primary care consultations (consultations with any clinician in the primary care team), the number of all-type hospitalisation spells (defined by discharge dates) and the count of regular medications (at least four prescriptions in a year by counting the unique British National Formulary (BNF) codes). All-cause mortality at 2 and 5 years was extracted from ONS data.

Patient characteristics that were considered in this study include gender, age groups (stratified into 18–44, 45–64, 65–84 and 85+ years) in 2012, last recorded pre-2012 body mass index (BMI), last recorded pre-2012 smoking status (current, never and ex-smokers) and socioeconomic deprivation measured by quintiles of IMD across the UK (1 for the least socioeconomically deprived quintile of areas and 5 for the most). Gender and age were determined in a straightforward manner from the CPRD-GOLD patient table. BMI and smoking status were extracted from the CPRD-GOLD clinical and additional tables using CPRD entity type 13 (BMI), CPRD entity type 4 (smoking status) and a smoking status Read code list from Jennifer Quint (Imperial College London) which is available at https://github.com/Kiddle-group.

### Statistical analysis

This study aims to identify clusters of multimorbid patients using patterns of co-existing long-term conditions. We used latent class analysis (LCA) (*poLCA*) to assign all patients to non-overlapping clusters (i.e. each patient is assigned to only one cluster) in a data-driven fashion [21, 22]. Compared to other exploratory clustering methods (e.g. factor analysis, hierarchical clustering [11, 23]), LCA is a model-based probabilistic clustering approach that is not sensitive to the rotation of factors and does not require any subjective choice of “distance measures” for multimorbidity patterns [18, 24]. This greatly enhances the reproducibility and stability of the latent class solutions. Clustering patients rather than diseases allows diseases to belong to multiple clusters and more naturally allows the characteristics and outcomes of clusters to be studied. As a result, each derived patient cluster has a unique and probabilistic multimorbidity phenotype profile where members do not necessarily need to have all conditions.

Guided by simulation studies [25], the optimal number of latent classes was decided using a combination of statistics (Bayesian Information Criteria (BIC), sample size-adjusted BIC, log-likelihood ratio test, entropy for classification quality) and clinical judgement. Within our datasets, conditions are present (i.e. recorded) or not by definition, and so missing data methods were not needed for cluster analysis. More details on a technical review of commonly used clustering methods, the LCA methodology and application of selection statistics are provided in Additional file 2: section 3.

To account for the different nature of multimorbidity clusters at different ages, four age strata (18–44, 45–64, 65–84, 85+ years) were chosen. We derived the cluster solution and performed post hoc statistical tests in a stratified (by age strata) random sample of the multimorbid population that contained 80% of the patients (i.e. training set). Separate LCAs were performed for each stratum, and each patient allocated to a single multimorbidity cluster. For ease of interpretation, clusters were labelled by their three most distinctive conditions whose difference in prevalence between cluster and age strata were the highest (see Additional file 2: table 1 for full details of conditions). To quantify the association between outcomes, multimorbidity clusters and patient demographics, generalised linear models were fitted (see Additional file 2: section 5). In the models for service use and health outcomes, the multimorbidity cluster with the lowest impact on the outcomes was taken as reference. For these models, individuals with missing data for last pre-2012 recording of smoking status or BMI represented a small percentage (< 5%) of the population, and so were excluded from the generalised linear models. Additional sensitivity analyses also using complete-case analysis were performed for the entire sample where the non-multimorbid patient group (which had higher missingness of smoking and BMI) was taken as reference (see Additional file 2: tables 16-19).

#### Assessment of the stability of morbidity clusters

To assess the stability of age-stratified multimorbidity clusters, LCAs were repeated in the remaining 20% of the population (i.e. test set), fixing the number of clusters to match that learned from the training set [26]. We employed three methods to indirectly validate our cluster solutions (a direct approach was not possible as clusters were unobserved). First, to check the consistency between disease profiles for 38 LTCs in the training and test sets, each cluster in the test set was matched (using two criteria for robustness) with a corresponding cluster in the training set. Matched cluster pairs were selected such that Jensen–Shannon distance [27] (JSD; a measure of the divergence between disease profiles) is the smallest and the bivariate Pearson’s correlation coefficient [28] (the degree to which two disease profiles co-vary) is the highest (Additional file 2: tables 4a, b). Second, entropy measures [25] (for classification quality) computed in the training and test sets were expected to be similar. Finally, stability was further assessed by observing in the training and test sets similar associations (in terms of size, direction and statistical significance) between clusters, patient demographics and outcome variables. For more details, see Additional file 2: section 4.

## Results

### Characteristics of the study population

A total of 391,669 patients were included in the study, of which 49% and 22% had none or only one long-term condition respectively (see Table 1 for patient demographics). Females, older individuals and those from areas of greater socioeconomic deprivation had a higher prevalence of multimorbidity.

**Table 1:** Demographic characteristics of the whole population (*N* = 391,669). For ordinal variables, median and first (Q1) and third (Q3) quartiles are reported. For categorical variables, counts and percentages are reported

Demographics	*N* (%)	No. of morbidities, median [Q1–Q3]	Multimorbid patients (%)
All patients	391,669 (100)	1 [0–2]	28.9
Gender			
Male	192,929 (49.3)	0 [0–2]	26.0
Female	198,740 (50.7)	1 [0–2]	31.7
Age group (years)			
18–24	32,007 (8.2)	0 [0–0]	4.7
25–34	60,501 (15.4)	0 [0–1]	7.9
35–44	68,688 (17.5)	0 [0–1]	13.1
45–54	74,734 (19.1)	0 [0–1]	20.5
55–64	60,323 (15.4)	1 [0–2]	34.4
65–74	49,427 (12.6)	2 [1–3]	53.6
75–84	31,262 (8.0)	3 [1–4]	73.6
85+	14,727 (3.8)	4 [2–5]	83.6
Socioeconomic status			
1 (least deprivation)	90,730 (23.2)	1 [0–2]	27.1
2	87,734 (22.4)	1 [0–2]	28.5
3	81,569 (20.8)	1 [0–2]	29.1
4	71,424 (18.2)	1 [0–2]	29.3
5 (greatest deprivation)	60,212 (15.4)	1 [0–2]	31.4

Independence test for demographics and multimorbidity

Gender vs multimorbidity: *χ*^2^ (1) = 1573.4, *p* < 0.001

Age group vs multimorbidity: *χ*^2^ (7) = 100,340.1, *p* < 0.001

IMD vs multimorbidity: *χ*^2^ (4) = 337.4, *p* < 0.001

Among the multimorbid patients (i.e. those with more than one long-term condition, *N* = 113,211, 29%), all unique combinations of conditions were less than 1% prevalent in the total population with the most prevalent 20 containing only pairs of conditions (Additional file 2: table 2). This together with the large number of unique combinations of conditions (Additional file 2: table 3) indicated that multimorbidity patterns were highly heterogeneous. Stratified by age strata, Table 2 shows that multimorbidity in the younger population (18–44) was more common in areas with greater socioeconomic deprivation while for older groups, the pattern is reversed.

**Table 2:** Characteristics of multimorbid patients (*N* = 113,211). For continuous and ordinal variables, median and first (Q1) and third (Q3) quartiles are reported. For categorical variables, counts and percentages are reported

Demographic characteristics	Across age strata (*N* = 113,211)	Age 18–44 years (*N* = 15,306)	Age 45–64 years (*N* = 36,097)	Age 65–84 years (*N* = 49,494)	Age 85+ years (*N* = 12,314)
Female (%)	63,072 (56)	9422 (62)	19,690 (55)	25,952 (52)	8008 (65)
No. of morbidities, median [Q1–Q3]	3 [2–4]	2 [2–3]	2 [2–3]	3 [2–4]	4 [3–6]
Age, median [Q1–Q3]	66 [53–77]	37 [30–41]	56 [51–61]	74 [69–79]	89 [86–91]
BMI					
Median [Q1–Q3]	27 [24–31]	26 [23–31]	28 [25–33]	27 [24–31]	25 [22–28]
Missing (%)	5788 (5)	1571 (10)	1462 (4)	1540 (3)	1215 (10)
Smoking status (%)					
Current smoker	21,586 (19)	5641 (37)	9140 (25)	6160 (12)	645 (5)
Never smoker	55,145 (49)	6789 (45)	17,040 (47)	24,042 (49)	7274 (59)
Ex-smoker	36,265 (32)	2821 (18)	9869 (27)	19,251 (39)	4324 (35)
Missing	215 (0.19)	55 (0.36)	48 (0.13)	41 (0.08)	71 (0.58)
Index of multiple deprivation in quintiles (%)					
1 (least deprivation)	24,624 (22)	2647 (17)	7333 (20)	11,722 (24)	2922 (24)
2	25,027 (22)	2738 (18)	7668 (21)	11,667 (24)	2954 (24)
3	23,700 (21)	2969 (19)	7352 (20)	10,612 (21)	2767 (22)
4	20,934 (18)	3316 (22)	6862 (19)	8645 (17)	2111 (17)
5 (greatest deprivation)	18,926 (17)	3636 (24)	6882 (19)	6848 (14)	1560 (13)

### Multimorbidity clusters and outcomes

For ease of reference, we refer to each cluster by its lead or key conditions (i.e. one or three conditions, respectively, whose cluster-specific prevalence is highest, and higher than their overall prevalence in their respective age group).

These clusters differ across age strata, both in terms of the number of clusters per strata and main components within each cluster (Table 3 and Additional file 2: figures 1-4). The association between multimorbidity clusters and outcomes (service use and mortality) remained significant (*p* < 0.01 in almost all clusters) after stratifying by age strata, controlling for socioeconomic deprivation, BMI and smoking behaviour (Additional file 2: tables 8–11). Results for the distribution of outcomes (i.e. median, interquartile range (IQR)) are shown in Table 4. Covariate-adjusted incidence rate ratios (aIRRs) for service utilisation and odds ratios (OR) for mortality derived from generalised linear models are available in Additional file 2: tables 8–15 (adjusted covariates were gender, socioeconomic deprivation, smoking status, BMI and age).

**Table 3:** Descriptions of the derived clusters of multimorbid patients for each age strata. Clusters are ordered by sizes from the largest to the smallest. Key conditions are the three estimated to be most distinctive in the cluster (where the difference between within-cluster prevalence and prevalence in age strata is the largest). For the number of morbidities, median and first (Q1) and third (Q3) quartiles are reported. For other categorical variables, percentages are reported. Greater deprivation denotes top 40% of IMD (categories 4 and 5)

Three key conditions (prevalence)		Patients (%)	No. of morbidities (median [Q1–Q3])	Female (%)	Greater deprivation (%)	Current smokers (%)
Lead condition (%)	Subsidiary conditions (%)					
Age 18–44 years						
Depression (100%)	Anxiety (41%), pain (31%)	32	2 [2–3]	66	50	46
Pain (36%)	Hearing loss (30%), hypertension (23%)	23	2 [2–3]	52	46	27
Asthma (100%)	IBS (26%), depression (20%)	20	2 [2–3]	63	41	29
IBS (100%)	Depression (29%), hearing loss (21%)	18	2 [2–3]	77	37	28
PSM (75%)	Alcohol (42%), depression (24%)	7	2 [2–3]	28	63	76
Age 45–64 years						
Hypertension (76%)	Diabetes (37%), pain (25%)	37	2 [2–3]	42	38	20
IBS (40%)	Hearing loss (29%), pain (28%)	24	2 [2–3]	64	29	20
Depression (93%)	Pain (53%), anxiety (31%)	22	3 [2–5]	68	46	35
Asthma (100%)	Pain (24%), COPD (16%)	12	2 [2–3]	61	35	20
Alcohol (62%)	PSM (42%), pain (28%)	4	3 [2–4]	31	57	63
Age 65–84 years						
Hypertension (100%)	Diabetes (31%), pain (27%)	41	3 [2–4]	54	30	10
Hearing loss (40%)	Prostate disorder (21%), IBS (3%)	22	3 [2–4]	48	25	9
Depression (56%)	Pain (56%), anxiety (23%)	14	4 [3–5]	72	33	15
CHD (54%)	Diabetes (32%), atrial fibrillation (29%)	11	4 [3–5]	30	33	12
COPD (57%)	Asthma (49%), pain (33%)	8	3 [2–5]	50	40	24
Pain (81%)	CHD (53%), depression (45%)	5	7 [7–9]	54	43	16
Age 85+ years						
Hypertension (72%)	Hearing loss (39%), diabetes (18%)	58	3 [2–4]	61	30	5
Pain (64%)	Depression (41%), constipation (24%)	23	5 [4–6]	80	30	5
CHD (61%)	Atrial fibrillation (53%), heart failure (49%)	11	7 [6–8]	60	30	4
Asthma (48%)	COPD (48%), pain (44%)	8	5 [4–6]	59	30	8

*IBS* irritable bowel syndrome, *PSM* psychoactive substance misuse (not alcohol), *CHD* coronary heart disease, *COPD* chronic obstructive pulmonary disease

**Table 4:** Mortality and health service utilisation by patient clusters in each age stratum. Clusters are ordered by the highest to the lowest mortality. The non-multimorbid cluster contains patients with zero or only one long-term condition. The number of GP consultations, hospitalisations and repeat prescriptions (by counting the number of unique BNF codes that were in repeated prescriptions at least four times) are measured in 1 year after January 2012. Both mean and median are reported because they highlight different aspects of skewed distributions, especially in relation to hospitalisations and prescriptions

Three key conditions (prevalence)		2-year mortality (%)	5-year mortality (%)	No. of GP contacts in a year (mean, median [Q1–Q3])	No. of hospitalisations in a year (mean, median [Q1–Q3])	No. of unique medicine on repeat prescriptions in a year (mean, median [Q1–Q3])
Lead condition (%)	Subsidiary conditions (%)					
Age 18–44 years						
PSM (75%)	Alcohol (42%), depression (24%)	1.8	3.9	10.7, 7 [1–16]	0.4, 0 [0–0]	1.3, 0 [0–2]
Pain (36%)	Hearing loss (30%), hypertension (23%)	1.0	2.7	11.9, 9 [3–17]	0.6, 0 [0–0]	2.5, 1 [0–4]
Depression (100%)	Anxiety (41%), pain (31%)	0.9	1.8	14.5, 12 [5–20]	0.4, 0 [0–0]	2.4, 2 [0–3]
Asthma (100%)	IBS (26%), depression (20%)	0.2	0.6	11.3, 9 [4–16]	0.4, 0 [0–0]	1.9, 1 [0–3]
IBS (100%)	Depression (29%), hearing loss (21%)	0.2	0.4	10.6, 8 [3–15]	0.4, 0 [0–0]	1.2, 0 [0–2]
Non-multimorbid		0.1	0.2	3.7, 1 [0–5]	0.2, 0 [0–0]	0.2, 0 [0–0]
Age 45–64 years						
Alcohol (62%)	PSM (42%), pain (28%)	4.5	12.5	10.7, 8 [2–16]	0.5, 0 [0–1]	2.4, 1 [0–4]
Depression (93%)	Pain (53%), anxiety (31%)	2.4	5.8	16.7, 14 [7–23]	0.6, 0 [0–1]	5.1, 4 [2–7]
Hypertension (76%)	Diabetes (37%), pain (25%)	1.6	4.4	11.5, 9 [4–16]	0.5, 0 [0–0]	4.1, 4 [1–6]
IBS (40%)	Hearing loss (29%), pain (28%)	1.3	3.0	10.5, 8 [3–15]	0.5, 0 [0–0]	2.0, 1 [0–3]
Asthma (100%)	Pain (24%), COPD (16%)	1.0	2.7	12.6, 10 [5–17]	0.4, 0 [0–0]	3.4, 3 [1–5]
Non-multimorbid		0.4	1.3	4.4, 2 [0–6]	0.2, 0 [0–0]	0.5, 0 [0–0]
Age 65–84 years						
Pain (81%)	CHD (53%), depression (45%)	16.2	39.2	26.3, 23 [14–35]	1.6, 1 [0–2]	10.7, 11 [8–14]
CHD (54%)	Diabetes (32%), atrial fibrillation (29%)	11.3	28.8	16.8, 14 [6–24]	1.1, 0 [0–1]	5.9, 6 [3–8]
COPD (57%)	Asthma (49%), pain (33%)	9.2	25.5	15.7, 13 [6–22]	0.8, 0 [0–1]	5.5, 5 [2–8]
Depression (56%)	Pain (56%), anxiety (23%)	8.4	20.9	17.3, 14 [8–23]	0.8, 0 [0–1]	5.9, 6 [3–8]
Hypertension (100%)	Diabetes (31%), pain (27%)	4.7	13.2	12.4, 10 [4–17]	0.6, 0 [0–1]	4.5, 4 [2–7]
Hearing loss (40%)	Prostate disorder (21%), IBS (3%)	4.4	11.1	13.2, 11 [5–19]	0.7, 0 [0–1]	3.3, 3 [1–5]
Non-multimorbid		1.9	6.6	6.4, 4 [0–9]	0.3, 0 [0–0]	1.2, 0 [0–2]
Age 85+ years						
CHD (61%)	Atrial fibrillation (53%), heart failure (49%)	37.7	70.8	21.9, 18 [9–31]	1.5, 1 [0–2]	8.0, 8 [5–11]
Pain (64%)	Depression (41%), constipation (24%)	31.1	62.9	17.3, 15 [7–24]	0.8, 0 [0–1]	6.7, 7 [4–10]
Asthma (48%)	COPD (48%), pain (44%)	28.0	56.5	19.6, 17 [9–27]	1.1, 0 [0–2]	6.9, 7 [4–10]
Hypertension (72%)	Hearing loss (39%), diabetes (18%)	20.9	49.5	13.0, 10 [2–19]	0.8, 0 [0–1]	4.1, 4 [0–6]
Non-multimorbid	–	13.4	36.0	6.7, 3 [0–10]	0.4, 0 [0–0]	1.3, 0 [0–2]

*IBS* irritable bowel syndrome, *PSM* psychoactive substance misuse (not alcohol), *CHD* coronary heart disease, *COPD* chronic obstructive pulmonary disease

#### Age strata: 18–44 years old

Five clusters were uncovered in the 18–44 age strata (Additional file 2: figures 1 & 5), whose lead conditions were depression (the most common cluster, 32% of patients in strata), pain (23%), asthma (20%), irritable bowel syndrome (18%) and psychoactive substance misuse (7%). Those in the cluster whose three key conditions were depression (within-cluster prevalence 100%), anxiety (41%) and pain (31%) were found to have the highest use of primary care consultations (median 12 [IQR 5–20] in a year). This cluster had an aIRR of primary care consultations of 1.35 (95% confidence interval (CI) 1.28–1.43) in comparison with the cluster with the lowest service use and mortality (whose lead condition was irritable bowel syndrome).

Those in the cluster whose three key conditions were pain (36%), hearing loss (30%) and hypertension (23%) were found to have the highest hospital admission rates (an average of 0.6 visits in a year) and the highest count of regular medicines (median 1 [IQR 0–4] unique drug classes in a year). This corresponded to an aIRR for hospitalisations of 1.04 [95% CI 0.90–1.20] and an aIRR for regular medicines of 1.87 [95% CI 1.74–2.02] relative to the cluster with the lowest service use and mortality.

The highest mortality in this age range was found in the least prevalent (7%) multimorbidity cluster whose three key conditions were psychoactive substance misuse (75%), alcohol problems (42%) and depression (24%) (3.9% mortality in 5 years). This level of mortality was 18 times higher than that of individuals in the same age range without multimorbidity (0.2%). This cluster was predominantly male (72%), came from socioeconomically deprived areas (63% from the most deprived 40% of UK areas) and with high smoking rates (76% current smokers).

#### Age strata: 45–64 years old

In the 45–64 age strata, LCA revealed five clusters (Additional file 2: figures 2 & 6), whose lead conditions were hypertension (the most common cluster, 37% of patients in strata), irritable bowel syndrome (24%), depression (22%), asthma (12%) and alcohol problems (4%). Those in the cluster whose three key conditions were depression (93%), pain (53%) and anxiety (31%) had the highest number of primary care consultations (median 14 [IQR 7–23] in a year, aIRR = 1.52 [95% CI 1.47–1.58]), hospital admission rates (an average of 0.6 visits in a year, aIRR = 1.31 [95% CI 1.31–1.44]) and regular medications (median 4 [IQR 2–7], aIRR = 2.37 [95% CI 2.29–2.46]). As in the younger age strata, the least prevalent multimorbidity cluster (4%) had the highest death rate (13% in 5 years, OR = 1.08 [95% CI 1.07–1.10]); its key conditions were alcohol problems (62%), psychoactive substance misuse (42%) and pain (28%). Again, this cluster was characterised by being typically male smokers from areas of high socioeconomic deprivation. Pain as a co-morbidity was represented in all the clusters in this age group.

#### Age strata: 65–84 years old

Six clusters were found in the 65–84 age strata (Additional file 2: figures 3 & 7), whose lead conditions were hypertension (the most common cluster, 41% of patients in strata), hearing loss (22%), depression (14%), coronary heart disease (11%), chronic obstructive pulmonary disease (8%) and pain (5%). The least prevalent multimorbidity cluster (5%), whose key conditions were pain (81%), coronary heart disease (53%) and depression (45%), had the highest use of primary care consultations (median 23 [IQR 14–35] in a year, aIRR = 1.92 [95% CI 1.82–2.02]), hospital admissions (an average of 1.6 visits in a year, aIRR = 2.15 [95% CI 1.94–2.40]), regular medicines (median 11 [IQR 8–14], aIRR = 2.88 [95% CI 2.88–3.00]) and death rates (39% mortality in 5 years).

#### Age strata: above 85 years old

The 85+ age stratum was composed of four clusters (Additional file 2: figures 4 & 8), whose lead conditions were hypertension (the most common, 58% of patients in strata), pain (23%), heart failure (11%) and asthma (8%). The majority of patients (58%) fitted within a cluster whose key conditions were hypertension (72%), hearing loss (39%) and diabetes (18%). The cluster with the majority of patients had the lowest mortality (50% 5-year mortality), as well as the least number of conditions (median 3 [IQR 2–4] morbidities), and the least health care utilisation (roughly half the GP contacts, hospitalisations and regular medicines of the cluster whose lead condition was “coronary heart disease”). The cluster with the highest mortality, GP contact, hospitalisations and repeat prescriptions comprised a trio of cardiac conditions: coronary heart disease, atrial fibrillation and heart failure.

### Validation of cluster morbidity profiles

As well as validating the clusters by their association with patient characteristics and outcomes, the similarity of multimorbidity clusters was compared between the training set (80% of patients, *N* = 90,571) and the test set (the other 20% of patients, *N* = 22,640). Results are summarised below and given in full in Additional file 2: section 4. Measures of cluster quality (i.e. entropy) were found to be consistent between the training and test sets.

As the training set contained more disease patterns, the derived clusters were more comprehensive. The test set (with fewer patients) contained fewer disease patterns, and therefore, we expected the derived clusters to be a subset of those in the training set. Indeed, validation of cluster profiles showed that every cluster in the test set found a match in the training set. Some clusters were particularly robust (had the smallest JSD and the highest Pearson’s correlation coefficient), for instance, those in the largest age strata (65–84 age strata, *N* = 49,494), and clusters whose lead condition was depression, psychoactive drug misuse or alcohol problems. A cluster with a less clear match had the lead condition asthma in the 18–44 age strata.

## Discussion

### Summary of results and comparison with other studies

This study identified and validated clusters of multimorbid patients using a novel patient-centred approach. In summary, we identified 20 patient clusters across four age strata. In the younger age-strata (18–44; 45–64), the clusters with the highest mortality (18 times higher than the non-multimorbid group in 18–44-year olds) comprised psychoactive substance abuse in combination with alcohol problems. The clusters with the most contact with general practice in people aged under 65 comprised depression, anxiety and pain. In 65–84-year olds, the cluster with the highest mortality and highest health service use (GP contact, hospitalisations, repeat prescriptions) comprised pain, coronary heart disease and depression, and in people aged 85 or over, it comprised heart failure, coronary heart disease and atrial fibrillation. The most common cluster in 18–44-year olds was centred around depression, but in all other age groups, they were centred around hypertension. In the oldest age group, this hypertension-centred cluster was associated with the best survival and lowest health service use among multimorbid patients. Pain featured in 13 of the clusters.

In this study, unlike most previous analyses of multimorbidity, we have defined novel clusters in terms of patients rather than diseases [10, 11]. These clusters have practical implications for service delivery, by supporting a move away from healthcare for individual diseases towards the care of individuals with clusters of conditions, and thus helping to reduce treatment burden [29]. The high mortality of the cluster of psychoactive substance and alcohol misuse warrants attention. The descriptive epidemiology of this cluster (male, smoker, under age 65, relatively low service use, from areas of greater socioeconomic deprivation) supports the need for better integration of substance misuse services within primary care and need to provide improved access to holistic management including for physical health [30]. Conversely, we found that the commonest cluster in people aged 85 and over (58% of patients with multimorbidity in this age group) was associated with the least health service use and the lowest mortality (Table 4). This gives a more nuanced perspective on the association of multimorbidity with age that has been widely reported [8], in that it suggests that in the oldest age group, multimorbidity per se may be less important, although numerically most common. We hypothesise that this is due to a survivor effect, i.e. individuals with more fatal multimorbidity often do not reach this age. Our age-stratified approach also enables different patterns of co-morbidity to be identified. Thus, in younger age groups, clusters focused around mental health are associated with most GP contact; in people aged 65–84, a cluster of mental health and coronary heart disease is associated with most GP contact, and other indicators of health service use, whereas in people aged over 85, the cluster representing most health service contact is dominated by cardiovascular conditions.

In terms of relative importance of single conditions within multimorbid clusters, the predominance of mental health conditions and hypertension has been identified in previous work [10, 11, 13, 31]. Mental health conditions are recognised as having a major impact on health service use over and above physical problems [32], and our findings emphasise the importance of ensuring psychological needs are given equal priority to physical ones in those clusters of patients where mental health problems are prevalent [33]. A novel finding in our work is the inclusion of pain in many of the clusters we identified. This aligns with studies that confirm chronic pain is widespread and emphasises the need to provide integrated pain management services to address the potential adverse impact on health service use and both physical and social functioning [34, 35].

### Strengths and limitations

The robust identification of such clusters would not have been possible without the novel use of representative data reflecting real-world patterns of multimorbidity, age stratification, patient-level clustering (not requiring all patients to have identical lists of conditions) and validation with held-out data. This is the largest-scale application of age-stratified latent class analysis to multimorbidity, both in patient numbers (above 100,000) and the number of conditions (38) [11]. By including younger patients and stratifying by age, we see how multimorbidity clusters differ over the lifecourse. Combining this with the release of reproducible analysis scripts is an approach which we recommend for future multimorbidity clustering efforts. Our systematic approach including all 38 conditions from Barnett et al. [8], age stratification, clustering and outcomes, was necessary to handle the complexity of multimorbidity in healthcare.

This study suffers from typical limitations of electronic health record research in that they rely on routine coding within the healthcare system including residual confounding and variable CPRD data quality. Wherever practically feasible we have taken steps to address these, e.g. the careful design of codelists, relying on variables with low missingness and adjusting for key variables. Some relevant information, such as disease severity, was not available for the majority of diseases and so was not modelled. This may affect the association of disease with characteristics and outcomes. Given the observational nature of this data, some residual confounding such as this is inevitable, and so we caution that the relationship between clusters, patient demographics and outcomes should not be interpreted causally.

While the clustering approach used (LCA) is a robust probabilistic approach, results may differ subtly if other approaches are used. Validation of latent clusters also requires further research where a larger sample size for the test set, perhaps from another database or country, can strengthen the validation. We notice that in every age strata, there was a cluster whose lead condition (pain, irritable bowel syndrome, hearing loss and asthma respectively) had a within-cluster prevalence of less than 50%, suggesting that they are less distinctive than the other clusters. It is also interesting that they are often the clusters with the lowest mortality. While these were validated in the test set, it may be that bigger datasets are required to split these into more distinct and interpretable clusters. Despite this, given the large and representative sample, the consistency of results both internally, across age strata and with existing literature, we are confident in our main results. Finally, multimorbidity evolves over time, but we only use longitudinal data to extract conditions in 2012 and service use and mortality outcomes.

## Conclusion

These multimorbidity clusters highlight major targets for public health and healthcare, giving a more nuanced understanding of multimorbidity than the work of Barnett et al. [8] which rely more on simple disease counts. The 18-fold higher mortality of younger multimorbid patients with psychoactive substance misuse is a clear case of an unmet need. Improving outcomes for this neglected patient group is likely to be feasible given that their risk factors (drug use, smoking, deprivation) are potentially amenable to intervention. Conversely, the fact that the majority of older multimorbid patients have relatively low service use and mortality has implications for the design of health services. More generally, the fact that chronic pain is a key feature of many multimorbidity clusters suggests that it is important to manage pain within the context of multimorbidity rather than in its own right. Similarly, our findings add to the evidence showing the importance of mental health in multimorbid patients, justifying the push for parity of physical and mental health within the healthcare system.

While patients with multimorbidity account for an ever-increasing proportion of healthcare need and provision [1, 4, 5, 7, 8], no existing interventions have shown convincing evidence of benefit in improving important outcomes [9, 36]. Our findings fit with the suggestion from Salisbury et al. [9] that one reason for the failure of previous interventions is that multimorbidity is heterogeneous, with very different diseases, needs and outcomes in different groups of patients. Our findings support the proposal that interventions to improve outcomes in multimorbidity may be more appropriately targeted on distinct types, and we have systematically highlighted groups of patients where tailored approaches could be attempted.

## Supplementary information

**Additional file 1.** ISAC protocol (16_057RA2)

**Additional file 2.** Supplementary

**Additional file 3.** STROBE checklist

## Data Availability

The Clinical Practice Research Datalink (CPRD) is an electronic healthcare record database open to all researchers. Researchers can apply to access CPRD data and, if successful, can access the data of their choosing. The CPRD charges researchers and other organisations to access this data. The data that support the findings of this study are available from the Clinical Practice Research Datalink (CPRD), but restrictions apply to the availability of these data, which were used under licence for the current study, and so are not publicly available. Data are however available from the authors upon reasonable request and with permission of Clinical Practice Research Datalink.

## References

[1] AcademyofMedicalSciences (2018). Multimorbidity: a priority for global health research.

[2] WHO:WorldHealthOrganization. Multimorbidity. 2016. https://apps.who.int/iris/handle/10665/252275.

[3] Wallace E, Salisbury C, Guthrie B, Lewis C, Fahey T, Smith SM (2015). Managing patients with multimorbidity in primary care. BMJ.

[4] Stafford M, Steventon A, Thorlby R, Fisher R, Turton C, Deeny S (2018). Briefing: understanding the health care needs of people with multiple health conditions.

[5] Cassell A, Edwards D, Harshfield A, Rhodes K, Brimicombe J, Payne R (2018). The epidemiology of multimorbidity in primary care: a retrospective cohort study. Br J Gen Pract.

[6] Chaplin K, Bower P, Man M-S, Brookes ST, Gaunt D, Guthrie B (2018). Understanding usual care for patients with multimorbidity: baseline data from a cluster-randomised trial of the 3D intervention in primary care. BMJ Open.

[7] National Guideline Centre (UK) (2016). Multimorbidity: assessment, prioritisation and management of care for people with commonly occurring multimorbidity.

[8] Barnett K, Mercer SW, Norbury M, Watt G, Wyke S, Guthrie B (2012). Epidemiology of multimorbidity and implications for health care, research, and medical education: a cross-sectional study. Lancet.

[9] Salisbury C, Man M-S, Bower P, Guthrie B, Chaplin K, Gaunt DM (2018). Management of multimorbidity using a patient-centred care model: a pragmatic cluster-randomised trial of the 3D approach. Lancet.

[10] Prados-Torres A, Calderón-Larrañaga A, Hancco-Saavedra J, Poblador-Plou B, van den Akker M (2014). Multimorbidity patterns: a systematic review. J Clin Epidemiol.

[11] Busija L, Lim K, Szoeke C, Sanders KM, McCabe MP. Do replicable profiles of multimorbidity exist? Systematic review and synthesis. Eur J Epidemiol. 2019. 10.1007/s10654-019-00568-5. 10.1007/s10654-019-00568-5 31624969

[12] Jani BD, Hanlon P, Nicholl BI, McQueenie R, Gallacher KI, Lee D (2019). Relationship between multimorbidity, demographic factors and mortality: findings from the UK Biobank cohort. BMC Med.

[13] Zemedikun DT, Gray LJ, Khunti K, Davies MJ, Dhalwani NN (2018). Patterns of multimorbidity in middle-aged and older adults: an analysis of the UK Biobank data. Mayo Clin Proc.

[14] Biobank (2007). Access matter: representativeness of the UK Biobank resource.

[15] Poblador-Plou B, van den Akker M, Vos R, Calderón-Larrañaga A, Metsemakers J, Prados-Torres A (2014). Similar multimorbidity patterns in primary care patients from two European regions: results of a factor analysis. PLoS One.

[16] Collerton J, Jagger C, Yadegarfar ME, Davies K, Parker SG, Robinson L (2016). Deconstructing complex multimorbidity in the very old: findings from the Newcastle 85+ study. Biomed Res Int.

[17] Ahlqvist E, Storm P, Käräjämäki A, Martinell M, Dorkhan M, Carlsson A (2018). Novel subgroups of adult-onset diabetes and their association with outcomes: a data-driven cluster analysis of six variables. Lancet Diabetes Endocrinol.

[18] Bartholomew DJ, Steele F, Galbraith J, Moustaki I. Analysis of multivariate social science data. London: Chapman and Hall/CRC; 2008.

[19] Herrett E, Gallagher AM, Bhaskaran K, Forbes H, Mathur R, van Staa T (2015). Data resource profile: Clinical Practice Research Datalink (CPRD). Int J Epidemiol.

[20] Mathur R, Bhaskaran K, Chaturvedi N, Leon DA, VanStaa T, Grundy E (2013). Completeness and usability of ethnicity data in UK-based primary care and hospital databases. J Public Health (Bangkok).

[21] Larsen FB, Pedersen MH, Friis K, Glümer C, Lasgaard M (2017). A latent class analysis of multimorbidity and the relationship to socio-demographic factors and health-related quality of life. A national population-based study of 162,283 Danish adults. PLoS One.

[22] Hall M, Dondo TB, Yan AT, Mamas MA, Timmis AD, Deanfield JE (2018). Multimorbidity and survival for patients with acute myocardial infarction in England and Wales: latent class analysis of a nationwide population-based cohort. PLoS Med.

[23] Prados-Torres A, Poblador-Plou B, Calderón-Larrañaga A, Gimeno-Feliu LA, González-Rubio F, Poncel-Falcó A (2012). Multimorbidity patterns in primary care: interactions among chronic diseases using factor analysis. PLoS One.

[24] Dendukuri N, Schiller I, de Groot J, Libman M, Moons K, Reitsma J, et al. Concerns about composite reference standards in diagnostic research. BMJ. 2018;360:j5779. 10.1136/bmj.j5779 29348126

[25] Nylund KL, Asparouhov T, Muthén BO (2007). Deciding on the number of classes in latent class analysis and growth mixture modeling: a {Monte Carlo} simulation study. Struct Equ Model A Multidiscip J.

[26] Finch WH, Bronk KC (2011). Conducting confirmatory latent class analysis using Mplus. Struct Equ Model A Multidiscip J..

[27] Lin J (1991). Divergence measures based on the Shannon entropy. IEEE Trans Inf Theory.

[28] Altman DG. Practical statistics for medical research. London: CRC press; 1990.

[29] May CR, Montori VM, Mair FS. We need minimally disruptive medicine. BMJ (Online). 2009;11:b2803. 10.1136/bmj.b2803 19671932

[30] Lawson E. Management of opioid addiction in primary care: a pragmatic approach prioritising wellbeing not ideology. Br J Gen Pract. 2013;63:231–2. 10.3399/bjgp13X665396 PMC3635548 23643204

[31] Déruaz-Luyet A, N’Goran AA, Senn N, Bodenmann P, Pasquier J, Widmer D, et al. Multimorbidity and patterns of chronic conditions in a primary care population in Switzerland: a cross-sectional study. BMJ Open. 2017;7:e013664. 10.1136/bmjopen-2016-013664 PMC5734197 28674127

[32] Payne RA, Abel GA, Guthrie B, Mercer SW. The effect of physical multimorbidity, mental health conditions and socioeconomic deprivation on unplanned admissions to hospital: a retrospective cohort study. CMAJ. 2013;185:E221–8. 10.1503/cmaj.121349 PMC3602270 23422444

[33] Das P, Naylor C, Majeed A. Bringing together physical and mental health within primary care: a new frontier for integrated care. J R Soc Med. 2016;109:364–6. 10.1177/0141076816665270 PMC5066536 27729592

[34] Breivik H, Collett B, Ventafridda V, Cohen R, Gallacher D. Survey of chronic pain in Europe: prevalence, impact on daily life, and treatment. Eur J Pain. 2006;10:287–333. 10.1016/j.ejpain.2005.06.009 16095934

[35] Bruggink L, Hayes C, Lawrence G, Brain K, Holliday S. Chronic pain: overlap and specificity in multimorbidity management. Aust J Gen Pract. 2019;48:689–92. 10.31128/AJGP-06-19-4966 31569314

[36] Smith SM, Wallace E, O’Dowd T, Fortin M. Interventions for improving outcomes in patients with multimorbidity in primary care and community settings. Cochrane Database Syst Rev. 2016;14:CD006560. 10.1002/14651858.CD006560.pub3 PMC6703144 26976529

