# Supplementary material for: Characteristics, service use and mortality of clusters of multimorbid patients in England: a population-based study
Source: BMC Med. 2020 Apr 10;18:78. doi: 10.1186/s12916-020-01543-8 (PMC7147068; doi:10.1186/s12916-020-01543-8)
Supplement: Supplementary file 1 — Additional file 1. ISAC protocol (16_057RA2) [file 12916_2020_1543_MOESM1_ESM.docx]

PROTOCOLS FOR RESEARCH USING THE CLINICAL PRACTICE RESEARCH DATALINK (CPRD)

| ISAC use only:  Protocol Number  Date submitted | .............................  ............................. | **IMPORTANT**  **If you have any queries, please contact ISAC Secretariat:** ISAC[@cprd.com](mailto:Annalisa.Rubino@gprd.com) |
| --- | --- | --- |

| **Section A: The study** | | |
| --- | --- | --- |
| **Study Title**  Descriptive Epidemiology of Multimorbidity in Primary Care: A Cross-Sectional Study | | |
| 1. **Has any part of this research proposal or a related proposal been previously submitted to ISAC?**   Yes No  *If Yes, please provide previous protocol numbers*: | | |
| 1. **Has this protocol been peer reviewed by another Committee? (e.g. grant award or ethics committee)**   Yes No  *If Yes, please state the name of the reviewing Committee(s) and provide an outline of the review process and outcome:* | | |
| 1. **Type of Study** (please tick all the relevant boxes which apply)   Adverse Drug Reaction/Drug Safety Drug Utilisation  Disease Epidemiology  Drug Effectiveness  Pharmacoeconomics  Methodological  Health/Public Health Services Research  Post-authorisation Safety  **Other^*^**  ^*^Please specify the type of study in the lay summary | | |
| 1. **This study is intended for** (please tick all the relevant boxes which apply)**:**   Publication in peer reviewed journals  Presentation at scientific conference  Presentation at company/institutional meetings  Regulatory purposes  Other | | |
| **Section B: The Investigators** | | |
| 1. **Chief Investigator** (full name, job title, organisation name & e-mail address for correspondence- see guidance notes for eligibility)   Name: Dr. Duncan Edwards  Post: NIHR Doctoral Research Fellow  Organisation: University of Cambridge  Email: dae31@medschl.cam.ac.uk  CV has been previously submitted to ISAC  **CV number:**  A new CV is being submitted with this protocol  An updated CV is being submitted with this protocol | | |
| 1. **Affiliation** (full address)   Department of Public Health and Primary Care, University of Cambridge, Forvie Site, Robinson Way, Cambridge CB2 0SR | | |
| 1. **Corresponding Applicant**   Name: Dr Steven Kiddle  Job title: MRC Career Development Award fellow  Organisation: MRC Biostatistics Unit, University of Cambridge  Email: steven.kiddle@mrc-bsu.cam.ac.uk  Same as chief investigator  CV has been previously submitted to ISAC  **CV number:** 650_16S  A new CV is being submitted with this protocol  An updated CV is being submitted with this protocol | | |
| 1. **List of all investigators/collaborators** (*please list the full names, affiliations and e-mail addresses* of all collaborators*, *other than the Chief Investigator*)   Other investigator: Professor Simon Griffin, Department of Public Health and Primary Care, University of Cambridge, ProfGP@medschl.cam.ac.uk  CV has been previously submitted to ISAC  **CV number:**  A new CV is being submitted with this protocol  An updated CV is being submitted with this protocol  Other investigator: Dr Kirsty Rhodes, MRC Biostatistics Unit, Kirsty.rhodes@mrc-bsu.cam.ac.uk  CV has been previously submitted to ISAC  **CV number:** 173_15S  A new CV is being submitted with this protocol  An updated CV is being submitted with this protocol  Other investigator: Amelia Harshfield, Department of Public Health and Primary Care, University of Cambridge, ah824@medschl.cam.ac.uk  CV has been previously submitted to ISAC  **CV number:** 422_15  A new CV is being submitted with this protocol  An updated CV is being submitted with this protocol  Other investigator: Dr Rupert Payne, School of Social and Community Medicine, University of Bristol, [rupert.payne@bristol.ac.uk](mailto:rupert.payne@bristol.ac.uk)  CV has been previously submitted to ISAC  **CV number:** 177_15CEP  A new CV is being submitted with this protocol  An updated CV is being submitted with this protocol  Other investigator: Dr Steven Kiddle, MRC Biostatistics Unit, University of Cambridge, [steven.kiddle@mrc-bsu.cam.ac.uk](mailto:steven.kiddle@mrc-bsu.cam.ac.uk)  CV has been previously submitted to ISAC  **CV number:** 650_16S  A new CV is being submitted with this protocol  An updated CV is being submitted with this protocol  Other investigator: Dr Yajing Zhu, MRC Biostatistics Unit, University of Cambridge, [Y.Zhu18@lse.ac.uk](mailto:Y.Zhu18@lse.ac.uk)  CV has been previously submitted to ISAC  **CV number:** 174_18  A new CV is being submitted with this protocol  An updated CV is being submitted with this protocol  Other investigator: Dr Paul Kirk, MRC Biostatistics Unit, University of Cambridge, [paul.kirk@mrc-bsu.cam.ac.uk](mailto:paul.kirk@mrc-bsu.cam.ac.uk)  CV has been previously submitted to ISAC  **CV number:**  A new CV is being submitted with this protocol  An updated CV is being submitted with this protocol  Other investigator: Prof Sylvia Richardson, MRC Biostatistics Unit, University of Cambridge, [sylvia.richardson@mrc-bsu.cam.ac.uk](mailto:sylvia.richardson@mrc-bsu.cam.ac.uk)  CV has been previously submitted to ISAC  **CV number:**  A new CV is being submitted with this protocol  An updated CV is being submitted with this protocol  [Please add more investigators as necessary]**Please note that your ISAC application form and protocol* ***must*** *be copied to all e-mail addresses listed above at the time of submission of your application to the ISAC mailbox. Failure to do so will result in delays in the processing of your application.* | | |
| 1. **Conflict of interest statement*** (please provide a draft of the conflict (or competing) of interest (COI) statement that you intend to include in any publication which might result from this work)   The authors do not have any conflict of interest.  **Please refer to the International Committee of Medical Journal Editors (ICMJE) for guidance on what constitutes a COI* | | |
| 1. **Experience/expertise available** (please complete the following questions to indicate the experience/expertise available within the team of investigators/collaborators actively involved in the proposed research, including the analysis of data and interpretation of results   **Previous GPRD/CPRD Studies** **Publications using GPRD/CPRD data**  None  1-3  > 3 | | |
|  | **Yes** | **No** |
| **Is statistical expertise available within the research team?**  *If yes, please indicate the name(s) of the relevant investigator(s)*   - Kirsty Rhodes, Steven Kiddle, Sylvia Richardson, Paul Kirk and Yajing Zhu are statisticians in the MRC Biostatistics Unit. |  |  |
| **Is experience of handling large data sets (>1 million records) available within the research team?**  *If yes, please indicate the name(s) of the relevant investigator(s)*   - Amelia Harshfield, Steven Kiddle, Yajing Zhu |  |  |
| **Is experience of practising in UK primary care available within the research team?**  *If yes, please indicate the name(s) of the relevant investigator(s)*   - Professor Simon Griffin is Professor of General Practice at the University of Cambridge School of Clinical Medicine. He is also a practicing GP in Cambridge. - Dr. Duncan Edwards is a National Institute of Health and Research Doctoral Research fellow and a practicing GP in South Norfolk. |  |  |
| 1. **References relating to your study**   Please list up to 3 references (most relevant) relating to your proposed study:  1. Barnett, K., Mercer, S. W., Norbury, M., Watt, G., Wyke, S., & Guthrie, B. (2012). Epidemiology of multimorbidity and implications for health care, research, and medical education: a cross-sectional study. *Lancet*, *380*(9836), 37–43.  2. Salisbury, C., Johnson, L., Purdy, S., Valderas, J. M. & Montgomery, A. A. Epidemiology and impact of multimorbidity in primary care: a retrospective cohort study. *Br. J. Gen. Pract.* **61,** e12–21 (2011).  3. van Oostrom, S. H., Picavet, H. S. J., de Bruin, S. R., Stirbu, I., Korevaar, J. C., Schellevis, F. G., & Baan, C. A. (2014). Multimorbidity of chronic diseases and health care utilization in general practice. *BMC Family Practice*, *15*(1), 61. | | |
| **Section C: Access to the data** | | |
| 1. **Financial Sponsor of study**   Pharmaceutical Industry  *Please specify:*      Academia  *Please specify:*  Government / NHS  *Please specify:*      Charity *Please specify:*  Other  *Please specify:*      None | | |
| 1. **Type of Institution carrying out the analyses**   Pharmaceutical Industry *Please specify:*      Academia *Please specify:* University of Cambridge  Government Department *Please specify:*      Research Service Provider *Please specify:*  NHS *Please specify:*      Other  *Please specify:* | | |

| 1. **Data source**   The sponsor has direct access to CPRD GOLD and will extract the relevant data*    A data set will be supplied by CPRD**  CPRD has been commissioned to extract the relevant data and to perform the analyses  Other *Please specify:*    *If data sources other than CPRD GOLD are required, these will be supplied by CPRD  ** Please note that datasets provided by CPRD are limited in size. Applicants should contact CPRD ([KC@CPRD.com](mailto:KC@CPRD.com)) if a dataset of >300,000 patients is required. |
| --- |
| 1. **Primary care data** (please specify which primary care data set(s) are required)   Vision only (Default for CPRD studies)  EMIS^®^ only*  Both Vision and EMIS^®^*  *Note: Vision and EMIS are different clinical systems, Vision data has traditionally been used for CPRD, EMIS is currently undergoing beta-testing.*  **Investigators requiring the use of EMIS data must discuss the study with a member of CPRD staff before submitting an ISAC application*  Please list below the name of the person/s at the CPRD with whom you have discussed your request for EMIS data: |
| **Section D: Data linkage** |
| 1. **Does this protocol also seek access to data held under the CPRD Data Linkage Scheme?**   Yes*  No  If No, please move to section E.  **Investigators requiring linked data must discuss the study with a member of CPRD staff. It is important to be aware that linked data are not available for all patients in CPRD GOLD, the coverage periods for each data source may differ and charges may be applied. Please contact the CPRD Research Team on +44 (20) 3080 6383 or email* [*kc@cprd.com*](mailto:kc@cprd.com) *to discuss your requirements before submitting your application.*  Please list below the name of the person/s at the CPRD with whom you have discussed your request:  Tarita Murray-Thomas  *Please note that as part of the ISAC review of linkages, the protocol may be shared - in confidence - with a representative of the requested linked data set(s) and summary details may be shared - in confidence - with the Confidentiality Advisory Group of the Health Research Authority.* |

| 1. **Please select the source(s) of linked data being requested:**   ONS Mortality Data  NCDR Cancer Registry Data*  Inpatient Hospital Episode Statistics  MINAP  Outpatient Hospital Episode Statistics  Mother Baby Link    Index of Multiple Deprivation  Townsend Score  Other** *Please specify:*  **Please note that applicants seeking access to cancer registry data must provide consent for publication of their study title and study institution on the UK Cancer Registry website. They must also complete a* ***Cancer Dataset Agreement Form*** *(available from CPRD) and provide a* ***System level Security Policy*** *for each organisation involved in the study.*  *** If “Other” is specified, please name an individual in CPRD that this linage has been discussed with.* |
| --- |
| 1. **Total number of linked datasets requested including CPRD GOLD**: 2 |
| 1. **Is linkage to a local dataset with <1 million patients being requested?**   Yes*  No  ** If yes, please provide further details:* |
| 1. **If you have requested linked data sets, please indicate whether the Chief Investigator or any of the collaborators listed in response to question 5 above, have access to any of the linked datasets in a patient identifiable form, or associated with a patient index.**   Yes*  No  ** If yes, please provide further details:* |
| 1. **Does this study involve linking to patient *identifiable* data from other sources?**   Yes  No |
| **Section E: Validation/verification** |
| 1. **Does this protocol describe a purely observational study using CPRD data (this may include the review of anonymised free text)?**   Yes*  No**  ** Yes: If you will be using data obtained from the CPRD Group, this study does not require separate ethics approval from an NHS Research Ethics Committee.*  *** No: You may need to seek separate ethics approval from an NHS Research Ethics Committee for this study. The ISAC will provide advice on whether this may be needed.* |
| 1. **Does this study require anonymised free text?**   Yes*  No  **Please note that work involving free text can only be performed on the July 2013 CPRD GOLD database build or earlier versions. CPRD can provide further advice on the use of anonymised free text.* |
| 1. **Does this protocol involve requesting any additional information from GPs?**   Yes*  No  * *Please indicate what will be required:*  Completion of questionnaires by the GP*^ψ^* Yes  No  Provision of anonymised records (e.g. hospital discharge summaries) Yes  No  Other (please describe)  *^ψ^ Any questionnaire for completion by GPs or other health care professional must be approved by ISAC before circulation for completion.* |
| 1. **Does this study require contact with patients in order for them to complete a questionnaire?**   Yes*  No  **Please note that any questionnaire for completion by patients must be approved by ISAC before circulation for completion.* |
| 1. **Does this study require contact with patients in order to collect a sample?**   Yes*  No  ** Please state what will be collected:* |
| **Section F: Signatures** |
| 1. **Signature from the Chief Investigator**   I confirm that the above information is to the best of my knowledge accurate, and I have read and understood the guidance to applicants.  Name:      Anna Cassell Date:            E. signature (type name): Anna Cassell |

**Protocol Section**

1. **Lay Summary (Max. 200 words)**

*Please provide a succinct overview of your proposed research in plain English i.e. non-technical language. This should cover the background, purpose of the study and the potential importance of the findings. References and abbreviations should be avoided. If you have ticked the "other" box in response to question 4 on the application form, up to an additional 100 words should be used to describe the benefit to public health expected from the study.*

Increasing numbers of people have more than one long term illness, such as diabetes or depression. While healthcare delivery and research has traditionally focused on treatment of individual diseases, the presence of two or more chronic diseases, known as multimorbidity, has become an increasingly common medical issue. Individuals with multimorbidity tend to have more complicated healthcare needs, often leading to increased doctors visits and hospitalisations.

The primary aim of this study is to use routinely collected medical record data to describe the prevalence of multimorbidity in English adults. We will examine the percentage of people in a sample of English patients that have multimorbidity, and the association of individual characteristics including age, sex, and ethnicity with multimorbidity. Additionally, we will assess the association of multimorbidity with usage of health services based on a patient’s number of GP visits, number of prescriptions, and how often they are admitted to hospital.

This work has the potential to benefit patients and health service providers. Through understanding how sociodemographic characteristics impact multimorbidity, we may be able to more appropriately target interventions for multimorbid patients. Additionally, it will allow for better planning and distribution of resources to better care for patients suffering from multimorbidity.

1. **Technical Summary (Max. 200 words)**

*Please provide a succinct overview of the objectives, methods and data analysis for the proposed research. Avoid the use of references in this section*.

Multimorbidity is becoming increasingly prevalent in England, and the effective management of multimorbid patients should be a priority of primary care medicine. In order to effectively plan health services to treat multimorbid patients, we must understand the epidemiology of this condition, and the current relationship between multimorbidity and health service usage.

This research is primarily a descriptive epidemiological study looking at the burden of multimorbidity in primary care in England. It will consist of both a cross-sectional, descriptive study of the prevalence of multimorbidity, and a follow-on retrospective cohort analysis investigating the relationship between multimorbidity and health service utilisation. Using a random of sample of 425,400 patient records, we will describe the prevalence of multimorbidity, and will investigate differences in multimorbidity among various sociodemographic covariate groups. Additionally, we will investigate any linkages between mental and physical comorbidities, and patterns of the most common comorbidities.

In the four-year retrospective cohort aspect of our study, we will use regression models to examine how health service utilisation may differ according to the presence of multimorbidity. Our primary outcomes will be the number of GP consultations, the number of prescriptions dispensed, and the number of hospitalisations.

1. **Objectives, Specific Aims and Rationale**

*Please include:*

*(i) The broad research objectives*

*(ii) The specific aims; any hypotheses to be tested should be stated here.*

*(iii) An explanation of how achievement of the specific aims will further the research objectives*

The main objectives of this research are to describe the distribution and patterns of multimorbidity in adults in England, and to investigate the relationship between multimorbidity and health service utilisation. We are also interested in the ways that particular diseases cluster together, because certain comorbidities have been consistently related to increased disease burden and healthcare utilisation^1^.

The selected primary objective of this study is to investigate the burden of multimorbidity in adults in England, which will be established by calculating the prevalence of multimorbidity with 95% confidence intervals. The secondary objective is to describe primary care health service usage in relation to multimorbidity using the number of GP consultations, the number of medication prescriptions dispensed, and the number of hospitalisations.

To meet these objectives, the specific aims of this study in an English general practice population are:

1. To use a representative, random sample of a large cohort of adult patients (≥18 years old) registered to an English general practice to describe the epidemiology of multimorbidity by sex, age, ethnicity, socioeconomic status, smoking status, and region.
2. To describe the frequency of pairs of two comorbid conditions in order to identify which morbidities most commonly present together.
3. To describe the relationship between the presence of mental health comorbidities and the number of physical morbidities an individual has.
4. To describe the utilisation of health services by individuals with multimorbidity based on the number of yearly GP consultations, the number of annual prescriptions dispensed, and the number of hospital admissions.
5. To determine patterns of multimorbidity disease combinations associated with health service utilisation outcomes, in order to identify clusters of patients that are in some respect “similar.”
6. **Background**

*Please provide a succinct review of the relevant background literature with references so as to explain the purpose of the study. Please ensure that you refer to any previous research in CPRD that is related, providing published references and, when known, the ISAC Protocol Number.*

The current paradigm for medical research tends to focus single diseases. This prioritisation of the study of individual diseases and treatments often excludes patients suffering from multiple chronic illnesses^2^. However, one of the central components of a strong primary care system is caring for the entire patient rather than focusing on a particular body system or disease^3^. As the UK population ages and more patients develop multiple chronic conditions, management of multimorbidity will become an increasingly important aspect of primary care medicine and research.

The current literature suggests that a substantial part of the population has multimorbidity, and while there are some discrepancies in the estimates of prevalence, these differences may be largely attributed to differences in defining morbidities. In Scotland, Barnett, *et al.*’s 2012 study found that 23.3% of all registered patients age 16 and older had two or more morbidities^4^, while Salisbury, *et al.* (2011) found the prevalence of multimorbidity to be 16% in UK adults^5^. This burden of multimorbidity places large demands on GPs, because it has been reported that multimorbid patients account for 78% of GP consultations^6^.

In addition to the demands that multimorbidity places on GPs, multimorbidity is also a serious concern at both the patient and healthcare system level. In individuals, multimorbidity has been associated with higher incidence of acute diseases^7^, increased rates of disability^8^, increased mortality^1^, and decreased quality of life^8^. At the healthcare delivery level, multimorbidity has been associated with greater outpatient and inpatient health service utilization^4,9^, as well as with significantly increased healthcare expenditure on multimorbid patients compared to those with zero or one morbidity^10,11^.

Research on the epidemiology of multimorbidity and its relationship to health service utilisation plays an important role in informing the planning, organization, and delivery of health services to patients with multimorbidity^6^. Salisbury, *et al.*  used GPRD data to conduct research on multimorbidity and health service utilization, and found that people with multimorbidity had higher rates of GP consultations and less continuity of care^6^. Another metric that has been used to assess health service utilisation using CPRD/GPRD data is the number of prescribed drugs^12^. In their 2013 study, Brilleman *et al.* reported that the number of prescribed drugs is the most powerful predictor of future consultations and health service utilisation^12^.

While limited in number, there have been a few studies, in addition to the aforementioned Salisbury *et al.* and Brilleman *et al.* studies, investigating multimorbidity using CPRD/GPRD data^13,14^. One study by Booth *et al.* found a strong association between obesity and multimorbidity^13^. However, they only used 11 chronic conditions in their definition of multimorbidity, which excluded a number of conditions that would reasonably constitute morbidities^13^. Another study by Brilleman *et al.* compared the ability of different measures of multimorbidity to predict future healthcare costs^14^. The results suggested that there can be large differences in the prevalence of multimorbidity, depending on which multimorbidity measure is used^14^. They also found that “the best performing multimorbidity measures were simple counts of the number of chronic conditions patients suffered from”^14^. We will use this “simple count” approach to define the number of comorbidities each patient has.

The Barnett *et al.* paper on multimorbidity in Scotland has provided an important foundation for studying the epidemiology of multimorbidity using a large, primary care dataset^4^. Our study intends to use CPRD data replicate and expand on this work, in order to describe the distribution of multimorbidity in England in replicable and clinically meaningful ways. In their research, Barnett *et al.* examined the association of of sex, age, and socioeconomic status with multimorbidity, and looked at the effect that having a mental-health comorbidity has on the number of physical morbidities^4^. We will build upon this work in the following ways: by using the additional covariates of ethnicity, smoking status, and region; by looking more extensively at the disease patterns and clustering effects of comorbidities; and by examining health service utilisation in relation to multimorbidity.

In the current literature, there is no consensus on how to define multimorbidity^4,15^. A 2013 systematic review of 39 studies of the prevalence, patterns, and determinants of multimorbidity in primary care found that the number of health conditions that these studies defined as multimorbidity ranged form 5 conditions to 335 conditions^15^. To create a list of comorbidities that identify patients with multimorbidity in primary care, the Cambridge Primary Care Unit has drawn on the work of Barnett et al. to develop a list of chronic diseases and long-term illnesses that have face value validity as entities that contribute to multimorbidity in primary care. See Appendix 1 for the list of these morbidities and for the criterion that will be used to define these diseases and conditions. The Barnett et al. multimorbidity list was initially developed based on recommendations obtained from a systematic review of comorbidities relating to cardiovascular disease^16^, from the UK Quality and Outcomes Framework^17^, and from important long term disorders that have been identified by NHS Scotland^4,18^. We have chosen to use the Barnett et al. list as the baseline from which to build our list of morbidities for a number of reasons. First, the selection of comorbidities in this list is based on clinical relevance rather than their usefulness to predict outcomes^4^. We believe this focus on clinical relevance provides the most meaningful description of the epidemiology of multimorbidity in primary care. In contrast, some multimorbidity scores such as the Charlson score aim to predict mortality, and often only include conditions that improve predictability^19,20^. As a result, these existing predictive measures such as the Charlson score are not as clinically meaningful for describing multimorbidity in general practice as. Additionally, the Barnett et al. list consists of comorbidities that are relevant to the Scottish population, which makes them likely to be relevant in the English population

Three morbidities, treated dyspepsia, anxiety, and glaucoma, were present on the Barnett list, but were excluded from this study. See Appendix 2 for the specific rationale of exclusion.

The specific Read code lists for extracting comorbidities are being developed using existing published lists of codes including clinicalcodes.org, CALIBER, NHS Scotland’s Information Services Division, and conditions incentivized under the Quality and Outcomes Framework, and new lists drawn from all possible codes within CPRD. Two clinicians will review each set of codes and, when disagreement is present, additional input from the other two clinicians will be used. Additionally, an estimate of the general population prevalence of the comorbidity will be calculated using the selected codes and a sample of CPRD data, and compared with previous epidemiologic studies. See Appendix 3 for sample code lists for hypertension, asthma, and heart failure.

1. **Study Type**

*Specify whether the study will be primarily descriptive, exploratory, hypothesis testing or a methodological piece of research.*

Primarily a descriptive cross-sectional and retrospective cohort analysis.

1. **Study Design**

*Describe the overall research design (for example, case-control, cohort) and reasons for choosing the proposed study design.*

To meet our primary objective of describing the epidemiology of multimorbidity, we will use a cross-sectional design. We will take a random sample of 425,400 patients registered to English General Practices that have provided “research standard” data continuously from January, 2012 until December, 2015. All adult patients (≥18 years of age) from practices meeting this acceptable data quality criteria and who have been registered for at least 12 months prior to January 1, 2012 will be included to create a random sample of 425,400 patients.

To meet our secondary objective of investigating health service utilisation in multimorbid patients, we will use a retrospective cohort design. We will create a cohort of patients beginning on January 1, 2012 and following these patients over four years until December 31, 2015. The exposed group will be all patients who have multimorbidity as of January 1, 2012, and the unexposed group will be patients who do not have multimorbidity. We will look at the relationship between the exposure of multimorbidity and the outcome of health service utilisation, which will be assessed based on number of GP consultation, number of prescription medications, and number hospital admissions over the four-year follow-up period. This study design will help us account for the effect of differential mortality that may potentially occur between the exposed and unexposed groups. Mortality will be a secondary outcome in our four-year follow-up. Any patients who have transferred out of their practice during the follow-up period will be censored.

1. **Sample Size**

*Please provide an estimate of sample size, and, where possible, a formal power calculation. An estimate of the expected number of patients available in the CPRD database should normally be included.*

For our primary objective of describing the prevalence of multimorbidity, we have calculated a sample size estimate of 425,400 patients.

This sample size calculation was obtained using the following formula for calculating sample size in cross-sectional studies of prevalence^21^.

$$n=\frac{Z^{2}P\left( 1-P \right)}{d^{2}}$$

In this formula, *n* is the sample size and Z is the test statistic corresponding to a 95% confidence level. P is the expected prevalence of multimorbidity. We set P to be 23.2%, which was the prevalence of multimorbidity obtained by the Barnett *et al.* study on a similar population in Scotland^4^. *d* is the precision of the estimate, corresponding to the effect size. We set size *d* to be 0.13%, which was based on the 95% confidence intervals of the prevalence of multimorbidity from the Barnett, *et al* paper^4^*.*

Using the LSOA “Linked Source Data”, we observed that there are currently 95.2% English patients who have patient level IMD data.  We therefore increased our sample size from 405,000 to 425,400 to account patients who will have to be dropped due to missing IMD data.

1. **Data Linkage Required (if applicable)**

*Please provide a synopsis of the purpose(s) for which the each of the linkages requested in section 18 of the application form is required.*

The proposed linkage of GOLD data to the patient-level Index of Multiple Deprivation (IMD) dataset will enable us to obtain an estimate of socioeconomic status, which we consider a key predictor of multimorbidity^4,5,22^. The proposed linkage to the Integrated Hospital Episode Statistics (HES) datasets will allow us to obtain much more complete ethnicity data^23^, which we believe to be an important predictor of multimorbidity that has not been adequately addressed in the current literature^24^. It will also allow us to obtain information about the number of hospital admissions, which is one of our outcomes for measuring health service utilisation.

1. **Study Population**

*Define the source and study population, in terms of persons, place, time period, and listing the criteria which will be used to select the study population from the CPRD, i.e any inclusion or exclusion criteria. Please make clear any restrictions imposed by the use of linked datasets.*

A random sample without replacement of approximately 425,400 patients taken from all adults 18 years or older who were currently registered with a GP on or before December 31, 2015.

1. **Selection of comparison group(s) or controls**

*Describe the criteria for eligibility and the procedure for control selection.*

For the retrospective cohort component of our study, cases are all patients in the dataset who have multimorbidity, defined as having two or more of the morbidities listed in Appendix A. The comparison control group will consist of patients who either have zero or one current morbidities in their medical record.

1. **Exposures, Outcomes and Covariates**

*For exposures and outcomes operational definitions (or procedures for developing them) must be provided, supported by preliminary code lists placed in an Annex. A comprehensive list of covariates should also be provided for any study which is not purely descriptive.*

*Exposures and Outcomes for Primary Research Objective:*

Since this is a descriptive study, we do not have a specific exposure for our primary objective. However, we will consider several covariates in our analysis including age, sex, ethnicity, socioeconomic status (using IMD deciles), smoking status, and region.

The outcome of our descriptive analysis is the prevalence of multimorbidity, and we will define multimorbidity using a list of 36 comorbidities (Appendix 1). See Section D for the rationale for choosing these specific morbidities.

In addition to describing the prevalence of multimorbidity, as part of our descriptive analysis we are interested in the pairing and clustering of different comorbidities. To assess the frequency of the most commonly paired comorbidities, our outcome will be pairs of any two separate morbidities from the code list of 36 comorbidities. Additionally, to evaluate our final descriptive outcome relating to the prevalence of physical-mental health comorbidities, mental health comorbidities will be defined as Depression and Anxiety, Alcohol Problems, Dementia, Learning Disability, and Schizophrenia or Bipolar Disorder. All other morbidities will be defined as physical (See Appendix 1).

*Exposures and Outcomes for Secondary Research Objective:*

Our secondary research objective is to investigate the association between multimorbidity and health service utilisation. The exposure for this four-year retrospective cohort study is multimorbidity, which has been defined above.

We will consider three patient-level health service utilisation outcomes:

- the number of GP practice consultations (in-person or by phone)
- the number of prescriptions a patient has been issued
- the number of hospital admissions.

We will consider mortality as a secondary outcome of the retrospective cohort component of our study.

1. **Data/ Statistical Analysis**

*This section should cover both the analytic methods and also the analyses which are to be performed to meet all the specific aims listed earlier. It is important to ensure that this section is clear and specific about any comparisons which will be made*

To meet our first aim of describing the epidemiology of multimorbidity, we will estimate the prevalence of multimorbidity and explore how this may differ according to patient-level characteristics. We will use a chi-square test to examine differences in presence of multimorbidity (0-1 morbidities versus 2+ morbidities) in relation to sociodemographic characteristics outlined above. We will use a t-test to assess differences in the mean number of morbidities by sex, and one-way ANOVA to test differences in the mean number of morbidities by age (which will be categorised in 10-year age intervals), ethnicity, socioeconomic status (IMD deciles), smoking status, and region.

To meet our second aim of investigating the frequency of pairs of two comorbid conditions, we will describe the prevalence of frequently occurring disease pairs, which we consider to be any pair of comorbidities with a prevalence >5%. These pairs will be identified by calculating the prevalence for all 630 ( $\frac{36*35}{2}$) potential disease couplets, and then reporting the comorbidity pairs with >5% prevalence. As this is a descriptive study, we are primarily interested in identifying the most commonly occurring disease couplets, and describing their prevalence. Clinical appraisal of the most prevalent couplets will be conducted to establish whether these pairs are common because of a causal link, or common because each has relatively high prevalence. These differences will be addressed in our discussion. A chi-squared test will be used to evaluate the association of these co-occurring morbidity pairs (e.g. having the comorbidity pair of interest versus having a different combination of comorbidities) in relation to sociodemographic characteristics. A one-way ANOVA test will be used to assess differences in the mean number of morbidities an individual has given that they have a specific pairing of commonly occurring comorbid diseases.

To meet our third aim of examining mental morbidities and the linkage of physical and mental comorbidities, we will report the percentage of individuals with both a physical and mental comorbidity. We will use a logistic regression, controlling for sociodemographic characteristics, to quantify the association between mental and physical morbidities, with the binary outcome being the presence of a mental health comorbidity. We will report odds ratios and covariate adjusted odds ratios of having any mental health disorder based on the number of physical morbidities an individual has. We will also report the odds ratios by sociodemographic characteristics. Additionally, we will use a chi-squared test to assess differences between the prevalence of any multimorbidity and the prevalence of having a physical-mental health comorbidity between our sociodemographic covariates described in Section K.

To meet our fourth aim of describing health service utilisation, we will use the total number of consultations as the denominator to evaluate the proportion of consultations devoted to patients with different levels of multimorbidity. We will also use the mean and standard deviation of the number of consultations and prescriptions to describe consultation and prescribing rates in relation to the number of morbidities. We will calculate the total number of consultations, prescriptions, and hospitalisations a patient has during our four-year study period, and will explore the patterns of the data to determine the most appropriate form of regression to look at the separate relationships between the number of morbidities and the number of consultations, prescriptions, and hospitalisations. In this analysis, we will adjust for patients who died during the four-year follow-up as well as for patients who have been censored due as a result of transferring out of the practice. We will use a Cox proportional hazards model to describe the differences in mortality rate between exposed multimorbidity patients and non-multimorbid patients. All regression analyses will consist of a crude analysis in addition to an adjusted one incorporating the covariates described in Section K.

To meet our fifth aim of determining patterns of multimorbidity associated with health service utilisation outcomes, we will use a latent class analysis (Hagenaars *et al.* 2002) to classify patients according to their distribution of multimorbidities^25^. Within each class of patients, we will assume a distinct multivariable model of health service utilisation outcome. This approach will assist in the identification of class specific factors associated with health service usage. Ideally, we want to fit models including random effects to allow for variation between practices. Depending on the number of classes obtained, this could potentially be quite a big model with a large number of parameters. If appropriate, we will investigate sub-sampling methods for this data set, choosing a suitable representative sample on which to run the chosen models^26^.

1. **Plan for addressing confounding**

*Purely descriptive studies are exempt from this requirement. All other studies should here provide some discussion of what they are doing in the design and/or analysis to control for confounding.*

We will adjust for confounding using multivariable regressions with random-effects models to account for age, sex, socioeconomic deprivation, ethnicity, smoking status, and region, as well as for clustering by GP practice.

1. **Plan for addressing missing data**

*The potential for missing data should be identified and how it will be addressed discussed here.*

We will only use patients with complete age, gender, and IMD data. To reduce the proportion of patients with missing IMD data, we will only use linked practices. Patterns of missingness in the data set will be described and explored at an early stage, and we will consider using multiple imputation techniques where appropriate^27,28^. Missing data for smoking status will be imputed using the last observation carried forward method.

1. **Limitations of the study design, data sources and analytical methods**

*The general limitations of the databases and observational research are well-known. Specific consideration of the potential impact of such limitations should be provided in the context of the proposed study.*

The key limitations to this protocol are as follows:

1. Because this research uses routinely collected medical record data, it is limited by the quality of data recording. There may be inconsistencies between different practices and physicians relating to how accurately and consistently they record data^29^. Additionally, populations that are not registered in the formal sector might be underrepresented in our sample. These shortcomings of using routinely collected data and the potential for bias against underrepresented populations will be acknowledged in the discussion of our findings.
2. There is no way for us to identify patients that have unrecorded, miscoded, or undiagnosed morbidities. We will be unable to separate patients with morbidities but with missing diagnosis codes from those patients for whom lack of a diagnosis code simply reflects the absence of the condition. This may result in an underestimation of the prevalence of certain conditions and of multimorbidity. This may produce some bias, especially if the miscoding and lack of diagnosis occurs systematically in different populations, for instance for those patients who have poor quality of or poor access to primary healthcare. We will address this potential limitation in the discussion of our findings.
3. There is a possibility of inaccurate coding of GP contact type. This may result in uncertainty in our analyses of the number of GP consultations by either overestimating or underestimating the number of actual GP consultations. We will attempt to address this limitation by conducting a sensitivity analysis and using narrower or broader categories of consultation type to determine frequency of GP consultations as required.
4. Despite the fact that particular combinations of diseases may have a stronger association with disease burden than others, our analysis of multimorbidities is not weighted and therefore does not take into account the severity of disorders^1^. Severity of morbidities is a potential confounder as it is likely to affect the number of consultations with GP practitioners as well as the number of medications they have been prescribed. This will be acknowledged in the discussion of our findings.
5. **Patient or user group involvement (if applicable)**

None

1. **Plans for disseminating and communicating study results, including the presence or absence of any restrictions on the extent and timing of publication**

*ISAC expects most studies that it approves to be published in the scientific literature and considers it an ethical obligation for any study with potential public health implications. In cases where multiple publications are likely to arise, a publication plan should be provided in this section.*

This study is being conducted as part of research for a Masters of Philosophy (MPhil) in Primary Care Research at the University of Cambridge. The Cambridge Department of Public Health and Primary Care has a commitment to disseminating the research that it conducts. Therefore, we aim to publish the main findings of this study in an international peer reviewed primary care journal within two years following the completion of the Master’s thesis.

1. **References**

1. Gijsen, R. *et al.* Causes and consequences of comorbidity. *J. Clin. Epidemiol.* **54,** 661–674 (2001).

2. Mercer, S. W., Smith, S. M., Wyke, S., O’Dowd, T. & Watt, G. C. M. Multimorbidity in primary care: developing the research agenda. *Fam. Pract.* **26,** 79–80 (2009).

3. Rawaf, S., De Maeseneer, J. & Starfield, B. From Alma-Ata to Almaty: a new start for primary health care. *Lancet* **372,** 1365–1367 (2008).

4. Barnett, K. *et al.* Epidemiology of multimorbidity and implications for health care, research, and medical education: a cross-sectional study. *Lancet* **380,** 37–43 (2012).

5. Salisbury, C., Johnson, L., Purdy, S., Valderas, J. M. & Montgomery, A. A. Epidemiology and impact of multimorbidity in primary care: a retrospective cohort study. *Br. J. Gen. Pract.* **61,** e12–21 (2011).

6. Salisbury, C., Johnson, L., Purdy, S., Valderas, J. M. & Montgomery, A. A. Epidemiology and impact of multimorbidity in primary care: a retrospective cohort study. *Br. J. Gen. Pract.* **61,** e12–21 (2011).

7. Foguet-Boreu, Q. *et al.* Impact of multimorbidity: acute morbidity, area of residency and use of health services across the life span in a region of south Europe. *BMC Fam. Pract.* **15,** 55 (2014).

8. Garin, N. *et al.* Impact of multimorbidity on disability and quality of life in the Spanish older population. *PLoS One* **9,** e111498 (2014).

9. Zulman, D. M. *et al.* Multimorbidity and healthcare utilisation among high-cost patients in the US Veterans Affairs Health Care System. *BMJ Open* **5,** e007771 (2015).

10. Bähler, C., Huber, C. A., Brüngger, B. & Reich, O. Multimorbidity, health care utilization and costs in an elderly community-dwelling population: a claims data based observational study. *BMC Health Serv. Res.* **15,** 23 (2015).

11. Kadam, U. T., Uttley, J., Jones, P. W. & Iqbal, Z. Chronic disease multimorbidity transitions across healthcare interfaces and associated costs: a clinical-linkage database study. *BMJ Open* **3,** e003109– (2013).

12. Brilleman, S. L. & Salisbury, C. Comparing measures of multimorbidity to predict outcomes in primary care: a cross sectional study. *Fam. Pract.* **30,** 172–8 (2013).

13. Booth, H. P., Prevost, A. T. & Gulliford, M. C. Impact of body mass index on prevalence of multimorbidity in primary care: cohort study. *Fam. Pract.* **31,** 38–43 (2014).

14. Brilleman, S. L. *et al.* Keep it simple? Predicting primary health care costs with clinical morbidity measures. *J. Health Econ.* **35,** 109–22 (2014).

15. Violan, C. *et al.* Prevalence, determinants and patterns of multimorbidity in primary care: a systematic review of observational studies. *PLoS One* **9,** e102149 (2014).

16. Buck, H. G., Akbar, J. A., Zhang, S. J. & Bettger, J. A. P. Measuring comorbidity in cardiovascular research: a systematic review. *Nurs. Res. Pract.* **2013,** 563246 (2013).

17. Employers, N. 2015/16 General Medical Services (GMS) contract Quality and Outcomes Framework (QOF). (2015). at <http://www.nhsemployers.org/~/media/Employers/Documents/Primary care contracts/QOF/2015 - 16/2015 16 QOF guidance for stakeholders.pdf>

18. Information Services Division NHS National Services Scotland. *Measuring Long-Term Conditions in Scotland Information Services Division*. (2008). at <https://www.isdscotland.org/Health-Topics/Hospital-Care/Diagnoses/2008_08_14_LTC_full_report.pdf>

19. Diederichs, C., Berger, K. & Bartels, D. B. The measurement of multiple chronic diseases--a systematic review on existing multimorbidity indices. *J. Gerontol. A. Biol. Sci. Med. Sci.* **66,** 301–11 (2011).

20. Charlson, M. E., Pompei, P., Ales, K. L. & MacKenzie, C. R. A new method of classifying prognostic comorbidity in longitudinal studies: development and validation. *J. Chronic Dis.* **40,** 373–83 (1987).

21. Pourhoseingholi, M. A., Vahedi, M. & Rahimzadeh, M. Sample size calculation in medical studies. *Gastroenterol. Hepatol. from bed to bench* **6,** 14–7 (2013).

22. van den Akker, M., Buntinx, F., Metsemakers, J. F. M., Roos, S. & Knottnerus, J. A. Multimorbidity in General Practice: Prevalence, Incidence, and Determinants of Co-Occurring Chronic and Recurrent Diseases. *J. Clin. Epidemiol.* **51,** 367–375 (1998).

23. Mathur, R. *et al.* Completeness and usability of ethnicity data in UK-based primary care and hospital databases. *J. Public Health (Oxf).* **36,** 684–92 (2014).

24. Wang, F., Xu, S., Shen, X., Guo, X. & Shen, R. Epidemiology of multimorbidity. *Lancet (London, England)* **380,** 1382–3; author reply 1383–4 (2012).

25. Andersen, R. Jacques A. Hagenaars and Allan L. McCutcheon, Eds. Applied Latent Class Analysis. *Can. J. Sociol.* **28,** 584 (2003).

26. Drovandi, C. C. *et al.* A principled experimental design approach to Big Data analysis. (2015). at <http://eprints.qut.edu.au/87946/8/87946.pdf>

27. Little, R. J. A. & Rubin, D. B. in *Statistical Analysis with Missing Data* 1–23 (John Wiley & Sons, Inc., 2014). doi:10.1002/9781119013563.ch1

28. Welch, C., Bartlett, J. & Petersen, I. Application of multiple imputation using the two-fold fully conditional specification algorithm in longitudinal clinical data. *Stata J.* **14,** 418–431 (2014).

29. Herrett, E. *et al.* Data Resource Profile: Clinical Practice Research Datalink (CPRD). *Int. J. Epidemiol.* dyv098– (2015). doi:10.1093/ije/dyv098

**Appendices**

Appendices should be used for essential supporting information only (e.g. code-lists) and they must be cited within the body of the protocol.

**Appendix 1: List of the 36 disease conditions to be included in multimorbidity count**

| Code | Condition | *Criteria* |
| --- | --- | --- |
|  |  |  |
| HYP | Hypertension | *Read Code ever recorded* |
| DEP | Depression and Anxiety | *Read code recorded in last 12 months OR >=4 anti-depressant prescriptions (excluding low dose tricyclics) in last 12 months OR >=4 anxiolytic/hypnotic prescriptions in the last 12 months* |
| PNC | Painful condition | *>=4 POM analgesics in last 12 months OR >= 4 specified anti-epileptics in the absence of an epilepsy Read code in last 12 months* |
| AST | Asthma (currently treated) | *Read code ever recorded AND any asthma prescription in the last 12 months.* |
| CHD | Coronary heart disease | *Read Code ever recorded* |
| DIB | Diabetes | *Read Code ever recorded* |
| THY | Thyroid disorders | *Read Code ever recorded* |
| RHE | Rheumatoid arthritis, other inflammatory polyarthropathies & systematic connective tissue disorders | *Read Code ever recorded* |
| HEL | Hearing loss | *Read Code ever recorded* |
| COP | COPD | *Read Code ever recorded* |
| IBS | Irritable bowel syndrome | *Read code ever recorded OR ≥ 4 prescription only medicine antispasmodic prescription* |
| CAN | New diagnosis of cancer in last five years | *Read code first recorded in last 5 years* |
| ALC | Alcohol problems | *Read Code ever recorded* |
| OPS | Other psychoactive substance misuse | *Read Code ever recorded* |
| CON | Constipation (Treated) | *≥4 laxative prescriptions in last year* |
| STR | Stroke & transient ischaemic attack | *Read Code ever recorded* |
| CKD | Chronic kidney disease | *Best of the last 2 GFR readings <60* |
| DIV | Diverticular disease of intestine | *Read code ever recorded* |
| ATR | Atrial fibrillation | *Read code ever recorded* |
| PVD | Peripheral vascular disease | *Read code ever recorded* |
| HEF | Heart failure | *Read code ever recorded* |
| PRO | Prostate disorders | *Read code ever recorded* |
| EPI | Epilepsy (currently treated) | *Read code ever recorded AND antiepileptic prescription in last 12 months* |
| DEM | Dementia | *Read code ever recorded* |
| SCZ | Schizophrenia (and related non-organic psychosis) or bipolar disorder | *Read code ever recorded/recorded in last 12 months OR Lithium prescribed in last 168 days* |
| PSO | Psoriasis or eczema | *Read code ever recorded AND ≥ 4 related prescriptions in last 12 months (excluding simple emollients)* |
| IBD | Inflammatory bowel disease | *Read code ever recorded* |
| MIG | Migraine | *≥ 4 prescription-only anti-migraine prescriptions in last year* |
| BLI | Blindness and low vision | *Read code ever recorded* |
| SIN | Chronic sinusitis | *Read code ever recorded* |
| LEA | Learning disability | *Read code ever recorded* |
| ANO | Anorexia or bulimia | *Read code ever recorded* |
| BRO | Bronchiectasis | *Read code ever recorded* |
| PRK | Parkinson’s disease | *Read code ever recorded* |
| MSC | Multiple sclerosis | *Read code ever recorded* |
| CLD | Chronic Liver Disease and Viral Hepatitis | *Read code ever recorded* |

**Appendix 2: Morbidities included in Barnett *et al.,* excluded from this study**

| Condition | Reason for exclusion |
| --- | --- |
| Treated Dyspepsia | No valid set of codes developed due to increasing use of proton pump inhibitors use to prevent gastric side effects of medications |
| Anxiety | Significant overlap with depression, therefore combined with depression to form Depression and Anxiety comorbidity |
| Glaucoma | Significant overlap with visual impairment, thus little added value as a separate comorbidity. |
| Viral Hepatitis | Significant overlap with Liver Disease, therefore combined with Liver Disease to form Chronic Liver Disease and Viral Hepatitis comorbidity |

**Appendix 3: Sample Code lists to be used for selecting comorbidities**

| **Disease** | **Code Type** | **Codes** |
| --- | --- | --- |
| Hypertension | Diagnosis | 204,799,351,15377,1894,4372,83473,10818,3712,7329,31755,59383,73293,57288,25371,51635,34744,16059,31387,31341,42229,18765,7057,69753,102458 |
| Asthma | Diagnosis | 5867,22752,73522,41017,11370,80085,74025,75049,74093,81364,74778,74627,84312,77881,83552,79714,74381,86315,74588,81113,74348,81835,78930,84251,76765,78239,74500,10487,3458,3018,13065,3366,84275,5798,78,1555,7146,2290,1208,15248,7731,14777,5627,27926,6707,45782,5267,3665,29325,58196,18323,45073,25796,185,40823,4442,32727,4892,233,232,8335,12987,16070,4606,21232,18207,93353,39478,47684,74458 |
|  | Medication | 10090,10102,10218,10254,10289,10321,10331,10360,10407,10432,10433,10458,10561,10597,10723,10744,10812,10813,10831,1087,1093,10958,10968,1097,1100,11046,11198,11307,11410,11478,11497,11588,11618,11719,11732,11779,11993,12042,12144,12240,12274,1236,1242,1243,1258,1259,12633,1269,12699,12808,12822,12909,12994,13037,13038,13040,13181,13256,13273,13290,13307,13365,1346,13529,13575,13757,13815,13996,1406,1409,1410,1411,1412,1414,1415,1422,1423,1424,1426,14294,14306,14321,14448,14483,14524,14525,14527,14561,14567,14590,14603,14700,14736,14739,14757,14991,15025,15153,1518,15284,15326,15365,1537,15409,15441,15483,1551,1552,15613,15706,15765,16018,16054,16148,16151,16158,1619,1620,16207,1628,1629,1630,16305,1635,1642,16433,16577,16584,16625,1676,1680,1683,1697,1698,16994,17,17002,1711,17140,17185,1725,1727,1728,1734,1741,17465,17590,17654,17670,17696,17874,17875,1794,1801,18140,18288,18299,18308,18314,1832,1833,1834,18394,18421,18456,18484,18537,1861,18622,1882,18848,1885,18968,18988,19031,19121,19350,19376,19389,19401,1950,1951,1952,1956,1957,1959,1960,1961,1962,1974,1975,20171,2020,20225,20825,20838,2092,21005,21102,21224,2125,2147,2148,21482,2152,2158,2159,2160,21769,218,21859,22080,2224,2229,22313,22430,2282,23269,2335,235,23567,23572,23709,23741,2395,23961,24023,24117,24207,24380,2440,24418,24674,24898,25022,25119,25125,25204,25339,25784,25937,2600,26063,2609,2655,26616,26860,26873,26987,27188,2722,2723,27249,273,27340,27505,2757,2758,27593,27679,27842,27944,28073,282,28241,2850,28508,2851,28577,2862,28640,2869,28761,28881,2892,2893,2911,29267,29273,29325,29475,2951,2978,2992,2994,2995,30118,3018,30204,30210,30212,30229,30230,30238,30240,30596,30649,3075,31,31082,3119,31231,314,3150,3163,31774,31845,3187,31933,32050,32102,3220,3254,32874,3289,32893,3297,3305,3306,33089,33258,33373,33588,3363,3374,33817,33849,3388,34018,34029,34134,34162,34310,34311,34315,34428,3443,34618,34619,34702,34739,34794,34859,34919,34995,35000,35011,35014,3546,35510,35522,35557,3556,35602,35631,3570,35724,35744,3585,35862,3666,3688,3743,37612,37615,3763,37791,3786,3787,38,38097,38120,3838,38416,38419,3927,3947,3989,3993,3994,40177,4055,40655,40709,4100,41269,4131,4132,41412,41549,41691,4171,41832,4222,42497,42511,4268,42910,4306,4365,4413,44713,4497,4499,4514,454,4541,4545,45863,4591,4592,4593,4601,46157,4634,4640,4647,465,4665,4688,4759,4801,4803,4842,4926,4942,510,5143,5161,5170,5172,5185,5223,5261,5308,5309,534,5453,549,5516,5521,5522,555,5551,5558,556,5580,5683,5718,5740,5753,5804,5822,5837,5864,5885,5889,5898,590,5941,5942,5975,5992,6050,6081,6315,6325,638,6462,6512,6522,6526,6569,6616,665,6719,674,6746,6758,6772,6780,6796,6839,6911,6938,696,6988,7013,7017,7133,7140,719,7192,7268,7270,7356,746,7576,7602,7638,7653,7711,7730,7731,7732,7733,7788,7832,7841,7891,7935,7948,7954,7964,7965,7972,8,8056,8057,8111,8215,8267,8333,8339,8433,8470,8498,8522,856,8608,8610,862,863,8635,8653,8676,879,880,8806,881,882,883,895,8955,896,898,907,908,909,9092,910,911,9164,9233,9270,9384,942,947,9477,956,957,9571,9577,958,959,9599,960,9635,964,9651,9681,9711,987,99,9921 |
| Heart Failure | Diagnosis | 101138,101137,106897,12550,104333 |

**Amendment 1**

Text from this amendment, which was placed inside the protocol in blue, has been set to black to indicate that it is not new, but copied here for reference.

From the background section:

*In the current literature, there is no consensus on how to define multimorbidity*^4,15^. *A 2013 systematic review of 39 studies of the prevalence, patterns, and determinants of multimorbidity in primary care found that the number of health conditions that these studies defined as multimorbidity ranged form 5 conditions to 335 conditions*^15^*. To create a list of comorbidities that identify patients with multimorbidity in primary care, the Cambridge Primary Care Unit has drawn on the work of Barnett et al. to develop a list of chronic diseases and long-term illnesses that have face value validity as entities that contribute to multimorbidity in primary care. See Appendix 1 for the list of these morbidities and for the criterion that will be used to define these diseases and conditions. The Barnett et al. multimorbidity list was initially developed based on recommendations obtained from a systematic review of comorbidities relating to cardiovascular disease*^16^*, from the UK Quality and Outcomes Framework*^17^*, and from important long term disorders that have been identified by NHS Scotland*^4,18^*. We have chosen to use the Barnett et al. list as the baseline from which to build our list of morbidities for a number of reasons. First, the selection of comorbidities in this list is based on clinical relevance rather than their usefulness to predict outcomes*^4^*. We believe this focus on clinical relevance provides the most meaningful description of the epidemiology of multimorbidity in primary care. In contrast, some multimorbidity scores such as the Charlson score aim to predict mortality, and often only include conditions that improve predictability*^19,20^*. As a result, these existing predictive measures such as the Charlson score are not as clinically meaningful for describing multimorbidity in general practice as. Additionally, the Barnett et al. list consists of comorbidities that are relevant to the Scottish population, which makes them likely to be relevant in the English population*

*Three morbidities, treated dyspepsia, anxiety, and glaucoma, were present on the Barnett list, but were excluded from this study. See Appendix 2 for the specific rationale of exclusion.*

*The specific Read code lists for extracting comorbidities are being developed using existing published lists of codes including clinicalcodes.org, CALIBER, NHS Scotland’s Information Services Division, and conditions incentivized under the Quality and Outcomes Framework, and new lists drawn from all possible codes within CPRD. Two clinicians will review each set of codes and, when disagreement is present, additional input from the other two clinicians will be used. Additionally, an estimate of the general population prevalence of the comorbidity will be calculated using the selected codes and a sample of CPRD data, and compared with previous epidemiologic studies. See Appendix 3 for sample code lists for hypertension, asthma, and heart failure.*

From study design:

*We will take a random sample of 425,400 patients registered to English General Practices that have provided “research standard” data continuously from January, 2012 until December, 2015. All adult patients (≥18 years of age)* *from practices meeting this acceptable data quality criteria and who have been registered for at least 12 months prior to* *January 1, 2012* will be included to create a random sample of 425,400 patients.

*To meet our secondary objective of investigating health service utilisation in multimorbid patients, we will use a retrospective cohort design. We will create a cohort of patients beginning on January 1, 2012 and following these patients over four years until December 31, 2015. The exposed group will be all patients who have multimorbidity as of January 1, 2012, and the unexposed group will be patients who do not have multimorbidity. We will look at the relationship between the exposure of multimorbidity and the outcome of health service utilisation, which will be assessed based on number of GP consultation, number of prescription medications, and number hospital admissions over the four-year follow-up period. This study design will help us account for the effect of differential mortality that may potentially occur between the exposed and unexposed groups. Mortality will be a secondary outcome in our four-year follow-up. Any patients who have transferred out of their practice during the follow-up period will be censored.*

From exposures and outcomes:

*See Section D for the rationale for choosing these specific morbidities.*

*We will consider mortality as a secondary outcome of the retrospective cohort component of our study.*

From Data/Statistical analysis:

*These pairs will be identified by calculating the prevalence for all 630 (* $\frac{36*35}{2}$*) potential disease couplets, and then reporting the comorbidity pairs with >5% prevalence. As this is a descriptive study, we are primarily interested in identifying the most commonly occurring disease couplets, and describing their prevalence. Clinical appraisal of the most prevalent couplets will be conducted to establish whether these pairs are common because of a causal link, or common because each has relatively high prevalence. These differences will be addressed in our discussion.*

*In this analysis, we will adjust for patients who died during the four-year follow-up as well as for patients who have been censored due as a result of transferring out of the practice. We will use a Cox proportional hazards model to describe the differences in mortality rate between exposed multimorbidity patients and non-multimorbid patients.*

H. Data Linkage Required (if applicable)

*Please provide a synopsis of the purpose(s) for which the each of the linkages requested in section 18 of the application form is required.*

The proposed linkage of GOLD data to the patient-level Index of Multiple Deprivation (IMD) dataset will enable us to obtain an estimate of socioeconomic status, which we consider a key predictor of multimorbidity^4,5,22^. The proposed linkage to the Integrated Hospital Episode Statistics (HES) datasets will allow us to obtain much more complete ethnicity data^23^, which we believe to be an important predictor of multimorbidity that has not been adequately addressed in the current literature^24^. It will also allow us to obtain information about the number of hospital admissions, which is one of our outcomes for measuring health service utilisation.

**Amendment 2**

The following amendment is proposed, as of 24/09/2018:

Investigators

The research team would like to add statisticians Dr Steven Kiddle, Prof Sylvia Richardson, Dr Paul Kirk and Dr Yajing Zhu to the project.

Objectives

We wish to update Objective 5:

1. To determine patterns of multimorbidity disease combinations associated with health service utilisation outcomes, treatment burden, mortality, index of multiple deprivation and age in order to identify clusters of patients that are in some respect “similar.”

Data Linkage Required (if applicable)

To assess the relationship between multimorbidity clusters and mortality, we additionally wish to link ONS mortality data.

Data/Statistical Analysis

We propose to use profile regression (see below) instead of latent class analysis to cluster patients based on their health conditions. Formally, profile regression is a latent class analysis, with the addition that clusters are sought which are related to the outcome. As described above, we will generate clusters associated with health service usage, treatment burden (number of current prescription medications), mortality, index of multiple deprivation and age. This will ensure the clusters found are relevant to those outcomes, and so will be most useful. Rather than random effects for practices, we will study the impact of practice-level variation by adding it as a fixed effect co-variate into our model. We will perform cross-validation to ensure the robustness of our results.

**Reference for profile regression:** https://arxiv.org/abs/1303.2836

**Amendment 3**

The following amendment is proposed, as of 27/11/2019:

Data/Statistical Analysis

For the first publication on clustering of multimorbidity patients we used Latent Class Analysis, as specified in the original protocol. This will be applied in patients stratified by age (18 – 44, 45 – 64, 65 – 84, 85+ years old) to highlight the relevance of different conditions to multimorbidity at different age groups.

Outcome-guided clustering will instead form part of a second clustering paper, that is more methodological. This paper will be about the balance between inference and prediction in studies where we are interested both in clustering and in outcomes. We have already compared various methods on simulated data, and now would like to demonstrate their effect on real data.

There are three quantities of interest: patient cluster (C), cluster-specific outcome (Y|C where Y=outcome, say mortality), patient-specific outcome (Y|U or Y where U = a vector of conditions a patient has). Ideally we want estimates for all quantities to be accurate, but essentially this leads to the dream of getting both inference (correct C, correct Y|C) and prediction (Y) right; i.e. we aim to uncover the true data-generating mechanism such that prediction is naturally accurate. The problem here is that by introducing cluster (C), we are likely to introduce extra uncertainty in the model such that we may deviate more from a prediction goal.

The methods we will compare are:

- The most popular approach, and the one we have taken so far, called the modal-class approach, where clusters are learned first (based on the vector of conditions a patient has U) and compared to outcomes post-hoc.
- The 1-step approach, with simultaneous estimation of clusters and the relationship with outcomes, assuming conditional independence between indicators (Us) and outcome (Y).
- 1-step local dependence-corrected approach, which takes local dependence into account using a restricted/non-restricted latent factor structure
- Bias-corrected 3-step approach: Recognise misclassification error in the modal-class approach and introduce “misclassification weights” to correct for biased Y|C relationship.
- Regression: Us are included as predictors of Y.

**Amendment 4**

The following amendment is proposed, as of 5/12/2019:

Our application of latent class analysis in age strata to determine multimorbidity clusters has revealed many clusters which we wish to understand better.

Data/Statistical Analysis

Subsequent to the identification of clusters of multimorbid patients using latent class analysis and a list of ~40 conditions, we wish to further characterise these clusters in the following ways:

1. Describing **the prevalence of 308 fine-grained conditions** within these clusters (e.g. prevalence of lung cancer, not just cancer). This will be a descriptive analysis using the 308 conditions defined here: <https://www.caliberresearch.org/portal/phenotypes/chronological-map>
2. **Which conditions are recorded first?** Our existing clusters are based on the prevalence of conditions in 2012, defined in the following way: <https://www.phpc.cam.ac.uk/pcu/cprd_cam/codelists/v11/> . We will begin with a simple descriptive analysis of which diseases within a cluster are (on average) recorded first. After this we may try and calculate for each cluster pairs of conditions (A and B) where A more often comes after B, and the relative risk of B given A happening earlier using the approach of Jensen et al., (2014) Nat Comm.
3. **Which causes of death are associated with each cluster?** We will use perform a descriptive analysis of primary cause of death for each cluster
4. **Can we improve identification of multimorbid individuals through the use of linked HES data?** While we have used HES linkage to look at hospitalisation, we have not used it to identify the diseases an individual has. This made sense from the point of view of studying the GP perspective on multimorbidity, but there will be some patients who receive diagnoses in secondary care that do not get entered into GP records. We will use existing HES definitions from <https://www.caliberresearch.org/portal/phenotypes> . We will assess the prevalence of each of the broad disease areas (from the original protocol) and whether it increases with the addition of HES-only diagnoses. We may also repeat the Latent Class Analysis to see how/if results change.
5. **Do we see similar patterns of multimorbidity in non-HES linked practices?** The non-HES linked practices, often from Scotland, Wales or Northern Ireland, are expected to have similar multimorbidity clusters to England, but there may be some differences. We will re-run our Latent Class Analysis in the non-HES linked practices to assess this.
