# Supplementary material for: Characteristics, service use and mortality of clusters of multimorbid patients in England: a population-based study
Source: BMC Med. 2020 Apr 10;18:78. doi: 10.1186/s12916-020-01543-8 (PMC7147068; doi:10.1186/s12916-020-01543-8)
Supplement: Supplementary file 2 — Additional file 2. Supplementary [file 12916_2020_1543_MOESM2_ESM.docx]

**Supplementary for the paper “Characteristics, service use and mortality of clusters of multimorbid patients in England: a population-based study”**

**Correspondence to:** Yajing Zhu, [yajing.zhu@mrc-bsu.cam.ac.uk](mailto:steven.kiddle@mrc-bsu.cam.ac.uk) ,MRC Biostatistics Unit, University of Cambridge, Cambridge, CB2 0SR, UK.

# Introduction

This supplementary accompanies the main paper and provides more details on the statistical analyses conducted for interested readers.

# Prevalence of conditions and multimorbidity patterns

**Table 1 Full list of morbidities (38 long-term conditions) and their prevalence in the population (N=391,669).** Based on CPRD@Cambridge codelists Version 1·1.

| Order | Morbidity in shorthand | Morbidity in full | Prevalence in population (%) |
| --- | --- | --- | --- |
| 1 | HYP | Hypertension | 18·33 |
| 2 | PNC | Painful conditions | 10·85 |
| 3 | HL | Hearing loss | 9·82 |
| 4 | DEP | Depression | 9·22 |
| 5 | IBS | Irritable bowel syndrome | 6·89 |
| 6 | AST | Asthma | 6·31 |
| 7 | DIA | Diabetes | 5·93 |
| 8 | CHD | Coronary heart disease | 4·87 |
| 9 | THY | Thyroid disorders | 4·76 |
| 10 | ANX | Anxiety | 3·03 |
| 11 | DIV | Diverticular disease of intestine | 2·89 |
| 12 | PSD | Prostate disorders | 2·86 |
| 13 | CKD | Chronic kidney disease | 2·78 |
| 14 | SIN | Chronic sinusitis | 2·49 |
| 15 | STR | Stroke & transient ischaemic attack | 2·45 |
| 16 | COPD | COPD | 2·29 |
| 17 | ATR | Atrial fibrillation | 2·28 |
| 18 | RHE | Rheumatoid arthritis | 2·26 |
| 19 | CSP | Constipation | 2·25 |
| 20 | CAN | Cancer | 1·86 |
| 21 | PUD | Peptic ulcer disease | 1·68 |
| 22 | ALC | Alcohol problems | 1·36 |
| 23 | PSM | Psychoactive substance misuse (NOT ALCOHOL) | 1·18 |
| 24 | BLI | Blindness and low vision | 1·04 |
| 25 | PSO | Psoriasis or eczema | 1·00 |
| 26 | HF | Heart failure | 0·98 |
| 27 | SCZ | Schizophrenia | 0·97 |
| 28 | IBD | Inflammatory bowel disease | 0·87 |
| 29 | PVD | Peripheral vascular disease | 0·87 |
| 30 | DEM | Dementia | 0·74 |
| 31 | AB | Anorexia bulimia | 0·53 |
| 32 | CLD | Chronic liver disease | 0·50 |
| 33 | EPI | Epilepsy | 0·45 |
| 34 | MIG | Migraine | 0·39 |
| 35 | LD | Learning disability | 0·39 |
| 36 | BRO | Bronchiectasis | 0·37 |
| 37 | MS | Multiple sclerosis | 0·27 |
| 38 | PRK | Parkinson's disease | 0·25 |

**Table 2 Top 20 most common unique combinations of conditions (all types of combinations, not limited to pairs) and their prevalence in the whole population (N=391,669).**

| **Morbidities** | **Prevalence (%)** |
| --- | --- |
| Diabetes, hypertension | 0·72 |
| Hypertension, painful conditions | 0·50 |
| Hearing loss, hypertension | 0·48 |
| Anxiety, depression | 0·32 |
| Depression, painful conditions | 0·31 |
| Coronary heart disease, hypertension | 0·29 |
| Hypertension, thyroid disorders | 0·27 |
| Depression, irritable bowel syndrome | 0·27 |
| Hypertension, asthma | 0·21 |
| Hypertension, depression | 0·21 |
| Hearing loss, irritable bowel syndrome | 0·21 |
| Chronic kidney disease, hypertension | 0·21 |
| Hypertension, irritable bowel syndrome | 0·21 |
| Hypertension, prostate disorder | 0·17 |
| Asthma, irritable bowel syndrome | 0·16 |
| Hearing loss, painful conditions | 0·16 |
| Asthma, depression | 0·16 |
| Hearing loss, asthma | 0·16 |
| Hearing loss, depression | 0·14 |
| Hypertension, stroke | 0·14 |

**Table 3 Details on the number of unique combinations of conditions in multimorbid patients in the training and test sets.**

| **Total # morbidity patterns** | | |
| --- | --- | --- |
| Age | Training | Test |
| 18-44 | 2128 | 870 |
| 45-64 | 6613 | 2570 |
| 65-84 | 13422 | 4605 |
| 85+ | 5919 | 1817 |
| Total | 21997 | 7899 |
| **Total # patients** | | |
| Age | Training | Test |
| 18-44 | 12245 | 3061 |
| 45-64 | 28878 | 7219 |
| 65-84 | 39596 | 9898 |
| 85+ | 9852 | 2462 |
| Total | 90571 | 22640 |

# Latent class analysis

## Model selection methodology

Methods to analyse the associations among diseases have included basic analytic approaches such as summarising multimorbidities by counting co-existing chronic diseases^1–3^, weighted or composite score for associated morbidities^4,5^ and k-means and/or hierarchical clustering methods based on dissimilarity measures^6,7^. More advanced statistical approaches, such as the probabilistic model-based latent class analysis (LCA) has been applied to identify clustered multimorbid patterns ^8–10^ but clustering results were highly diverse due to varied sample size, difference in the population of interest (mostly in older patients), approach for model selection (often relying on only one or two information criteria) and the lack of validation of derived cluster solutions.

In this paper, identification of multimorbidity clusters was achieved using the latent class analysis (LCA), where patients with similar patterns of morbidities were grouped into latent classes. Guided by implications from simulation studies^11^, the optimal number of latent classes needed to captured such heterogeneity was decided using a combination of statistics (Bayesian Information Criteria (BIC), sample-sized adjusted BIC (aBIC), difference in log-likelihood from consecutive models (likelihood ratio), entropy^[[1]](#footnote-1)^ for classification quality and clinical judgment. An ideal best-model is the one that is more parsimonious (low BIC and aBIC, low likelihood ratio), with good classification quality (high entropy), captures the most heterogeneity between morbidities and is clinically meaningful. Based on coefficients estimated in the selected model, patients were then assigned to the cluster with the highest estimated posterior probability of membership (i.e. most likely latent class membership). Compared to other clustering methods (e.g. exploratory factor analysis, hierarchical clustering method) which have been typically applied to diseases not patients, there are clear advantages of LCA applied at the patient level. First, it is a model-based clustering approach and hence is not sensitive to the subjective choice of “distance measures” for morbidity patterns and the rotation of factors. This enhances the reproducibility and stability of the latent class solutions. Second, it has two main outputs: the number of clusters of multimorbid patients and the morbidity profile in each cluster. This improves the clusters interpretability.

## Model selection statistics

We present below the model selection statistics for performing LCA with different number of classes for patients in each age strata.

Age strata 18-44


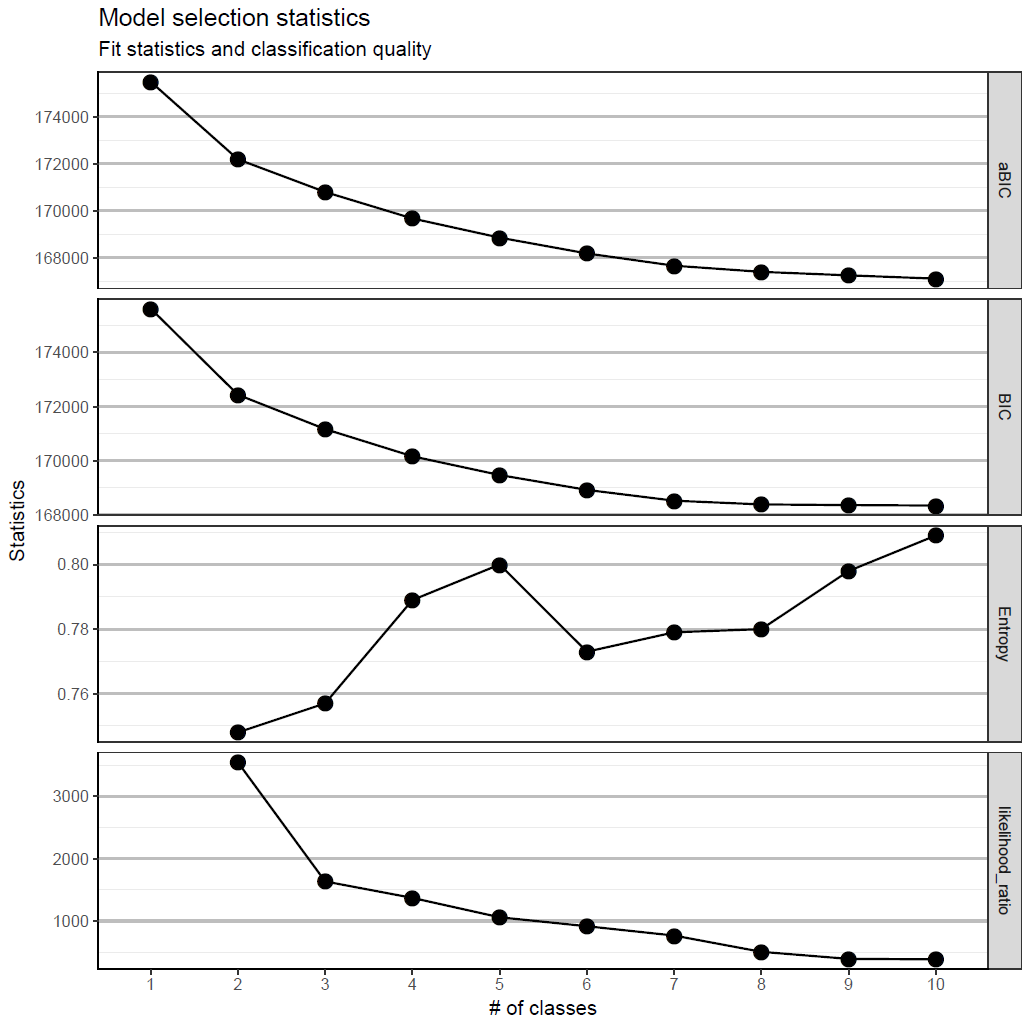


**Figure 1 Model selection statistics for latent class solution for patients aged 18-44 years.**

Best model: 5-class model.

Rationale: This solution has relatively low aBIC and BIC. Another candidate for the best-model is the 9-class solution. However, having extra classes provides little gain in class separation (entropy is not much better than that of a 5-class model) and loses clarity of clinical interpretation.

Age strata 45-64


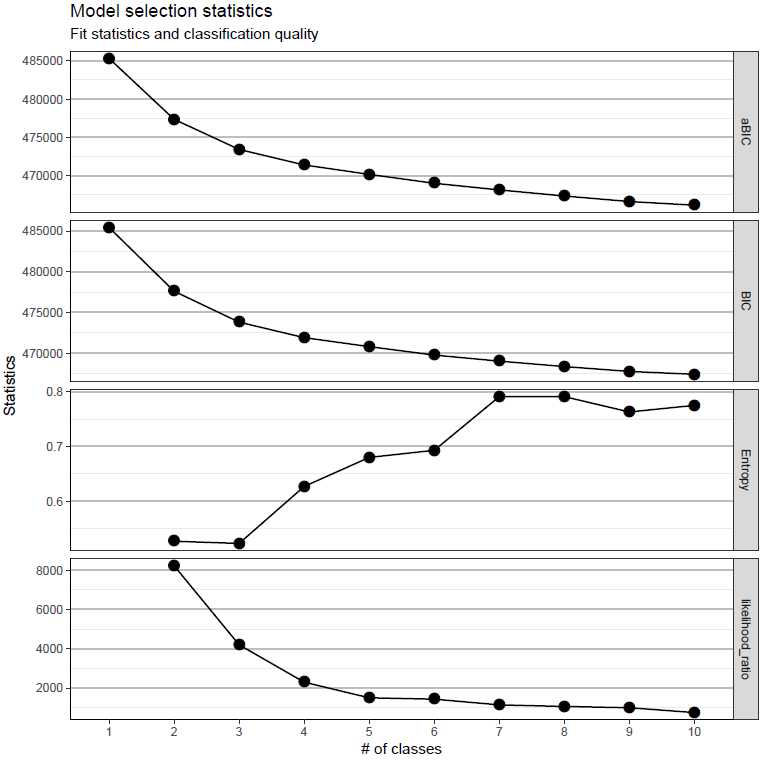


**Figure 2 Model selection statistics for latent class solution for patients aged 45-64 years.**

Best model: 5-class model.

Rationale: Performance of the 6-class model is very similar to 5-class model but gives little gain in selection statistics. Models with more than 6 classes fail to replicate the best likelihood using multiple sets of starting values (8000 sets) so results are likely to be local and unstable; also 7-class model gives limited improvement on likelihood. The higher entropy value for models with more than 6 classes are mostly due to the extraction of pseudo classes (an example of over-fitting) that are induced by local solutions.

Age strata 65-84


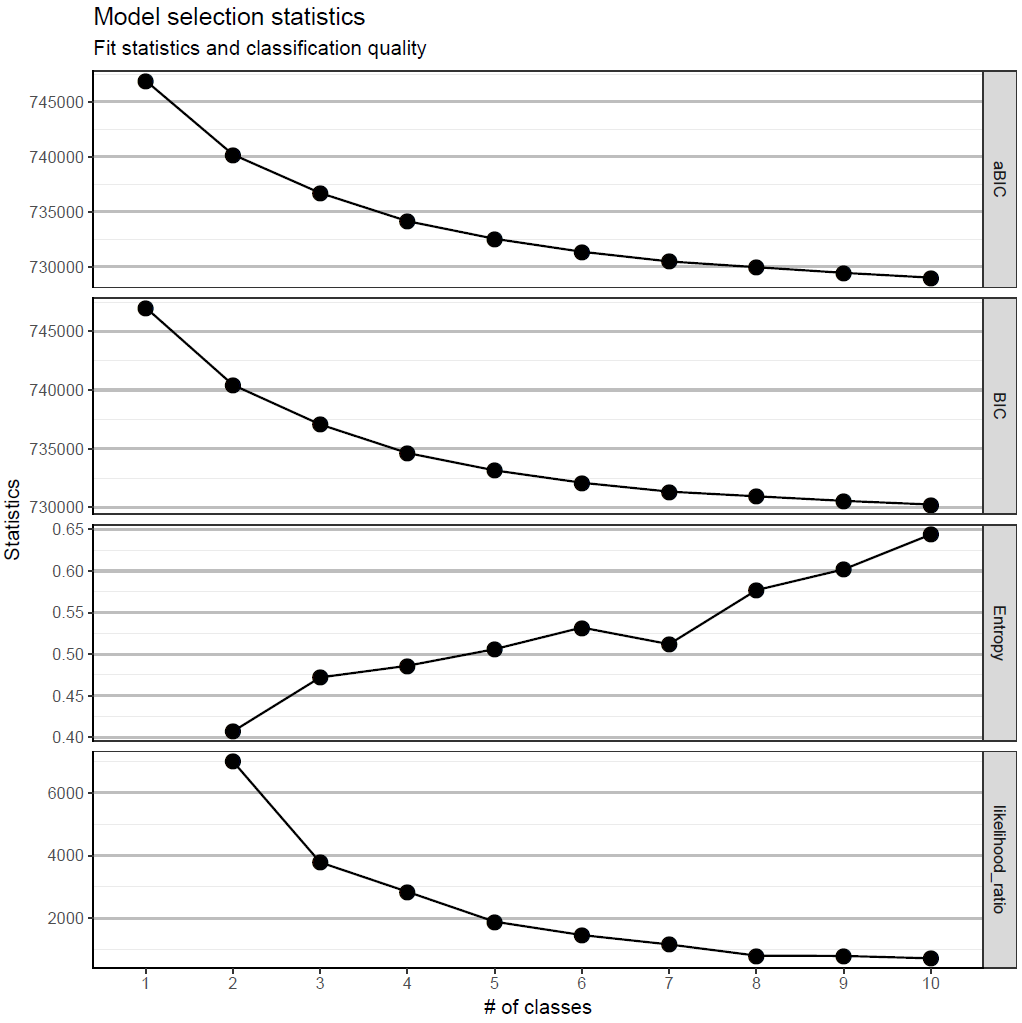


**Figure 3 Model selection statistics for latent class solution for patients aged 65-84 years.**

Best model: 6-class model.

Rationale: Little improvement from models with more than 6 classes in entropy and likelihood ratio. In particular, 10-class model only gives local maxima in likelihood hence is not reliable. 8-class could be another candidate for the best-model but the additional two class profiles are found to be similar to the existing class profiles in the 6-class model.

Age strata 85+


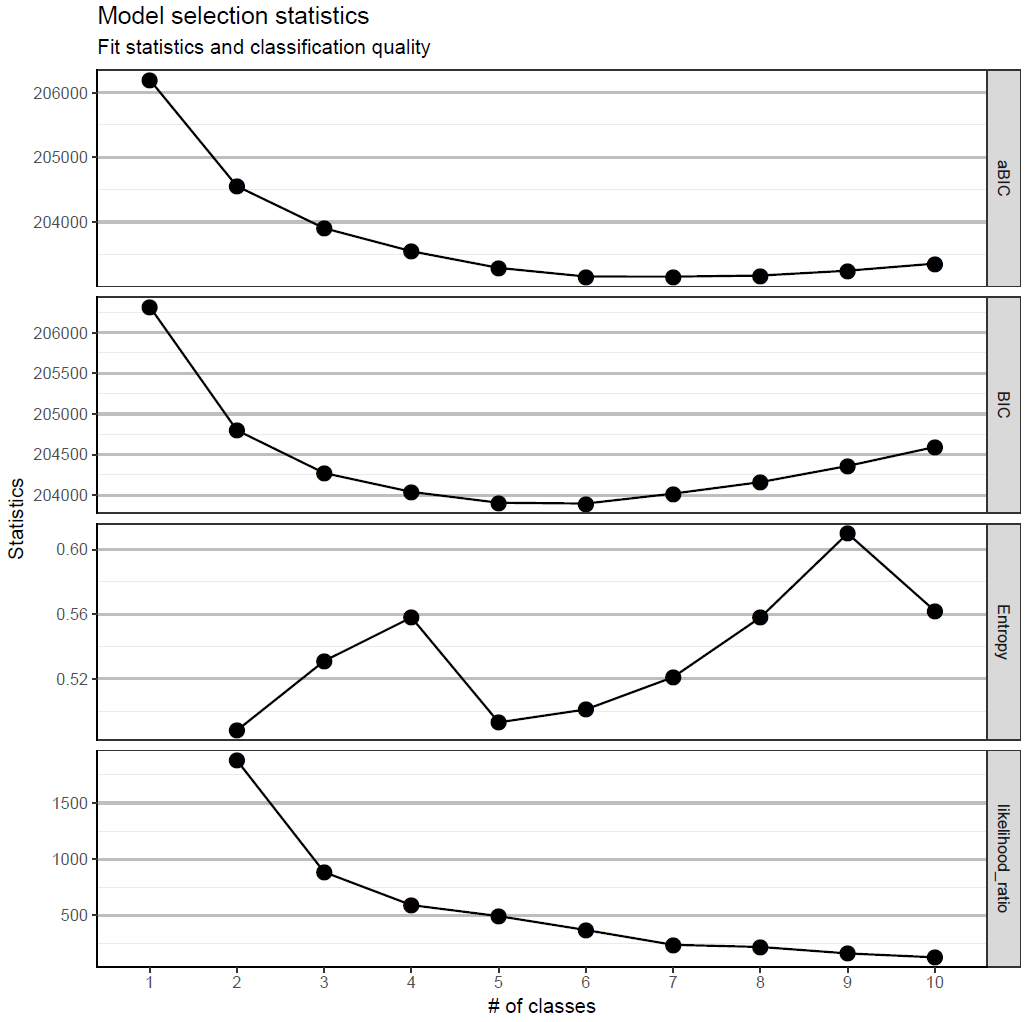


**Figure 4 Model selection statistics for latent class solution for patients aged 85+ years.**

Best model: 4-class model

Rationale: It is clear that the best solution lies among 4-6-class models. Entropy is the highest for the 4-class model and likelihood changes very little when extra classes are added.

# Validation of the latent class solution

Despite some similarities in the commonly identified multimorbidity clusters (“cardiovascular-metabolic disease”, “mental-health-related disease”), most findings from previous research have exhibited heterogeneity due to differences in sample size, data source and the number of diseases considered^12^. To ensure the stability and robustness of our clusters, we employed three methods (check the relationship between clusters and patient demographics and outcomes; check the consistency of the quality of cluster solutions in train and test (using entropy); check the consistency between cluster profiles in the training and test sets (using Jensen-Shannon distance measure^13^ and Pearson’s correlation coefficient^14^) to indirectly validate latent class solutions. Note that a direct approach is impossible as latent classes are unobserved.

We derive the cluster solution in a randomly selected subset of the multimorbid population that contains 80% of the patients, stratified by age group (i.e. training set). To assess the validity and reliability of morbidity cluster, age-stratified LCA was repeated in the held-out 20% of the population (i.e. test set), fixing the number of clusters (i.e. classes) to match that learned from the training set.

First, to check the consistency between cluster profiles in the training and test sets, our aim is to find each cluster in the test set a match in the training set. Given that the training set contains more disease patterns than the test set, clusters in the training set provide a more comprehensive picture of the disease heterogeneity. We therefore expect that clusters in test set are subsets of the clusters identified in the training set. To check this, we match clusters using two criteria: Jensen-Shannon distance and Pearson’s correlation coefficient. The Jensen-Shannon distance (JSD, bounded in 0 to 1, with high values indicating a higher degree of divergence)^13^ between cluster profiles is defined by the square root of Jensen-Shannon divergence measure, based on the Kullback Leibler divergence. JSD has been commonly used to assess the similarity between probability distributions. Note that in our application, before computation, cluster profiles for each most likely class assignment are transformed by dividing the conditional probabilities by their summation (i.e. scaled to the 0-1 range to give a weighted distribution). For each test cluster, the matching cluster in the training set is selected such that the JSD is the smallest (Table 3a). The second criteria, the bi-variate Pearson’s correlation coefficient (bounded in -1 to 1) is computed using the conditional probabilities for 38 LTCs for each pairs of clusters in the training and test set and it assesses the degree of the linear association between two disease profiles. High values (close to 1) indicate a close association between cluster profiles (Table 3b). Table 3a and 3b show that each cluster in the test set has a satisfactory mapping to a cluster in the training set and that there is a clear agreement between results based on two criteria. However, we note that [18-44 age strata: AST,HL,PNC] and [85+ age strata: PNC,DEP,CSP] in the test set seems to be matched with two clusters in the training set (light-grey shaded cells). This is because both matching statistics use the whole profile (38 LTCs) and that test set contains fewer disease patterns than the training set. Most distinctive features (illustrated by cluster labels) can now be consulted and this suggests the dark-grey-shaded cells are the best match. Second, classification quality (measured by entropy) in the training and test are compared (Table 4). Finally, we assess the association between multimorbidity clusters and patient demographics and outcomes. Stability is established if such associations are similar in training and test sets (Tables 5 and 6).

In Table 4, note that morbidity patterns in the training and test sets may be different because only 20% of the multimorbid patients are included in the test set. As a result, relative class size (order of classes) may differ between training and test sets. If class solutions are valid, we will observe similar entropy (classification quality) and similar class profiles in the training and test set, where we list the top 3 most distinctive morbidities in each cluster (Tables 3a, Table 3b and Table 4). Small variation among the prevalence of morbidities is tolerated as the class assignment is based on the most likely class membership, which carries more variability when sample size is small (as in the test set, with 20% of the multimorbid patients).

We are reasonably confident about our results because 1) a large sample (N is above 100,000) is used and is more inclusive of various disease patterns, 2) classification qualities in the training and test sets are similar (entropy value), 3) cluster profiles in the training and test sets are highly consistent (assessed using two criteria) and each cluster in the test set can be well-matched with one cluster in the training set and 4) relationships between clusters and patient demographics and outcomes in the training and test sets are highly consistent. In general, because clusters are unknown by nature, there is a lack of consensus in the statistical literature for the validation of latent class solutions (particularly for large datasets with a large number of patterns). Our validation procedures are indirect but they offer a consistent solution. Validation can be further improved when more methodological work comes to light.

For example, as similarity measures for cluster profiles do not have a theoretical cut-off point, statistical tests may further strengthen our confidence in the matched clusters but we do not consider them in this context due to the following reasons. First, sample size for such a comparison is 38 (LTCs) and is not large enough for sufficient statistical power. Second, morbidities in each cluster should not be given equal weight as cluster profiles are essentially defined by the most distinctive morbidities. Some weighting strategy is needed but this requires a more detailed methodological discussion which is beyond the scope of this paper. Finally, cluster assignments are based on most likely class membership but classification error is present. Similarity tests for clusters in the training and test data need to account for misclassification error when clusters are not observed and again, more methodological research is required.

**Table 4a Jensen-Shannon distance (JSD, range = [0-1], high values indicate a higher degree of divergence) for the similarities between corresponding clusters profiles (for 38 LTCs) in the training (rows) and test sets (columns).** Each cluster in the test set is matched with a cluster in the training set with the smallest JSD (with zero indicating perfect similarity). Matched clusters are shaded in dark grey (secondary choices ae shaded in light grey).

| **18-44** | **DEP,ANX,PNC** | **AST,HL,PNC** | **IBS,DEP,AST** | **HYP,DIA,DEP** | **PSM,ALC,PNC** |  |
| --- | --- | --- | --- | --- | --- | --- |
| DEP,ANX,PNC | 0·05 | 0·42 | 0·42 | 0·53 | 0·46 |  |
| PNC,HL,HYP | 0·44 | 0·29 | 0·46 | 0·32 | 0·52 |  |
| AST,IBS,DEP | 0·45 | 0·32 | 0·34 | 0·48 | 0·53 |  |
| IBS,DEP,HL | 0·46 | 0·47 | 0·19 | 0·57 | 0·59 |  |
| PSM,ALC,DEP | 0·43 | 0·48 | 0·53 | 0·58 | 0·12 |  |
| **45-64** | **HYP,DIA,CHD** | **IBS,HL,AST** | **DEP,PNC,ANX** | **PNC,CHD,DIA** | **ALC,PSM,PNC** |  |
| HYP,DIA,PNC | 0·13 | 0·38 | 0·43 | 0·21 | 0·48 |  |
| IBS,HL,PNC | 0·42 | 0·17 | 0·36 | 0·37 | 0·49 |  |
| DEP,PNC,ANX | 0·46 | 0·35 | 0·05 | 0·36 | 0·36 |  |
| AST,COPD,PNC | 0·42 | 0·27 | 0·42 | 0·40 | 0·50 |  |
| ALC,PSM,PNC | 0·50 | 0·46 | 0·41 | 0·44 | 0·13 |  |
| **65-84** | **HYP,DIA,CKD** | **HL,IBS,DIV** | **CHD,AF,DIA** | **DEP,PNC,ANX** | **AST,COPD,PNC** | **PNC,DEP,CHD** |
| HYP,DIA,CKD | 0·03 | 0·30 | 0·26 | 0·35 | 0·35 | 0·27 |
| HL,PSD,IBS | 0·28 | 0·07 | 0·33 | 0·32 | 0·33 | 0·28 |
| DEP,PNC,ANX | 0·34 | 0·32 | 0·42 | 0·07 | 0·39 | 0·21 |
| CHD,AF,DIA | 0·26 | 0·36 | 0·05 | 0·41 | 0·40 | 0·26 |
| COPD,AST,PNC | 0·36 | 0·33 | 0·36 | 0·34 | 0·11 | 0·29 |
| PNC,CHD,DEP | 0·28 | 0·29 | 0·25 | 0·23 | 0·31 | 0·06 |
| **85+** | **HYP,CHD,HL** | **DEP,PNC,CSP** | **PNC,DEP,CSP** | **COPD,AST,PSD** |  |  |
| HYP,HL,DIA | 0·26 | 0·37 | 0·30 | 0·34 |  |  |
| PNC,DEP,CSP | 0·39 | 0·21 | 0·24 | 0·41 |  |  |
| HF,CHD,AF | 0·32 | 0·36 | 0·22 | 0·34 |  |  |
| AST,COPD,PNC | 0·39 | 0·39 | 0·30 | 0·25 |  |  |

**Table 4b Pearson’s correlation coefficient (range = [-1 - 1], high values indicate a higher degree of similarity) for the similarities between corresponding clusters profiles (for 38 LTCs) in the training (rows) and test sets (columns).** Matched clusters are shaded in dark grey (secondary choices ae shaded in light grey).

| **18-44** | **DEP,ANX,PNC** | **AST,HL,PNC** | **IBS,DEP,AST** | **HYP,DIA,DEP** | **PSM,ALC,PNC** |  |
| --- | --- | --- | --- | --- | --- | --- |
| DEP,ANX,PNC | 1·00 | 0·41 | 0·36 | 0·22 | 0·27 |  |
| PNC,HL,HYP | 0·42 | 0·69 | 0·16 | 0·63 | 0·12 |  |
| AST,IBS,DEP | 0·29 | 0·74 | 0·44 | 0·24 | 0·15 |  |
| IBS,DEP,HL | 0·32 | 0·19 | 0·97 | 0·05 | 0·04 |  |
| PSM,ALC,DEP | 0·35 | 0·19 | 0·10 | 0·03 | 0·98 |  |
| **45-64** | **HYP,DIA,CHD** | **IBS,HL,AST** | **DEP,PNC,ANX** | **PNC,CHD,DIA** | **ALC,PSM,PNC** |  |
| HYP,DIA,PNC | 0·98 | 0·39 | 0·29 | 0·81 | 0·24 |  |
| IBS,HL,PNC | 0·35 | 0·90 | 0·52 | 0·46 | 0·19 |  |
| DEP,PNC,ANX | 0·24 | 0·46 | 1·00 | 0·55 | 0·56 |  |
| AST, PNC, COPD | 0·29 | 0·62 | 0·28 | 0·35 | 0·18 |  |
| ALC,PSM,PNC | 0·17 | 0·16 | 0·37 | 0·24 | 0·97 |  |
| **65-84** | **HYP,DIA,CKD** | **HL,IBS,DIV** | **CHD,AF,DIA** | **DEP,PNC,ANX** | **AST,COPD,PNC** | **PNC,DEP,CHD** |
| HYP,DIA,CKD | 1·00 | 0·62 | 0·78 | 0·56 | 0·55 | 0·74 |
| HL,PSD,IBS | 0·67 | 0·99 | 0·61 | 0·58 | 0·49 | 0·68 |
| DEP,PNC,ANX | 0·57 | 0·56 | 0·36 | 0·99 | 0·39 | 0·86 |
| CHD, DIA,AF | 0·78 | 0·52 | 1·00 | 0·38 | 0·40 | 0·74 |
| COPD,AST,PNC | 0·52 | 0·47 | 0·45 | 0·48 | 0·97 | 0·58 |
| PNC,CHD,DEP | 0·73 | 0·64 | 0·76 | 0·83 | 0·58 | 0·99 |
| **85+** | **HYP,CHD,HL** | **DEP,PNC,CSP** | **PNC,DEP,CSP** | **COPD,AST,PSD** |  |  |
| HYP,HL,DIA | 0·99 | 0·70 | 0·77 | 0·65 |  |  |
| PNC,DEP,CSP | 0·67 | 0·99 | 0·91 | 0·45 |  |  |
| CHD,AF,HF | 0·85 | 0·68 | 0·90 | 0·59 |  |  |
| AST,COPD,PNC | 0·68 | 0·63 | 0·74 | 0·94 |  |  |

**Table 5 Validation of the cluster solution derived from training set (80%) on the test set (20%) of multimorbid patients.**

| **Age 18-44** | **Entropy (training)=0·80; Entropy (test)=0·82** | |
| --- | --- | --- |
| **Training** | **Class size (%)** | **Top 3 most distinctive morbidities** |
| 1 | 32 | DEP,ANX,PNC |
| 2 | 23 | PNC,HL,HYP |
| 3 | 20 | AST,IBS,DEP |
| 4 | 18 | IBS,DEP,HL |
| 5 | 7 | PSM,ALC,DEP |
| **Test** |  |  |
| 1 | 35 | DEP,ANX,PNC |
| 2 | 26 | AST,HL,PNC |
| 3 | 23 | IBS,DEP,AST |
| 4 | 8 | HYP,DIA,DEP |
| 5 | 8 | PSM,ALC,PNC |
| **Age 45-64** | **Entropy (training)=0·68; Entropy (test)=0·65** | |
| **Training** | **Class size (%)** | **Top 3 most distinctive morbidities** |
| 1 | 37 | HYP,DIA,PNC |
| 2 | 24 | IBS,HL,PNC |
| 3 | 22 | DEP,PNC,ANX |
| 4 | 12 | AST, PNC, COPD |
| 5 | 4 | ALC,PSM,PNC |
| **Test** |  |  |
| 1 | 31 | HYP,DIA,CHD |
| 2 | 29 | IBS,HL,PNC |
| 3 | 23 | DEP,PNC,ANX |
| 4 | 11 | PNC,CHD,DIA |
| 5 | 5 | ALC,PSM,PNC |
| **Age 65-84** | **Entropy (training)=0·53; Entropy (test)=0·53** | |
| **Training** | **Class size (%)** | **Top 3 most distinctive morbidities** |
| 1 | 41 | HYP,DIA,CKD |
| 2 | 22 | HL,PSD,IBS |
| 3 | 14 | DEP,PNC,ANX |
| 4 | 11 | CHD, DIA,AF |
| 5 | 8 | COPD,AST,PNC |
| 6 | 5 | PNC,CHD,DEP |
| **Test** |  |  |
| 1 | 41 | HYP,DIA,CKD |
| 2 | 18 | HL,IBS,DIV |
| 3 | 15 | CHD,AF,DIA |
| 4 | 13 | PNC,DEP,CHD |
| 5 | 8 | AST,COPD,PNC |
| 6 | 6 | PNC,DEP,CHD |
| **Age 85+** | **Entropy (training)=0·56; Entropy (test)=0·59** | |
| **Training** | **Class size (%)** | **Top 3 most distinctive morbidities** |
| 1 | 58 | HYP,HL,DIA |
| 2 | 23 | PNC,DEP,CSP |
| 3 | 11 | CHD,AF,HF |
| 4 | 8 | AST,COPD,PNC |
| **Test** |  |  |
| 1 | 59 | HYP,CHD,HL |
| 2 | 22 | DEP,PNC,CSP |
| 3 | 11 | PNC,DEP,CSP |
| 4 | 8 | COPD,AST,PSD |

**Table 6 Relationship between multimorbidity cluster, patient characteristics and outcomes in the training set.** Cluster labels are defined by the three most distinctive morbidities in each cluster (where the difference between within-cluster prevalence and prevalence in age strata is the largest). Percent of greater deprivation is derived for the top 40% of IMD (Index of multiple deprivation in quintiles; categories 4 and 5). Number of GP consultations, hospital spells and repeat prescription in one year are derived. The amount of repeat prescriptions is defined in terms of BNF codes of prescriptions that have been repeated for at least four times in one year.

| **Multimorbidity clusters** | **% patients** | **% greater deprivation** | **Med· age** | **% female** | **% current smokers** | **Med· BMI** | **Med· # morbidities** | **Avg· # consultations** | **Avg· # hospital spells** | **Avg· # polypharmacy** | **% deaths in 2 years** | **% deaths in 5 years** |
| --- | --- | --- | --- | --- | --- | --- | --- | --- | --- | --- | --- | --- |
| **18-44** |  |  |  |  |  |  |  |  |  |  |  |  |
| DEP,ANX,PNC | 31·9 | 49·6 | 36·0 | 66·4 | 45·8 | 26·4 | 2·0 | 14·5 | 0·4 | 2·4 | 0·9 | 1·8 |
| PNC,HL,HYP | 22·8 | 45·5 | 38·0 | 52·4 | 27·3 | 27·5 | 2·0 | 11·9 | 0·6 | 2·5 | 1·0 | 2·7 |
| AST,IBS,DEP | 20·3 | 40·7 | 36·0 | 63·0 | 29·1 | 26·1 | 2·0 | 11·3 | 0·4 | 1·9 | 0·2 | 0·6 |
| IBS,DEP,HL | 18·3 | 37·1 | 37·0 | 76·7 | 28·4 | 25·3 | 2·0 | 10·6 | 0·4 | 1·2 | 0·2 | 0·4 |
| PSM,ALC,DEP | 6·7 | 63·3 | 36·5 | 27·7 | 76·2 | 24·4 | 2·0 | 10·7 | 0·4 | 1·3 | 1·8 | 3·9 |
| **45-64** |  |  |  |  |  |  |  |  |  |  |  |  |
| HYP,DIA,PNC | 37·2 | 37·7 | 58·0 | 41·8 | 20·5 | 30·0 | 2·0 | 11·5 | 0·5 | 4·1 | 1·6 | 4·4 |
| IBS,HL,PNC | 24·3 | 29·5 | 55·0 | 64·0 | 20·5 | 26·4 | 2·0 | 10·5 | 0·5 | 2·0 | 1·3 | 3·0 |
| DEP,PNC,ANX | 21·7 | 45·9 | 54·0 | 67·8 | 35·1 | 28·0 | 3·0 | 16·7 | 0·6 | 5·1 | 2·4 | 5·8 |
| AST, PNC, COPD | 12·3 | 35·1 | 55·0 | 60·5 | 20·2 | 28·1 | 2·0 | 12·6 | 0·4 | 3·4 | 1·0 | 2·7 |
| ALC,PSM,PNC | 4·4 | 57·2 | 53·0 | 31·1 | 63·3 | 25·2 | 3·0 | 10·7 | 0·5 | 2·4 | 4·5 | 12·5 |
| **65-84** |  |  |  |  |  |  |  |  |  |  |  |  |
| HYP,DIA,CKD | 41·0 | 30·4 | 74·0 | 54·5 | 10·1 | 28·0 | 3·0 | 12·4 | 0·6 | 4·5 | 4·7 | 13·2 |
| HL,PSD,IBS | 21·6 | 25·3 | 73·0 | 47·8 | 9·5 | 26·4 | 3·0 | 13·2 | 0·7 | 3·3 | 4·4 | 11·1 |
| DEP,PNC,ANX | 13·8 | 32·5 | 73·0 | 71·8 | 15·5 | 26·7 | 4·0 | 17·3 | 0·8 | 5·9 | 8·4 | 20·9 |
| CHD, DIA,AF | 10·8 | 33·3 | 76·0 | 29·5 | 12·0 | 27·7 | 4·0 | 16·8 | 1·1 | 5·9 | 11·3 | 28·8 |
| COPD,AST,PNC | 8·2 | 40·0 | 73·0 | 49·7 | 24·5 | 26·7 | 3·0 | 15·7 | 0·8 | 5·5 | 9·2 | 25·5 |
| PNC,CHD,DEP | 4·7 | 43·1 | 77·0 | 54·5 | 16·5 | 28·7 | 7·0 | 26·3 | 1·6 | 10·7 | 16·2 | 39·2 |
| **85+** |  |  |  |  |  |  |  |  |  |  |  |  |
| HYP,HL,DIA | 57·9 | 29·6 | 88·0 | 60·9 | 5·0 | 25·3 | 3·0 | 13·0 | 0·8 | 4·1 | 20·9 | 49·5 |
| PNC,DEP,CSP | 23·3 | 30·0 | 89·0 | 79·7 | 5·4 | 24·6 | 5·0 | 17·3 | 0·8 | 6·7 | 31·1 | 62·9 |
| CHD,AF,HF | 10·6 | 30·5 | 89·0 | 59·7 | 3·8 | 25·4 | 7·0 | 21·9 | 1·5 | 8·0 | 37·7 | 70·8 |
| AST,COPD,PNC | 8·3 | 30·2 | 88·0 | 59·2 | 8·2 | 25·0 | 5·0 | 19·6 | 1·1 | 6·9 | 28·0 | 56·5 |

**Table 7 Relationship between multimorbidity cluster, patient characteristics and outcomes in the test set.** Cluster labels are defined by the three most distinctive morbidities in each cluster (where the difference between within-cluster prevalence and prevalence in age strata is the largest). Percent of greater deprivation is derived for the top 40% of IMD (categories 4 and 5). Number of GP consultations, hospital spells and repeat prescription in one year are derived. The amount of repeat prescriptions is defined in terms of BNF codes of prescriptions that have been repeated for at least four times in one year.

| **Multimorbidity clusters** | **% patients** | **% greater deprivation** | **Med· age** | **% female** | **% current smokers** | **Med· BMI** | **Med· # morbidities** | **Avg· # consultations** | **Avg· # hospital spells** | **Avg· # polypharmacy** | **% deaths in 2 years** | **% deaths in 5 years** |
| --- | --- | --- | --- | --- | --- | --- | --- | --- | --- | --- | --- | --- |
| **18-44** |  |  |  |  |  |  |  |  |  |  |  |  |
| DEP,ANX,PNC | 35·2 | 49·9 | 36·0 | 65·4 | 44·7 | 25·9 | 2·0 | 14·0 | 0·4 | 2·4 | 0·6 | 1·1 |
| AST,HL,PNC | 26·3 | 40·3 | 35·0 | 53·7 | 24·9 | 26·1 | 2·0 | 10·8 | 0·4 | 2·0 | 0·6 | 2·1 |
| IBS,DEP,AST | 22·9 | 36·6 | 36·0 | 78·2 | 28·1 | 25·8 | 2·0 | 11·1 | 0·4 | 1·3 | 0·3 | 0·3 |
| HYP,DIA,DEP | 7·9 | 47·9 | 41·0 | 40·9 | 20·7 | 31·6 | 2·0 | 11·1 | 0·4 | 3·1 | 2·1 | 2·9 |
| PSM,ALC,PNC | 7·6 | 63·5 | 35·0 | 27·9 | 75·5 | 24·2 | 2·0 | 10·2 | 0·4 | 1·3 | 1·3 | 5·2 |
| **45-64** |  |  |  |  |  |  |  |  |  |  |  |  |
| HYP,DIA,CHD | 31·1 | 36·2 | 58·0 | 43·8 | 16·2 | 30·0 | 2·0 | 10·4 | 0·4 | 3·7 | 1·0 | 3·6 |
| IBS,HL,AST | 29·1 | 31·1 | 55·0 | 64·0 | 21·8 | 26·7 | 2·0 | 10·7 | 0·6 | 2·2 | 1·1 | 3·4 |
| DEP,PNC,ANX | 23·0 | 41·7 | 54·0 | 69·9 | 30·5 | 27·9 | 3·0 | 15·5 | 0·6 | 4·5 | 1·9 | 3·6 |
| PNC,CHD,DIA | 11·4 | 48·3 | 58·0 | 35·8 | 24·6 | 29·5 | 3·0 | 15·4 | 0·7 | 5·8 | 3·2 | 10·2 |
| ALC,PSM,PNC | 5·4 | 55·0 | 53·0 | 29·4 | 62·7 | 25·3 | 3·0 | 12·1 | 0·7 | 3·2 | 3·8 | 8·7 |
| **65-84** |  |  |  |  |  |  |  |  |  |  |  |  |
| HYP,DIA,CKD | 41·2 | 30·9 | 74·0 | 53·9 | 11·4 | 28·1 | 3·0 | 12·5 | 0·6 | 4·6 | 4·2 | 13·1 |
| HL,IBS,DIV | 17·7 | 25·6 | 73·0 | 53·4 | 9·4 | 26·1 | 3·0 | 13·1 | 0·7 | 3·1 | 4·8 | 12·7 |
| CHD,AF,DIA | 14·6 | 33·1 | 76·0 | 31·5 | 12·4 | 27·6 | 4·0 | 15·3 | 1·0 | 5·3 | 9·3 | 24·6 |
| DEP,PNC,ANX | 13·3 | 34·2 | 73·0 | 69·2 | 17·2 | 26·3 | 4·0 | 16·9 | 0·9 | 5·8 | 9·1 | 23·4 |
| AST,COPD,PNC | 7·7 | 31·1 | 72·0 | 54·3 | 17·5 | 26·7 | 3·0 | 15·9 | 0·7 | 5·1 | 5·9 | 19·2 |
| PNC,DEP,CHD | 5·6 | 42·9 | 76·0 | 56·2 | 14·9 | 29·0 | 7·0 | 24·5 | 1·0 | 10·4 | 13·8 | 32·9 |
| **85+** |  |  |  |  |  |  |  |  |  |  |  |  |
| HYP,CHD,HL | 58·6 | 29·5 | 88·0 | 60·2 | 4·9 | 25·5 | 3·0 | 14·2 | 0·8 | 4·4 | 21·1 | 49·9 |
| DEP,PNC,CSP | 22·4 | 27·0 | 89·0 | 85·3 | 4·0 | 24·4 | 4·0 | 15·2 | 0·6 | 5·9 | 28·3 | 63·9 |
| PNC,DEP,CSP | 10·6 | 32·6 | 89·0 | 62·5 | 6·9 | 24·9 | 8·0 | 22·8 | 1·1 | 9·3 | 36·8 | 73·2 |
| COPD,AST,PSD | 8·5 | 33·7 | 88·0 | 49·0 | 8·7 | 24·9 | 4·0 | 16·8 | 1·0 | 5·3 | 26·9 | 56·3 |

# Association between multimorbidity clusters, health service utilisation and mortality

## Relating multimorbidity clusters to outcomes

In this section, we present results for regression models of for each age strata. Outcome measures are health service utilisation and mortality. Service utilisation is measured by three variables: the number of GP consultations in one year, the number of hospitalisation spells in one year and the amount of polypharmacy (in terms of BNF codes of prescriptions that have been repeated for at least four times) in one year since January 2012. Mortality is measured by 2-year mortality and 5-year mortality; both are binary variables. Separate regression models for each outcome given multimorbidity clusters is estimated, adjusted for demographics, i.e. gender (ref. male), socioeconomic status (measured by IMD, ref. least deprived), smoking status (ref. never smoker), BMI and age. Among the multimorbidity clusters, the cluster with the lowest impact on the outcomes of interest (in that age strata) is chosen as the reference cluster that all other clusters were compared to. Generalised linear models are fitted for each outcome. Specifically, to account for the dispersion of the outcome variables, negative binomial models are fitted for service utilisation outcomes (coefficients are incident rate ratios, IRR), and logit models (coefficients are odds ratios, OR) are fitted for mortality outcomes. We present below results for the training set only for clarity purposes (results for test sets are very similar to that in the training set, except that for some demographics the effects are non-significant due to the reduced sample size in the test sets). Additionally, we have conducted a set of sensitivity analyses using the entire sample (including both multimorbid and non-multimorbid patients) to understand the impact of multimorbidity clusters on service use and health outcomes, relative to the non-multimorbid patients. We caution interested readers not to interpret these results causally because 1) we have not allowed for non-linear relationships and interactions between predictors and 2) there is likely to be residual confounding.

Summary of findings from regression models:

In general, across age groups, we find that the association between multimorbidity clusters and outcomes are obviously larger than that of other demographics characteristics. Stratified by age groups, the association of multimorbidity clusters with outcomes remains strong and statistically significant (p<0.05 in almost all clusters). Compared to male patients, females generally have better mortality outcomes across all ages. The impact of poor socioeconomic circumstances on these outcomes is greater and statistically significant in the younger population (below 65 years old, and is particularly strong in terms of NHS service use) and gradually diminishes in older population (aged 85 and above). Smoking, in earlier years (<65 years old) is significantly related to higher service use and in the older population (aged 65 and above), is significantly related to a higher likelihood of mortality.

**Table 8 Association between of multimorbidity clusters and NHS service utilisation and mortality (age strata 18-44).** Coefficients are reported in incidence rate ratios (for negative binomial models) and odds ratios (for logit models) with 95% confidence intervals.

| **Predictors** | **GP consultation** | **Hospitalisation** | **Repeat prescription** | **2-year mortality** | **5-year mortality** |
| --- | --- | --- | --- | --- | --- |
|  | **IRR (95% CI)** | **IRR (95% CI)** | **IRR (95% CI)** | **OR (95% CI)** | **OR (95% CI)** |
| Multimorbidity clusters | | | | | |
| `IBS,DEP,HL` | 1 | 1 | 1 | 1 | 1 |
| `DEP,ANX,PNC` | 1·35 ^***^ | 1·04 | 1·87 ^***^ | 1·01 ^***^ | 1·01 ^***^ |
|  | (1·28 – 1·43) | (0·90 – 1·20) | (1·74 – 2·02) | (1·00 – 1·01) | (1·00 – 1·02) |
| `PNC,HL,HYP` | 1·18 ^***^ | 1·77 ^***^ | 1·90 ^***^ | 1·01 ^**^ | 1·02 ^***^ |
|  | (1·11 – 1·25) | (1·53 – 2·05) | (1·76 – 2·06) | (1·00 – 1·01) | (1·01 – 1·03) |
| `AST,IBS,DEP` | 1·08 ^**^ | 0·90 | 1·56 ^***^ | 1·00 | 1·00 |
|  | (1·02 – 1·15) | (0·77 – 1·05) | (1·44 – 1·69) | (1·00 – 1·00) | (0·99 – 1·01) |
| `PSM,ALC,DEP` | 1·13 ^***^ | 1·12 | 1·04 | 1·01 ^***^ | 1·03 ^***^ |
|  | (1·04 – 1·24) | (0·89 – 1·41) | (0·92 – 1·17) | (1·00 – 1·02) | (1·01 – 1·04) |
| Gender | | | | | |
| Male | 1 | 1 | 1 | 1 | 1 |
| Female | 1·36 ^***^ | 1·41 ^***^ | 0·97 | 1·00 ^***^ | 0·99 ^***^ |
|  | (1·31 – 1·42) | (1·27 – 1·56) | (0·92 – 1·02) | (0·99 – 1·00) | (0·99 – 1·00) |
| Index of multiple deprivation (in quintiles) | | | | | |
| 1 (least deprived) | 1 | 1 | 1 | 1 | 1 |
| 2 | 1·03 | 1·28 ^***^ | 1·08 ^*^ | 1·00 | 1·00 |
|  | (0·96 – 1·09) | (1·09 – 1·51) | (0·99 – 1·17) | (0·99 – 1·00) | (0·99 – 1·01) |
| 3 | 1·03 | 1·42 ^***^ | 1·20 ^***^ | 1·00 | 1·00 |
|  | (0·97 – 1·09) | (1·21 – 1·66) | (1·11 – 1·30) | (1·00 – 1·01) | (1·00 – 1·01) |
| 4 | 1·06 ^*^ | 1·42 ^***^ | 1·27 ^***^ | 1·00 | 1·00 |
|  | (1·00 – 1·12) | (1·21 – 1·66) | (1·18 – 1·37) | (0·99 – 1·00) | (0·99 – 1·01) |
| 5 (most deprived) | 1·05 | 1·35 ^***^ | 1·33 ^***^ | 1·00 | 1·00 |
|  | (0·99 – 1·11) | (1·15 – 1·58) | (1·23 – 1·44) | (0·99 – 1·00) | (1·00 – 1·01) |
| Smoking status | | | | | |
| Non-smoker | 1 | 1 | 1 | 1 | 1 |
| Current smoker | 1·11 ^***^ | 1·15 ^**^ | 1·07 ^**^ | 1·00 ^*^ | 1·01 ^**^ |
|  | (1·07 – 1·16) | (1·03 – 1·28) | (1·01 – 1·13) | (1·00 – 1·01) | (1·00 – 1·01) |
| Ex-smoker | 1·05 ^*^ | 1·22 ^***^ | 1·05 | 1·00 ^**^ | 1·01 ^**^ |
|  | (0·99 – 1·10) | (1·08 – 1·39) | (0·98 – 1·12) | (1·00 – 1·01) | (1·00 – 1·01) |
| BMI | 1·01 ^***^ | 1·00 | 1·02 ^***^ | 1·00 | 1·00 |
|  | (1·01 – 1·01) | (0·99 – 1·01) | (1·02 – 1·03) | (1·00 – 1·00) | (1·00 – 1·00) |
| Age | 1·00 | 0·98 ^***^ | 1·03 ^***^ | 1·00 ^**^ | 1·00 ^***^ |
|  | (1·00 – 1·00) | (0·97 – 0·99) | (1·02 – 1·03) | (1·00 – 1·00) | (1·00 – 1·00) |
| * p<0·1   ** p<0·05   *** p<0·01 | | | | | |

**Table 9 Association between of multimorbidity clusters and NHS service utilisation and mortality (age strata 45-64**). Coefficients are reported in incidence rate ratios (for negative binomial models) and odds ratios (for logit models) with 95% confidence intervals.

| **Predictors** | **GP consultation** | **Hospitalisation** | **Repeat prescription** | **2-year mortality** | **5-year mortality** |
| --- | --- | --- | --- | --- | --- |
|  | **IRR (95% CI)** | **IRR (95% CI)** | **IRR (95% CI)** | **OR (95% CI)** | **OR (95% CI)** |
| Multimorbidity clusters | | | | | |
| `IBS,HL,PNC` | 1 | 1 | 1 | 1 | 1 |
| `HYP,DIA,PNC` | 1·05 ^***^ | 1·06 | 1·76 ^***^ | 1·00 | 1·01 ^*^ |
|  | (1·02 – 1·09) | (0·97 – 1·15) | (1·70 – 1·82) | (1·00 – 1·00) | (1·00 – 1·01) |
| `DEP,PNC,ANX` | 1·52 ^***^ | 1·31 ^***^ | 2·37 ^***^ | 1·01 ^***^ | 1·02 ^***^ |
|  | (1·47 – 1·58) | (1·20 – 1·44) | (2·29 – 2·46) | (1·01 – 1·01) | (1·02 – 1·03) |
| `AST,COPD,PNC` | 1·16 ^***^ | 0·92 | 1·60 ^***^ | 1·00 | 1·00 |
|  | (1·12 – 1·21) | (0·83 – 1·03) | (1·53 – 1·67) | (0·99 – 1·00) | (0·99 – 1·01) |
| `ALC,PSM,PNC` | 1·05 | 0·99 | 1·19 ^***^ | 1·02 ^***^ | 1·08 ^***^ |
|  | (0·98 – 1·12) | (0·84 – 1·17) | (1·11 – 1·27) | (1·01 – 1·03) | (1·07 – 1·10) |
| Gender | | | | | |
| Male | 1 | 1 | 1 | 1 | 1 |
| Female | 1·12 ^***^ | 0·96 | 0·97 ^**^ | 0·99 ^***^ | 0·99 ^***^ |
|  | (1·10 – 1·15) | (0·90 – 1·02) | (0·95 – 1·00) | (0·99 – 1·00) | (0·98 – 0·99) |
| Index of multiple deprivation (in quintiles) | | | | | |
| 1 (least deprived) | 1 | 1 | 1 | 1 | 1 |
| 2 | 1·00 | 1·21 ^***^ | 1·07 ^***^ | 1·00 | 1·01 ^**^ |
|  | (0·96 – 1·03) | (1·10 – 1·33) | (1·03 – 1·11) | (1·00 – 1·01) | (1·00 – 1·02) |
| 3 | 1·04 ^**^ | 1·15 ^***^ | 1·12 ^***^ | 1·00 ^*^ | 1·01 ^***^ |
|  | (1·01 – 1·08) | (1·05 – 1·27) | (1·08 – 1·16) | (1·00 – 1·01) | (1·00 – 1·02) |
| 4 | 1·05 ^**^ | 1·23 ^***^ | 1·22 ^***^ | 1·01 ^**^ | 1·01 ^***^ |
|  | (1·01 – 1·09) | (1·12 – 1·36) | (1·17 – 1·27) | (1·00 – 1·01) | (1·01 – 1·02) |
| 5 (most deprived) | 1·13 ^***^ | 1·33 ^***^ | 1·36 ^***^ | 1·01 ^***^ | 1·03 ^***^ |
|  | (1·08 – 1·17) | (1·20 – 1·47) | (1·31 – 1·41) | (1·01 – 1·02) | (1·02 – 1·03) |
| Smoking status | | | | | |
| Non-smoker | 1 | 1 | 1 | 1 | 1 |
| Current smoker | 1·04 ^**^ | 1·04 | 1·12 ^***^ | 1·01 ^***^ | 1·04 ^***^ |
|  | (1·01 – 1·07) | (0·96 – 1·12) | (1·08 – 1·15) | (1·01 – 1·02) | (1·03 – 1·04) |
| Ex-smoker | 1·06 ^***^ | 1·04 | 1·12 ^***^ | 1·00 | 1·01 ^**^ |
|  | (1·03 – 1·09) | (0·96 – 1·12) | (1·09 – 1·15) | (1·00 – 1·01) | (1·00 – 1·01) |
| BMI | 1·01 ^***^ | 1·00 | 1·02 ^***^ | 1·00 ^***^ | 1·00 ^**^ |
|  | (1·01 – 1·01) | (0·99 – 1·00) | (1·02 – 1·02) | (1·00 – 1·00) | (1·00 – 1·00) |
| Age | 1·01 ^***^ | 1·01 ^***^ | 1·03 ^***^ | 1·00 ^***^ | 1·00 ^***^ |
|  | (1·00 – 1·01) | (1·01 – 1·02) | (1·02 – 1·03) | (1·00 – 1·00) | (1·00 – 1·00) |
| * p<0·1   ** p<0·05   *** p<0·01 | | | | | |

**Table 10 Association between of multimorbidity clusters and NHS service utilisation and mortality (age strata 65-84).** Coefficients are reported in incidence rate ratios (for negative binomial models) and odds ratios (for logit models) with 95% confidence intervals.

| **Predictors** | **GP consultation** | **Hospitalisation** | **Repeat prescription** | **2-year mortality** | **5-year mortality** |
| --- | --- | --- | --- | --- | --- |
|  | **IRR (95% CI)** | **IRR (95% CI)** | **IRR (95% CI)** | **OR (95% CI)** | **OR (95% CI)** |
| Multimorbidity clusters | | | | | |
| `HL,PSD,IBS` | 1 | 1 | 1 | 1 | 1 |
| `HYP,DIA,CKD` | 0·92 ^***^ | 0·88 ^***^ | 1·29 ^***^ | 1·00 | 1·02 ^***^ |
|  | (0·90 – 0·95) | (0·83 – 0·93) | (1·26 – 1·32) | (1·00 – 1·01) | (1·01 – 1·03) |
| `DEP,PNC,ANX` | 1·30 ^***^ | 1·20 ^***^ | 1·72 ^***^ | 1·04 ^***^ | 1·10 ^***^ |
|  | (1·25 – 1·34) | (1·11 – 1·30) | (1·67 – 1·77) | (1·03 – 1·05) | (1·09 – 1·12) |
| `CHD,AF,DIA` | 1·24 ^***^ | 1·51 ^***^ | 1·65 ^***^ | 1·05 ^***^ | 1·14 ^***^ |
|  | (1·19 – 1·29) | (1·40 – 1·64) | (1·59 – 1·70) | (1·05 – 1·06) | (1·13 – 1·16) |
| `COPD,AST,PNC` | 1·19 ^***^ | 1·09 ^*^ | 1·56 ^***^ | 1·04 ^***^ | 1·13 ^***^ |
|  | (1·14 – 1·24) | (1·00 – 1·20) | (1·51 – 1·62) | (1·03 – 1·05) | (1·12 – 1·15) |
| `PNC,CHD,DEP` | 1·92 ^***^ | 2·15 ^***^ | 2·88 ^***^ | 1·10 ^***^ | 1·26 ^***^ |
|  | (1·82 – 2·02) | (1·94 – 2·40) | (2·77 – 3·00) | (1·09 – 1·12) | (1·24 – 1·29) |
| Gender | | | | | |
| Male | 1 | 1 | 1 | 1 | 1 |
| Female | 1·04 ^***^ | 0·92 ^***^ | 1·00 | 0·99 ^***^ | 0·96 ^***^ |
|  | (1·02 – 1·06) | (0·88 – 0·97) | (0·99 – 1·02) | (0·98 – 0·99) | (0·95 – 0·97) |
| Index of multiple deprivation (in quintiles) | | | | | |
| 1 (least deprived) | 1 | 1 | 1 | 1 | 1 |
| 2 | 0·96 ^**^ | 1·03 | 1·02 | 1·00 | 1·00 |
|  | (0·94 – 0·99) | (0·97 – 1·10) | (1·00 – 1·05) | (1·00 – 1·01) | (0·99 – 1·01) |
| 3 | 0·97 ^**^ | 1·11 ^***^ | 1·05 ^***^ | 1·01 ^*^ | 1·01 ^**^ |
|  | (0·94 – 1·00) | (1·04 – 1·18) | (1·02 – 1·08) | (1·00 – 1·01) | (1·00 – 1·02) |
| 4 | 0·95 ^***^ | 1·14 ^***^ | 1·11 ^***^ | 1·02 ^***^ | 1·04 ^***^ |
|  | (0·92 – 0·99) | (1·06 – 1·22) | (1·08 – 1·14) | (1·01 – 1·02) | (1·03 – 1·05) |
| 5 (most deprived) | 0·94 ^***^ | 1·27 ^***^ | 1·14 ^***^ | 1·02 ^***^ | 1·04 ^***^ |
|  | (0·91 – 0·97) | (1·18 – 1·37) | (1·11 – 1·18) | (1·01 – 1·03) | (1·03 – 1·05) |
| Smoking status | | | | | |
| Non-smoker | 1 | 1 | 1 | 1 | 1 |
| Current smoker | 0·99 | 1·03 | 1·02 | 1·04 ^***^ | 1·10 ^***^ |
|  | (0·95 – 1·02) | (0·96 – 1·11) | (0·99 – 1·05) | (1·03 – 1·05) | (1·09 – 1·12) |
| Ex-smoker | 1·03 ^**^ | 1·04 | 1·08 ^***^ | 1·01 ^***^ | 1·03 ^***^ |
|  | (1·01 – 1·05) | (0·99 – 1·09) | (1·06 – 1·10) | (1·01 – 1·02) | (1·02 – 1·04) |
| BMI | 1·01 ^***^ | 1·00 ^*^ | 1·02 ^***^ | 1·00 ^***^ | 1·00 ^***^ |
|  | (1·01 – 1·01) | (0·99 – 1·00) | (1·02 – 1·02) | (1·00 – 1·00) | (1·00 – 1·00) |
| Age | 1·01 ^***^ | 1·02 ^***^ | 1·01 ^***^ | 1·00 ^***^ | 1·01 ^***^ |
|  | (1·01 – 1·01) | (1·01 – 1·02) | (1·01 – 1·01) | (1·00 – 1·01) | (1·01 – 1·02) |
| * p<0·1   ** p<0·05   *** p<0·01 | | | | | |

**Table 11 Association between of multimorbidity clusters and NHS service utilisation and mortality (age strata 85+).** Coefficients are reported in incidence rate ratios (for negative binomial models) and odds ratios (for logit models) with 95% confidence intervals.

| **Predictors** | **GP consultation** | **Hospitalisation** | **Repeat prescription** | **2-year mortality** | **5-year mortality** |
| --- | --- | --- | --- | --- | --- |
|  | **IRR (95% CI)** | **IRR (95% CI)** | **IRR (95% CI)** | **OR (95% CI)** | **OR (95% CI)** |
| Multimorbidity clusters | | | | | |
| `HYP,HL,DIA` | 1 | 1 | 1 | 1 | 1 |
| `PNC,DEP,CSP` | 1·38 ^***^ | 1·22 ^***^ | 1·66 ^***^ | 1·09 ^***^ | 1·12 ^***^ |
|  | (1·30 – 1·46) | (1·11 – 1·34) | (1·58 – 1·75) | (1·06 – 1·11) | (1·09 – 1·15) |
| `HF,CHD,AF` | 1·68 ^***^ | 1·97 ^***^ | 1·93 ^***^ | 1·18 ^***^ | 1·23 ^***^ |
|  | (1·56 – 1·82) | (1·76 – 2·21) | (1·82 – 2·06) | (1·15 – 1·22) | (1·19 – 1·27) |
| `AST,COPD,PNC` | 1·49 ^***^ | 1·39 ^***^ | 1·66 ^***^ | 1·09 ^***^ | 1·09 ^***^ |
|  | (1·37 – 1·62) | (1·22 – 1·59) | (1·55 – 1·78) | (1·06 – 1·12) | (1·05 – 1·13) |
| Gender | | | | | |
| Male | 1 | 1 | 1 | 1 | 1 |
| Female | 0·92 ^***^ | 0·85 ^***^ | 1·04 | 0·95 ^***^ | 0·93 ^***^ |
|  | (0·88 – 0·97) | (0·79 – 0·92) | (0·99 – 1·08) | (0·93 – 0·97) | (0·91 – 0·95) |
| Index of multiple deprivation (in quintiles) | | | | | |
| 1 (least deprived) | 1 | 1 | 1 | 1 | 1 |
| 2 | 0·94 ^*^ | 0·96 | 1·02 | 0·99 | 0·99 |
|  | (0·88 – 1·00) | (0·86 – 1·07) | (0·96 – 1·08) | (0·96 – 1·01) | (0·97 – 1·02) |
| 3 | 0·91 ^***^ | 1·13 ^**^ | 0·98 | 1·01 | 1·00 |
|  | (0·85 – 0·98) | (1·01 – 1·26) | (0·93 – 1·04) | (0·98 – 1·03) | (0·98 – 1·03) |
| 4 | 0·90 ^***^ | 0·97 | 0·99 | 1·01 | 1·01 |
|  | (0·84 – 0·97) | (0·86 – 1·09) | (0·93 – 1·05) | (0·98 – 1·03) | (0·98 – 1·04) |
| 5 (most deprived) | 0·89 ^***^ | 1·04 | 1·09 ^**^ | 1·01 | 1·05 ^***^ |
|  | (0·82 – 0·96) | (0·92 – 1·19) | (1·02 – 1·16) | (0·98 – 1·04) | (1·01 – 1·08) |
| Smoking status | | | | | |
| Non-smoker | 1 | 1 | 1 | 1 | 1 |
| Current smoker | 0·96 | 1·04 | 0·91 ^**^ | 1·07 ^***^ | 1·12 ^***^ |
|  | (0·87 – 1·07) | (0·88 – 1·23) | (0·83 – 1·00) | (1·03 – 1·11) | (1·07 – 1·17) |
| Ex-smoker | 1·04 | 0·99 | 1·06 ^***^ | 1·01 | 1·04 ^***^ |
|  | (0·99 – 1·09) | (0·91 – 1·07) | (1·02 – 1·11) | (0·99 – 1·03) | (1·02 – 1·06) |
| BMI | 1·00 | 0·99 ^*^ | 1·01 ^***^ | 0·99 ^***^ | 0·99 ^***^ |
|  | (0·99 – 1·00) | (0·99 – 1·00) | (1·01 – 1·01) | (0·99 – 0·99) | (0·99 – 1·00) |
| Age | 0·98 ^***^ | 0·98 ^***^ | 0·98 ^***^ | 1·02 ^***^ | 1·03 ^***^ |
|  | (0·98 – 0·99) | (0·97 – 0·99) | (0·98 – 0·99) | (1·02 – 1·02) | (1·03 – 1·03) |
| * p<0·1   ** p<0·05   *** p<0·01 | | | | | |

## Relating demographics to multimorbidity clusters

**Table 12 Association between of multimorbidity clusters and patient demographics (age strata 18-44)**. Coefficients are reported in odds ratios (for logit models) with 95% confidence intervals.

| **Predictors** | **DEP,ANX,PNC** | **PNC,HL,HYP** | **AST,IBS,DEP** | **IBS,DEP,HL** | **PSM,ALC,DEP** |
| --- | --- | --- | --- | --- | --- |
|  | **OR (95% CI)** | **OR (95% CI)** | **OR (95% CI)** | **OR (95% CI)** | **OR (95% CI)** |
| Gender | | | | |  |
| Male | 1 | 1 | 1 | 1 | 1 |
| Female | 1·08 ^***^ | 0·90 ^***^ | 1·00 | 1·12 ^***^ | 0·92 ^***^ |
|  | (1·06 – 1·10) | (0·88 – 0·91) | (0·98 – 1·02) | (1·10 – 1·13) | (0·91 – 0·93) |
| Index of multiple deprivation (in quintiles) | | | | |  |
| 1 (least deprived) | 1 | 1 | 1 | 1 | 1 |
| 2 | 0·98 | 1·01 | 1·01 | 1·00 | 1·00 |
|  | (0·96 – 1·01) | (0·98 – 1·04) | (0·98 – 1·03) | (0·97 – 1·02) | (0·99 – 1·02) |
| 3 | 1·01 | 1·02 | 0·99 | 0·98 | 1·00 |
|  | (0·98 – 1·03) | (0·99 – 1·04) | (0·97 – 1·02) | (0·96 – 1·00) | (0·99 – 1·02) |
| 4 | 1·03 ^**^ | 1·01 | 0·98 | 0·96 ^***^ | 1·02 ^***^ |
|  | (1·00 – 1·06) | (0·99 – 1·04) | (0·96 – 1·01) | (0·93 – 0·98) | (1·00 – 1·03) |
| 5 (most deprived) | 1·03 ^**^ | 1·02 ^*^ | 0·96 ^***^ | 0·94 ^***^ | 1·05 ^***^ |
|  | (1·00 – 1·06) | (1·00 – 1·05) | (0·93 – 0·98) | (0·92 – 0·96) | (1·04 – 1·07) |
| Smoking status | | | | |  |
| Non-smoker | 1 | 1 | 1 | 1 | 1 |
| Current smoker | 1·14 ^***^ | 0·89 ^***^ | 0·94 ^***^ | 0·95 ^***^ | 1·11 ^***^ |
|  | (1·12 – 1·17) | (0·87 – 0·90) | (0·92 – 0·95) | (0·93 – 0·96) | (1·10 – 1·12) |
| Ex-smoker | 1·05 ^***^ | 0·93 ^***^ | 0·99 | 1·00 | 1·04 ^***^ |
|  | (1·02 – 1·07) | (0·91 – 0·95) | (0·97 – 1·01) | (0·98 – 1·02) | (1·03 – 1·05) |
| BMI | 1·00 ^***^ | 1·00 ^***^ | 1·00 | 1·00 ^***^ | 1·00 ^***^ |
|  | (1·00 – 1·00) | (1·00 – 1·01) | (1·00 – 1·00) | (0·99 – 1·00) | (1·00 – 1·00) |
| Age | 1·00 ^***^ | 1·00 ^***^ | 1·00 ^***^ | 1·00 ^***^ | 1·00 |
|  | (1·00 – 1·00) | (1·00 – 1·01) | (0·99 – 1·00) | (1·00 – 1·00) | (1·00 – 1·00) |
| * p<0·1   ** p<0·05   *** p<0·01 | | | | | |

**Table 13 Association between of multimorbidity clusters and patient demographics (age strata 45-64)**. Coefficients are reported in odds ratios (for logit models) with 95% confidence intervals.

| **Predictors** | **HYP,DIA,PNC** | **IBS,HL,PNC** | **DEP,PNC,ANX** | **AST,COPD,PNC** | **ALC,PSM,PNC** |
| --- | --- | --- | --- | --- | --- |
|  | **OR (95% CI)** | **OR (95% CI)** | **OR (95% CI)** | **OR (95% CI)** | **OR (95% CI)** |
| Gender | | | | |  |
| Male | 1 | 1 | 1 | 1 | 1 |
| Female | 0·82 ^***^ | 1·09 ^***^ | 1·12 ^***^ | 1·03 ^***^ | 0·96 ^***^ |
|  | (0·81 – 0·83) | (1·08 – 1·10) | (1·11 – 1·13) | (1·02 – 1·04) | (0·96 – 0·97) |
| Index of multiple deprivation (in quintiles) | | | | |  |
| 1 (least deprived) | 1 | 1 | 1 | 1 | 1 |
| 2 | 1·01 | 0·99 ^*^ | 1·02 ^**^ | 0·99 | 0·99 |
|  | (0·99 – 1·02) | (0·97 – 1·00) | (1·00 – 1·03) | (0·98 – 1·01) | (0·99 – 1·00) |
| 3 | 1·01 | 0·98 ^***^ | 1·02 ^***^ | 0·99 ^*^ | 1·01 |
|  | (0·99 – 1·03) | (0·96 – 0·99) | (1·01 – 1·04) | (0·98 – 1·00) | (1·00 – 1·01) |
| 4 | 1·01 | 0·94 ^***^ | 1·05 ^***^ | 0·99 ^**^ | 1·02 ^***^ |
|  | (0·99 – 1·03) | (0·92 – 0·95) | (1·04 – 1·07) | (0·97 – 1·00) | (1·01 – 1·02) |
| 5 (most deprived) | 1·00 | 0·92 ^***^ | 1·08 ^***^ | 0·98 ^***^ | 1·03 ^***^ |
|  | (0·98 – 1·01) | (0·90 – 0·93) | (1·07 – 1·10) | (0·97 – 0·99) | (1·03 – 1·04) |
| Smoking status | | | | |  |
| Non-smoker | 1 | 1 | 1 | 1 | 1 |
| Current smoker | 0·94 ^***^ | 0·92 ^***^ | 1·11 ^***^ | 0·97 ^***^ | 1·08 ^***^ |
|  | (0·93 – 0·95) | (0·91 – 0·93) | (1·09 – 1·12) | (0·96 – 0·98) | (1·07 – 1·08) |
| Ex-smoker | 0·98 ^**^ | 0·97 ^***^ | 1·03 ^***^ | 1·01 ^*^ | 1·01 ^***^ |
|  | (0·97 – 1·00) | (0·96 – 0·98) | (1·02 – 1·04) | (1·00 – 1·02) | (1·01 – 1·02) |
| BMI | 1·01 ^***^ | 0·99 ^***^ | 1·00 | 1·00 ^**^ | 1·00 ^***^ |
|  | (1·01 – 1·02) | (0·99 – 0·99) | (1·00 – 1·00) | (1·00 – 1·00) | (1·00 – 1·00) |
| Age | 1·02 ^***^ | 1·00 ^***^ | 0·99 ^***^ | 1·00 ^***^ | 1·00 ^***^ |
|  | (1·01 – 1·02) | (1·00 – 1·00) | (0·99 – 0·99) | (1·00 – 1·00) | (1·00 – 1·00) |
| * p<0·1   ** p<0·05   *** p<0·01 | | | | | |

**Table 14 Association between of multimorbidity clusters and patient demographics (age strata 65-84)**. Coefficients are reported in odds ratios (for logit models) with 95% confidence intervals.

| **Predictors** | **HYP,DIA,CKD** | **HL,PSD,IBS** | **DEP,PNC,ANX** | | **CHD,AF,DIA** | | **COPD,AST,PNC** | | **PNC,CHD,DEP** | |  |
| --- | --- | --- | --- | --- | --- | --- | --- | --- | --- | --- | --- |
|  | **OR (95% CI)** | **OR (95% CI)** | | **OR (95% CI)** | | **OR (95% CI)** | | **OR (95% CI)** | | **OR (95% CI)** | |
| Gender | | | | | | | |  | |  | |
| Male | 1 | 1 | | 1 | | 1 | | 1 | | 1 | |
| Female | 1·02 ^***^ | 0·96 ^***^ | | 1·11 ^***^ | | 0·91 ^***^ | | 1·01 ^***^ | | 1·01 ^***^ | |
|  | (1·01 – 1·03) | (0·95 – 0·97) | | (1·11 – 1·12) | | (0·90 – 0·91) | | (1·00 – 1·01) | | (1·00 – 1·01) | |
| Index of multiple deprivation (in quintiles) | | | | | | | |  | |  | |
| 1 (least deprived) | 1 | 1 | | 1 | | 1 | | 1 | | 1 | |
| 2 | 1·01 | 0·99 ^**^ | | 0·99 ^*^ | | 1·00 | | 1·00 | | 1·01 ^**^ | |
|  | (1·00 – 1·03) | (0·97 – 1·00) | | (0·98 – 1·00) | | (0·99 – 1·01) | | (1·00 – 1·01) | | (1·00 – 1·01) | |
| 3 | 1·00 | 0·98 ^***^ | | 1·00 | | 1·00 | | 1·01 ^**^ | | 1·01 ^***^ | |
|  | (0·99 – 1·02) | (0·97 – 0·99) | | (0·99 – 1·01) | | (0·99 – 1·01) | | (1·00 – 1·02) | | (1·00 – 1·02) | |
| 4 | 1·00 | 0·95 ^***^ | | 1·00 | | 1·01 | | 1·02 ^***^ | | 1·02 ^***^ | |
|  | (0·98 – 1·02) | (0·94 – 0·96) | | (0·99 – 1·01) | | (1·00 – 1·02) | | (1·02 – 1·03) | | (1·01 – 1·03) | |
| 5 (most deprived) | 0·97 ^***^ | 0·94 ^***^ | | 1·00 | | 1·02 ^***^ | | 1·03 ^***^ | | 1·04 ^***^ | |
|  | (0·96 – 0·99) | (0·93 – 0·95) | | (0·99 – 1·02) | | (1·01 – 1·03) | | (1·02 – 1·04) | | (1·03 – 1·05) | |
| Smoking status | | | | | | | |  | |  | |
| Non-smoker | 1 | 1 | | 1 | | 1 | | 1 | | 1 | |
| Current smoker | 0·90 ^***^ | 0·91 ^***^ | | 1·02 ^***^ | | 1·02 ^***^ | | 1·13 ^***^ | | 1·04 ^***^ | |
|  | (0·88 – 0·91) | (0·90 – 0·93) | | (1·01 – 1·03) | | (1·01 – 1·03) | | (1·12 – 1·14) | | (1·03 – 1·04) | |
| Ex-smoker | 0·92 ^***^ | 0·96 ^***^ | | 0·99 ^*^ | | 1·03 ^***^ | | 1·08 ^***^ | | 1·03 ^***^ | |
|  | (0·91 – 0·93) | (0·95 – 0·97) | | (0·99 – 1·00) | | (1·02 – 1·04) | | (1·08 – 1·09) | | (1·02 – 1·03) | |
| BMI | 1·01 ^***^ | 0·99 ^***^ | | 1·00 ^***^ | | 1·00 ^***^ | | 1·00 ^***^ | | 1·00 ^***^ | |
|  | (1·01 – 1·01) | (0·99 – 0·99) | | (1·00 – 1·00) | | (1·00 – 1·00) | | (1·00 – 1·00) | | (1·00 – 1·00) | |
| Age | 1·00 | 1·00 ^***^ | | 1·00 ^***^ | | 1·01 ^***^ | | 1·00 ^***^ | | 1·00 ^***^ | |
|  | (1·00 – 1·00) | (0·99 – 1·00) | | (1·00 – 1·00) | | (1·01 – 1·01) | | (1·00 – 1·00) | | (1·00 – 1·00) | |
| * p<0·1   ** p<0·05   *** p<0·01 | | | | | | | | | | | |

**Table 15 Association between of multimorbidity clusters and patient demographics (age strata 85+)**. Coefficients are reported in odds ratios (for logit models) with 95% confidence intervals.

| **Predictors** | **HYP,HL,DIA** | **PNC,DEP,CSP** | **HF,CHD,AF** | **AST,COPD,PNC** |
| --- | --- | --- | --- | --- |
|  | **OR (95% CI)** | **OR (95% CI)** | **OR (95% CI)** | **OR (95% CI)** |
| Gender | | | | |
| Male | 1 | 1 | 1 | 1 |
| Female | 0·90 ^***^ | 1·14 ^***^ | 0·98 ^***^ | 1·00 |
|  | (0·88 – 0·92) | (1·12 – 1·16) | (0·97 – 0·99) | (0·99 – 1·01) |
| Index of multiple deprivation (in quintiles) | | | | |
| 1 (least deprived) | 1 | 1 | 1 | 1 |
| 2 | 1·01 | 0·99 | 1·00 | 1·01 |
|  | (0·98 – 1·04) | (0·96 – 1·01) | (0·98 – 1·02) | (0·99 – 1·03) |
| 3 | 1·01 | 1·00 | 1·00 | 0·99 |
|  | (0·98 – 1·04) | (0·98 – 1·03) | (0·98 – 1·02) | (0·98 – 1·01) |
| 4 | 1·02 | 0·99 | 1·00 | 0·99 |
|  | (0·99 – 1·05) | (0·97 – 1·02) | (0·98 – 1·02) | (0·97 – 1·01) |
| 5 (most deprived) | 0·98 | 1·01 | 1·00 | 1·01 |
|  | (0·95 – 1·02) | (0·98 – 1·04) | (0·98 – 1·02) | (0·99 – 1·03) |
| Smoking status | | | | |
| Non-smoker | 1 | 1 | 1 | 1 |
| Current smoker | 0·96 ^*^ | 0·99 | 0·98 | 1·07 ^***^ |
|  | (0·92 – 1·01) | (0·95 – 1·03) | (0·95 – 1·01) | (1·04 – 1·10) |
| Ex-smoker | 0·94 ^***^ | 0·97 ^***^ | 1·03 ^***^ | 1·06 ^***^ |
|  | (0·92 – 0·96) | (0·95 – 0·99) | (1·01 – 1·04) | (1·05 – 1·08) |
| BMI | 1·00 ^**^ | 1·00 ^***^ | 1·00 ^***^ | 1·00 |
|  | (1·00 – 1·00) | (0·99 – 1·00) | (1·00 – 1·00) | (1·00 – 1·00) |
| Age | 1·00 | 1·00 | 1·00 ^***^ | 1·00 ^***^ |
|  | (1·00 – 1·00) | (1·00 – 1·00) | (1·00 – 1·01) | (0·99 – 1·00) |
| * p<0·1   ** p<0·05   *** p<0·01 | | | | |

Additional results for the relationship between multimorbidity clusters and service use and health outcomes for the entire sample, where non-multimorbid patients are taken as reference. Note that complete-case analyses were applied throughout. In non-multimorbid and multimorbid populations, smoking status has 4.9% and 0.2% missingness respectively; BMI has 21% and 5.1% missingness respectively.

**Table 16 Association between of multimorbidity clusters and NHS service utilisation and mortality (age strata 18-44).**  Coefficients are reported in incidence rate ratios (for negative binomial models) and odds ratios (for logit models) with 95% confidence intervals. Non-multimorbid patients are taken as reference.

|  | **GP consultation** | **Hospitalisation** | **Repeat prescription** | **2-year mortality** | **5-year mortality** |
| --- | --- | --- | --- | --- | --- |
| *Predictors* | *IRR (95% CI)* | *IRR (95% CI)* | *IRR (95% CI)* | *OR (95% CI)* | *OR (95% CI)* |
| (Intercept) | 1.77 ^***^ | 0.11 ^***^ | 0.03 ^***^ | 1.00 ^***^ | 0.99 ^***^ |
|  | (1.67 – 1.88) | (0.10 – 0.13) | (0.03 – 0.03) | (1.00 – 1.00) | (0.99 – 1.00) |
| Multimorbidity clusters | | | | | |
| Non-multimorbid (reference) | 1 | 1 | 1 | 1 | 1 |
| AST,IBS,DEP | 2.54 ^***^ | 1.95 ^***^ | 8.47 ^***^ | 1 | 1.00 ^***^ |
|  | (2.40 – 2.69) | (1.72 – 2.20) | (7.88 – 9.10) | (1.00 – 1.00) | (1.00 – 1.01) |
| PSM,ALC,DEP | 3.12 ^***^ | 2.74 ^***^ | 6.38 ^***^ | 1.01 ^***^ | 1.03 ^***^ |
|  | (2.83 – 3.46) | (2.22 – 3.40) | (5.60 – 7.29) | (1.01 – 1.02) | (1.03 – 1.04) |
| PNC,HL,HYP | 2.91 ^***^ | 4.15 ^***^ | 10.78 ^***^ | 1.01 ^***^ | 1.02 ^***^ |
|  | (2.76 – 3.07) | (3.73 – 4.64) | (10.07 – 11.54) | (1.01 – 1.01) | (1.02 – 1.02) |
| DEP,ANX,PNC | 3.21 ^***^ | 2.18 ^***^ | 10.07 ^***^ | 1.01 ^***^ | 1.01 ^***^ |
|  | (3.07 – 3.36) | (1.97 – 2.40) | (9.50 – 10.68) | (1.01 – 1.01) | (1.01 – 1.02) |
| IBS,DEP,HL | 2.27 ^***^ | 2.07 ^***^ | 5.26 ^***^ | 1 | 1 |
|  | (2.14 – 2.41) | (1.83 – 2.35) | (4.86 – 5.70) | (1.00 – 1.00) | (1.00 – 1.00) |
| Gender | | | | | |
| Male | 1 | 1 | 1 | 1 | 1 |
| Female | 1.96 ^***^ | 2.16 ^***^ | 1.30 ^***^ | 1.00 ^***^ | 1.00 ^***^ |
|  | (1.92 – 1.99) | (2.07 – 2.26) | (1.26 – 1.34) | (1.00 – 1.00) | (1.00 – 1.00) |
| Index of multiple deprivation (in quintiles) | | | | | |
| 1 (least deprived) | 1 | 1 | 1 | 1 | 1 |
| 2 | 0.97 ^**^ | 1.06 ^*^ | 1 | 1 | 1 |
|  | (0.94 – 1.00) | (0.99 – 1.14) | (0.95 – 1.05) | (1.00 – 1.00) | (1.00 – 1.00) |
| 3 | 0.96 ^***^ | 1.20 ^***^ | 1.05 ^*^ | 1 | 1 |
|  | (0.93 – 0.99) | (1.12 – 1.29) | (1.00 – 1.10) | (1.00 – 1.00) | (1.00 – 1.00) |
| 4 | 0.97 ^**^ | 1.19 ^***^ | 1.09 ^***^ | 1 | 1 |
|  | (0.94 – 1.00) | (1.11 – 1.27) | (1.04 – 1.15) | (1.00 – 1.00) | (1.00 – 1.00) |
| 5 (most deprived) | 1.03 ^**^ | 1.28 ^***^ | 1.20 ^***^ | 1 | 1.00 ^**^ |
|  | (1.00 – 1.06) | (1.19 – 1.37) | (1.14 – 1.26) | (1.00 – 1.00) | (1.00 – 1.00) |
| Smoking status | | | | | |
| Non-smokers | 1 | 1 | 1 | 1 | 1 |
| Current smoker | 1.09 ^***^ | 1.22 ^***^ | 1.11 ^***^ | 1.00 ^***^ | 1.00 ^***^ |
|  | (1.07 – 1.11) | (1.16 – 1.28) | (1.07 – 1.15) | (1.00 – 1.00) | (1.00 – 1.00) |
| Ex-smoker | 1.15 ^***^ | 1.20 ^***^ | 1.18 ^***^ | 1 | 1 |
|  | (1.12 – 1.18) | (1.13 – 1.28) | (1.13 – 1.23) | (1.00 – 1.00) | (1.00 – 1.00) |
| BMI | 1.02 ^***^ | 1.01 ^***^ | 1.03 ^***^ | 1 | 1 |
|  | (1.02 – 1.02) | (1.00 – 1.01) | (1.03 – 1.04) | (1.00 – 1.00) | (1.00 – 1.00) |
| Age | 1 | 0.99 ^***^ | 1.02 ^***^ | 1.00 ^***^ | 1.00 ^***^ |
|  | (1.00 – 1.00) | (0.98 – 0.99) | (1.02 – 1.03) | (1.00 – 1.00) | (1.00 – 1.00) |
| ** p<0.1   ** p<0.05   *** p<0.01* | | | | | |

**Table 17 Association between of multimorbidity clusters and NHS service utilisation and mortality (age strata 45-64).**  Coefficients are reported in incidence rate ratios (for negative binomial models) and odds ratios (for logit models) with 95% confidence intervals. Non-multimorbid patients are taken as reference.

|  | **GP consultation** | **Hospitalisation** | **Repeat prescription** | **2-year mortality** | **5-year mortality** |
| --- | --- | --- | --- | --- | --- |
| *Predictors* | *IRR (95% CI)* | *IRR (95% CI)* | *IRR (95% CI)* | *OR (95% CI)* | *OR (95% CI)* |
| (Intercept) | 1.28 ^***^ | 0.04 ^***^ | 0.02 ^***^ | 0.98 ^***^ | 0.93 ^***^ |
|  | (1.17 – 1.39) | (0.03 – 0.05) | (0.01 – 0.02) | (0.97 – 0.98) | (0.92 – 0.94) |
| Multimorbidity clusters | | | | | |
| Non-multimorbid (reference) | 1 | 1 | 1 | 1 | 1 |
| DEP,PNC,ANX | 3.26 ^***^ | 3.22 ^***^ | 8.87 ^***^ | 1.02 ^***^ | 1.04 ^***^ |
|  | (3.16 – 3.37) | (2.98 – 3.48) | (8.58 – 9.17) | (1.02 – 1.02) | (1.04 – 1.04) |
| AST,COPD,PNC | 2.47 ^***^ | 2.32 ^***^ | 5.83 ^***^ | 1.01 ^***^ | 1.01 ^***^ |
|  | (2.37 – 2.58) | (2.09 – 2.57) | (5.59 – 6.08) | (1.00 – 1.01) | (1.01 – 1.02) |
| HYP,DIA,PNC | 2.26 ^***^ | 2.68 ^***^ | 6.07 ^***^ | 1.01 ^***^ | 1.02 ^***^ |
|  | (2.20 – 2.32) | (2.52 – 2.86) | (5.91 – 6.24) | (1.01 – 1.01) | (1.02 – 1.03) |
| IBS,HL,PNC | 2.13 ^***^ | 2.54 ^***^ | 3.68 ^***^ | 1.01 ^***^ | 1.01 ^***^ |
|  | (2.07 – 2.20) | (2.36 – 2.74) | (3.56 – 3.81) | (1.01 – 1.01) | (1.01 – 1.02) |
| ALC,PSM,PNC | 2.45 ^***^ | 2.63 ^***^ | 4.68 ^***^ | 1.04 ^***^ | 1.11 ^***^ |
|  | (2.29 – 2.64) | (2.23 – 3.13) | (4.35 – 5.04) | (1.03 – 1.04) | (1.10 – 1.12) |
| Gender | | | | | |
| Male | 1 | 1 | 1 | 1 | 1 |
| Female | 1.31 ^***^ | 1.11 ^***^ | 1 | 1.00 ^***^ | 0.99 ^***^ |
|  | (1.29 – 1.34) | (1.06 – 1.15) | (0.98 – 1.02) | (1.00 – 1.00) | (0.99 – 0.99) |
| Index of multiple deprivation (in quintiles) | | | | | |
| 1 (least deprived) | 1 | 1 | 1 | 1 | 1 |
| 2 | 0.97 ^**^ | 1.24 ^***^ | 1.03 ^**^ | 1 | 1.00 ^**^ |
|  | (0.95 – 1.00) | (1.16 – 1.32) | (1.00 – 1.07) | (1.00 – 1.00) | (1.00 – 1.01) |
| 3 | 0.97 ^**^ | 1.14 ^***^ | 1.05 ^***^ | 1 | 1.00 ^***^ |
|  | (0.95 – 1.00) | (1.07 – 1.22) | (1.02 – 1.08) | (1.00 – 1.00) | (1.00 – 1.01) |
| 4 | 1.01 | 1.31 ^***^ | 1.17 ^***^ | 1.00 ^**^ | 1.01 ^***^ |
|  | (0.98 – 1.03) | (1.22 – 1.39) | (1.13 – 1.20) | (1.00 – 1.00) | (1.00 – 1.01) |
| 5 (most deprived) | 1.08 ^***^ | 1.27 ^***^ | 1.30 ^***^ | 1.00 ^***^ | 1.01 ^***^ |
|  | (1.05 – 1.11) | (1.19 – 1.37) | (1.26 – 1.34) | (1.00 – 1.01) | (1.01 – 1.02) |
| Smoking status | | | | | |
| Non-smokers | 1 | 1 | 1 | 1 | 1 |
| Current smoker | 1.01 | 1.18 ^***^ | 1.10 ^***^ | 1.01 ^***^ | 1.02 ^***^ |
|  | (0.99 – 1.03) | (1.12 – 1.24) | (1.08 – 1.13) | (1.01 – 1.01) | (1.02 – 1.02) |
| Ex-smoker | 1.11 ^***^ | 1.14 ^***^ | 1.20 ^***^ | 1 | 1.00 ^***^ |
|  | (1.09 – 1.13) | (1.09 – 1.20) | (1.17 – 1.23) | (1.00 – 1.00) | (1.00 – 1.01) |
| BMI | 1.01 ^***^ | 1.01 ^***^ | 1.03 ^***^ | 1.00 ^***^ | 1.00 ^***^ |
|  | (1.01 – 1.02) | (1.00 – 1.01) | (1.03 – 1.04) | (1.00 – 1.00) | (1.00 – 1.00) |
| Age | 1.01 ^***^ | 1.02 ^***^ | 1.04 ^***^ | 1.00 ^***^ | 1.00 ^***^ |
|  | (1.01 – 1.02) | (1.02 – 1.02) | (1.04 – 1.05) | (1.00 – 1.00) | (1.00 – 1.00) |
| ** p<0.1   ** p<0.05   *** p<0.01* | | | | | |

**Table 18 Association between of multimorbidity clusters and NHS service utilisation and mortality (age strata 65-84).**  Coefficients are reported in incidence rate ratios (for negative binomial models) and odds ratios (for logit models) with 95% confidence intervals. Non-multimorbid patients are taken as reference.

|  | **GP consultation** | **Hospitalisation** | **Repeat prescription** | **2-year mortality** | **5-year mortality** |
| --- | --- | --- | --- | --- | --- |
| *Predictors* | *IRR (95% CI)* | *IRR (95% CI)* | *IRR (95% CI)* | *OR (95% CI)* | *OR (95% CI)* |
| (Intercept) | 2.40 ^***^ | 0.06 ^***^ | 0.18 ^***^ | 0.79 ^***^ | 0.46 ^***^ |
|  | (2.11 – 2.74) | (0.04 – 0.08) | (0.16 – 0.20) | (0.77 – 0.81) | (0.44 – 0.48) |
| Multimorbidity clusters | | | | | |
| Non-multimorbid (reference) | 1 | 1 | 1 | 1 | 1 |
| CHD,AF,DIA | 2.36 ^***^ | 3.14 ^***^ | 4.12 ^***^ | 1.08 ^***^ | 1.17 ^***^ |
|  | (2.28 – 2.45) | (2.91 – 3.39) | (3.99 – 4.25) | (1.07 – 1.08) | (1.16 – 1.19) |
| PNC,CHD,DEP | 3.66 ^***^ | 4.46 ^***^ | 7.23 ^***^ | 1.13 ^***^ | 1.30 ^***^ |
|  | (3.47 – 3.85) | (4.01 – 4.96) | (6.94 – 7.55) | (1.11 – 1.14) | (1.28 – 1.32) |
| HL,PSD,IBS | 1.91 ^***^ | 2.14 ^***^ | 2.54 ^***^ | 1.02 ^***^ | 1.02 ^***^ |
|  | (1.86 – 1.96) | (2.01 – 2.27) | (2.47 – 2.61) | (1.01 – 1.02) | (1.01 – 1.03) |
| COPD,AST,PNC | 2.27 ^***^ | 2.27 ^***^ | 3.98 ^***^ | 1.06 ^***^ | 1.16 ^***^ |
|  | (2.18 – 2.36) | (2.08 – 2.48) | (3.84 – 4.12) | (1.05 – 1.07) | (1.14 – 1.17) |
| DEP,PNC,ANX | 2.48 ^***^ | 2.58 ^***^ | 4.39 ^***^ | 1.05 ^***^ | 1.12 ^***^ |
|  | (2.40 – 2.56) | (2.40 – 2.77) | (4.27 – 4.52) | (1.05 – 1.06) | (1.11 – 1.13) |
| HYP,DIA,CKD | 1.76 ^***^ | 1.85 ^***^ | 3.26 ^***^ | 1.02 ^***^ | 1.04 ^***^ |
|  | (1.72 – 1.80) | (1.75 – 1.95) | (3.19 – 3.33) | (1.02 – 1.02) | (1.03 – 1.05) |
| Gender | | | | | |
| Male | 1 | 1 | 1 | 1 | 1 |
| Female | 1.05 ^***^ | 0.91 ^***^ | 0.99 | 0.99 ^***^ | 0.97 ^***^ |
|  | (1.03 – 1.07) | (0.88 – 0.95) | (0.98 – 1.01) | (0.99 – 0.99) | (0.97 – 0.98) |
| Index of multiple deprivation (in quintiles) | | | | | |
| 1 (least deprived) | 1 | 1 | 1 | 1 | 1 |
| 2 | 0.96 ^***^ | 1.01 | 1.02 ^*^ | 1 | 1.01 ^*^ |
|  | (0.94 – 0.99) | (0.96 – 1.07) | (1.00 – 1.05) | (1.00 – 1.01) | (1.00 – 1.01) |
| 3 | 0.96 ^***^ | 1.06 ^**^ | 1.04 ^***^ | 1.01 ^**^ | 1.01 ^**^ |
|  | (0.93 – 0.98) | (1.00 – 1.12) | (1.01 – 1.06) | (1.00 – 1.01) | (1.00 – 1.02) |
| 4 | 0.93 ^***^ | 1.08 ^**^ | 1.09 ^***^ | 1.01 ^***^ | 1.03 ^***^ |
|  | (0.91 – 0.96) | (1.01 – 1.15) | (1.07 – 1.12) | (1.01 – 1.02) | (1.02 – 1.04) |
| 5 (most deprived) | 0.92 ^***^ | 1.26 ^***^ | 1.13 ^***^ | 1.02 ^***^ | 1.04 ^***^ |
|  | (0.90 – 0.95) | (1.18 – 1.35) | (1.10 – 1.16) | (1.01 – 1.03) | (1.03 – 1.05) |
| Smoking status | | | | | |
| Non-smoker | 1 | 1 | 1 | 1 | 1 |
| Current smoker | 0.98 ^*^ | 1.08 ^**^ | 1.02 | 1.03 ^***^ | 1.09 ^***^ |
|  | (0.95 – 1.00) | (1.02 – 1.15) | (0.99 – 1.04) | (1.03 – 1.04) | (1.08 – 1.10) |
| Ex-smoker | 1.06 ^***^ | 1.13 ^***^ | 1.10 ^***^ | 1.01 ^***^ | 1.02 ^***^ |
|  | (1.04 – 1.08) | (1.08 – 1.18) | (1.08 – 1.12) | (1.01 – 1.01) | (1.02 – 1.03) |
| BMI | 1.01 ^***^ | 1.00 ^*^ | 1.02 ^***^ | 1.00 ^***^ | 1.00 ^***^ |
|  | (1.01 – 1.01) | (1.00 – 1.01) | (1.02 – 1.02) | (1.00 – 1.00) | (1.00 – 1.00) |
| Age | 1.01 ^***^ | 1.02 ^***^ | 1.02 ^***^ | 1.00 ^***^ | 1.01 ^***^ |
|  | (1.01 – 1.01) | (1.02 – 1.03) | (1.02 – 1.02) | (1.00 – 1.00) | (1.01 – 1.01) |
| ** p<0.1   ** p<0.05   *** p<0.01* | | | | | |

**Table 19 Association between of multimorbidity clusters and NHS service utilisation and mortality (age strata 85+).**  Coefficients are reported in incidence rate ratios (for negative binomial models) and odds ratios (for logit models) with 95% confidence intervals. Non-multimorbid patients are taken as reference.

|  | **GP consultation** | **Hospitalisation** | **Repeat prescription** | **2-year mortality** | **5-year mortality** |
| --- | --- | --- | --- | --- | --- |
| *Predictors* | *IRR (95% CI)* | *IRR (95% CI)* | *IRR (95% CI)* | *OR (95% CI)* | *OR (95% CI)* |
| (Intercept) | 3.37 ^***^ | 1.54 | 5.47 ^***^ | 0.25 ^***^ | 0.13 ^***^ |
|  | (1.81 – 6.27) | (0.57 – 4.11) | (3.20 – 9.31) | (0.20 – 0.31) | (0.10 – 0.17) |
| Multimorbidity clusters | | | | | |
| Non-multimorbid (reference) | 1 | 1 | 1 | 1 | 1 |
| HF,CHD,AF | 3.00 ^***^ | 3.34 ^***^ | 5.36 ^***^ | 1.27 ^***^ | 1.37 ^***^ |
|  | (2.73 – 3.30) | (2.87 – 3.89) | (4.92 – 5.84) | (1.22 – 1.31) | (1.32 – 1.43) |
| AST,COPD,PNC | 2.65 ^***^ | 2.37 ^***^ | 4.59 ^***^ | 1.17 ^***^ | 1.22 ^***^ |
|  | (2.40 – 2.94) | (2.01 – 2.79) | (4.19 – 5.03) | (1.13 – 1.21) | (1.17 – 1.27) |
| HYP,HL,DIA | 1.78 ^***^ | 1.70 ^***^ | 2.77 ^***^ | 1.07 ^***^ | 1.12 ^***^ |
|  | (1.66 – 1.91) | (1.51 – 1.92) | (2.59 – 2.96) | (1.05 – 1.10) | (1.09 – 1.15) |
| PNC,DEP,CSP | 2.45 ^***^ | 2.07 ^***^ | 4.60 ^***^ | 1.17 ^***^ | 1.25 ^***^ |
|  | (2.26 – 2.65) | (1.81 – 2.38) | (4.26 – 4.96) | (1.13 – 1.20) | (1.21 – 1.30) |
| Gender | | | | | |
| Male |  |  |  |  |  |
| Female | 0.94 ^**^ | 0.86 ^***^ | 1.05 ^**^ | 0.95 ^***^ | 0.93 ^***^ |
|  | (0.90 – 0.99) | (0.80 – 0.93) | (1.01 – 1.09) | (0.93 – 0.97) | (0.91 – 0.95) |
| Index of multiple deprivation (in quintiles) | | | | | |
| 1( least deprived) | 1 | 1 | 1 | 1 | 1 |
| 2 | 0.96 | 1 | 1.04 | 1 | 1 |
|  | (0.90 – 1.02) | (0.90 – 1.11) | (0.98 – 1.10) | (0.97 – 1.02) | (0.98 – 1.03) |
| 3 | 0.93 ^**^ | 1.15 ^***^ | 1 | 1.01 | 1.01 |
|  | (0.87 – 0.99) | (1.04 – 1.28) | (0.94 – 1.06) | (0.99 – 1.04) | (0.99 – 1.04) |
| 4 | 0.89 ^***^ | 0.97 | 0.99 | 1.01 | 1.01 |
|  | (0.83 – 0.96) | (0.87 – 1.09) | (0.93 – 1.05) | (0.98 – 1.03) | (0.98 – 1.04) |
| 5 (most deprived) | 0.90 ^***^ | 1.06 | 1.08 ^**^ | 1.01 | 1.04 ^**^ |
|  | (0.83 – 0.97) | (0.94 – 1.20) | (1.01 – 1.16) | (0.99 – 1.04) | (1.01 – 1.08) |
| Smoking status | | | | | |
| Non smoker |  |  |  |  |  |
| Current smoker | 0.93 | 0.99 | 0.89 ^**^ | 1.05 ^***^ | 1.11 ^***^ |
|  | (0.85 – 1.03) | (0.84 – 1.16) | (0.82 – 0.98) | (1.01 – 1.09) | (1.06 – 1.15) |
| Ex-smoker | 1.06 ^**^ | 1.01 | 1.07 ^***^ | 1.01 | 1.03 ^***^ |
|  | (1.01 – 1.11) | (0.93 – 1.09) | (1.03 – 1.12) | (0.99 – 1.03) | (1.01 – 1.06) |
| BMI | 1 | 0.99 | 1.01 ^***^ | 0.99 ^***^ | 0.99 ^***^ |
|  | (0.99 – 1.00) | (0.99 – 1.00) | (1.00 – 1.01) | (0.99 – 0.99) | (0.99 – 1.00) |
| Age | 0.98 ^***^ | 0.99 ^**^ | 0.98 ^***^ | 1.02 ^***^ | 1.03 ^***^ |
|  | (0.98 – 0.99) | (0.98 – 1.00) | (0.98 – 0.99) | (1.02 – 1.02) | (1.03 – 1.03) |
| ** p<0.1   ** p<0.05   *** p<0.01* | | | | | |

**Figure 5 Cluster profiles for patients aged 18-44 years.** Morbidities are ordered based on the overall prevalence in the population (from most to least). Full list of abbreviations of conditions is defined in Table 1. Cluster labels are based around the three conditions estimated to be most distinctive in the cluster (where the difference between within-cluster prevalence and prevalence in age strata is the largest). Numbers in cells are estimated prevalence of long-term conditions (row) in each cluster (column) and is also visualised by the blue bars. Greater deprivation is defined by categories 4 and 5 of the IMD. The number of repeat prescriptions (at least four times), GP consultations and hospitalisations are measured in one year after January 2012. The number of prescriptions is computed by counting the number of unique BNF codes that were in repeated prescriptions for at least four times in one year.

**Figure 6 Cluster profiles for patients aged 45-64 years.** Morbidities are ordered based on the overall prevalence in the population (from most to least). Full list of abbreviations of conditions is defined in Table 1. Cluster labels are based around the three conditions estimated to be most distinctive in the cluster (where the difference between within-cluster prevalence and prevalence in age strata is the largest). Numbers in cells are estimated prevalence of long-term conditions (row) in each cluster (column) and is also visualised by the blue bars. Greater deprivation is defined by categories 4 and 5 of the IMD. The number of repeat prescriptions (at least four times), GP consultations and hospitalisations are measured in one year after January 2012. The number of prescriptions is computed by counting the number of unique BNF codes that were in repeated prescriptions for at least four times in one year.

**Figure 7 Cluster profiles for patients aged 65-84 years.** Morbidities are ordered based on the overall prevalence in the population (from most to least). Full list of abbreviations of conditions is defined in Table 1. Cluster labels are based around the three conditions estimated to be most distinctive in the cluster (where the difference between within-cluster prevalence and prevalence in age strata is the largest). Numbers in cells are estimated prevalence of long-term conditions (row) in each cluster (column) and is also visualised by the blue bars. Greater deprivation is defined by categories 4 and 5 of the IMD. The number of repeat prescriptions (at least four times), GP consultations and hospitalisations are measured in one year after January 2012. The number of prescriptions is computed by counting the number of unique BNF codes that were in repeated prescriptions for at least four times in one year.

**Figure 8 Cluster profiles for patients aged 85+ years.** Morbidities are ordered based on the overall prevalence in the population (from most to least). Full list of abbreviations of conditions is defined in Table 1. Cluster labels are based around the three conditions estimated to be most distinctive in the cluster (where the difference between within-cluster prevalence and prevalence in age strata is the largest). Numbers in cells are estimated prevalence of long-term conditions (row) in each cluster (column) and is also visualised by the blue bars. Greater deprivation is defined by categories 4 and 5 of the IMD. The number of repeat prescriptions (at least four times), GP consultations and hospitalisations are measured in one year after January 2012. The number of prescriptions is computed by counting the number of unique BNF codes that were in repeated prescriptions for at least four times in one year.

References

1 Schäfer I, von Leitner E-C, Schön G, *et al.* Multimorbidity patterns in the elderly: a new approach of disease clustering identifies complex interrelations between chronic conditions. *PLoS One* 2010; **5**: e15941.

2 France EF, Wyke S, Gunn JM, Mair FS, McLean G, Mercer SW. Multimorbidity in primary care: a systematic review of prospective cohort studies. *Br J Gen Pr* 2012; **62**: e297--e307.

3 Huntley AL, Johnson R, Purdy S, Valderas JM, Salisbury C. Measures of multimorbidity and morbidity burden for use in primary care and community settings: a systematic review and guide. *Ann Fam Med* 2012; **10**: 134–41.

4 Schmidt M, Jacobsen JB, Lash TL, Bøtker HE, Sørensen HT. 25 year trends in first time hospitalisation for acute myocardial infarction, subsequent short and long term mortality, and the prognostic impact of sex and comorbidity: a Danish nationwide cohort study. *BMJ* 2012; **344**: e356.

5 Chen H-Y, Saczynski JS, McManus DD, *et al.* The impact of cardiac and noncardiac comorbidities on the short-term outcomes of patients hospitalized with acute myocardial infarction: a population-based perspective. *Clin Epidemiol* 2013; **5**: 439.

6 Ahlqvist E, Storm P, Käräjämäki A, *et al.* Novel subgroups of adult-onset diabetes and their association with outcomes: a data-driven cluster analysis of six variables. *Lancet Diabetes Endocrinol* 2018; **6**: 361–9.

7 Collerton J, Jagger C, Yadegarfar ME, *et al.* Deconstructing Complex Multimorbidity in the Very Old: Findings from the Newcastle 85$\mathplus$ Study. *Biomed Res Int* 2016; **2016**: 1–15.

8 Whitson HE, Johnson KS, Sloane R, *et al.* Identifying Patterns of Multimorbidity in Older Americans: Application of Latent Class Analysis. *J Am Geriatr Soc* 2016; **64**: 1668–73.

9 Gellert P, von Berenberg P, Zahn T, Neuwirth J, Kuhlmey A, Dräger D. Multimorbidity Profiles in German Centenarians: A Latent Class Analysis of Health Insurance Data. *J Aging Health* 2017; **31**: 580–94.

10 Nguyen QD, Wu C, Odden MC, Kim DH. Multimorbidity Patterns, Frailty, and Survival in Community-Dwelling Older Adults. *Journals Gerontol Ser A* 2018; published online Aug. DOI:10.1093/gerona/gly205.

11 Nylund KL, Asparouhov T, Muthén BO. Deciding on the Number of Classes in Latent Class Analysis and Growth Mixture Modeling: A Monte Carlo Simulation Study. *Struct Equ Model A Multidiscip J* 2007; **14**: 535–69.

12 Prados-Torres A, Calderón-Larrañaga A, Hancco-Saavedra J, Poblador-Plou B, van den Akker M. Multimorbidity patterns: a systematic review. *J Clin Epidemiol* 2014; **67**: 254–66.

13 Lin J. Divergence measures based on the Shannon entropy. *IEEE Trans Inf theory* 1991; **37**: 145–51.

14 Altman DG. Practical statistics for medical research. CRC press, 1990.

1. Overall (relative) entropy (denoted by E) is computed by

   $E=1+\frac{1}{Nln\left( k \right)}\left[ \sum_{i=1}^{N} \sum_{k=1}^{K} \begin{aligned} P\left( C=k | \boldsymbol{M}_{i} \right)\ln\left( P\left( C=k | \boldsymbol{M}_{i} \right) \right) \end{aligned} \right]$, where k denotes the class number, i denotes individual, **M** denotes the morbidity pattern (a vector of length 38) and C denotes the class assignment. Individuals are assigned to the class where the posterior probability of being in this class $P\left( C=k | \boldsymbol{M}_{i} \right)$ is the highest. Higher values (closer to 1) indicates good class separation, i.e. less “fuzziness” between classes. [↑](#footnote-ref-1)
